# The effectiveness of the chronic disease management for hypertension: A systematic review and meta-analysis

**DOI:** 10.1097/MD.0000000000042455

**Published:** 2025-07-25

**Authors:** Zeyu Li, Jiahua Wu, Weidan Yuan, Danchun Lan, Ruchun Chang, Ziyong Li, Liming Lu, Peiming Zhang

**Affiliations:** a Gaozhou Traditional Chinese Medicine Hospital, Maoming, China; b Foshan Hospital of Traditional Chinese Medicine Affiliated to Guangzhou University of Chinese Medicine (The Eighth Clinical Medical College of Guangzhou University of Chinese Medicine), Chancheng District, Foshan, China; c Clinical Research and Big Data Laboratory, South China Research Center for Acupuncture and Moxibustion, Medical College of Acu-Moxi and Rehabilitation, Guangzhou University of Chinese Medicine, Panyu District, Guangzhou, China.

**Keywords:** chronic disease management, hypertension, intervention effectiveness, meta-analysis, systematic review

## Abstract

**Background:**

Chronic disease management (CDM) is becoming more crucial in optimizing the management of hypertension and related risk control while enhancing the self-efficacy of patients. However, there is a lack of systematic summaries on the subject. This study aimed to provide synthesized evidence on the effectiveness of interventions and the quality of evidence for the implementation of CDM for hypertension.

**Methods:**

The protocol of this study was registered on PROSPERO (CRD42023472066). Traditional (randomized control trial)/cluster randomized controlled trials were searched on Medical Literature Analysis and Retrieval System Online (database) (via PubMed), Excerpta Medica dataBASE, and Cochrane from inception until September 30th, 2023 (updated to March 31st, 2025), including comparisons of CDM with blank control/usual care (BC/UC), or comprehensive CDM with only self-management support (SMS). We applied data pooling using a fixed/random effect model to account for statistical heterogeneity. Effect sizes for continuous and categorical outcomes were expressed as mean difference (MD) or standardized MD (SMD), and risk ratios with 95% confidence intervals (CIs).

**Results:**

We included 10 randomized control trials and 6 cluster randomized controlled trials enrolling 9109 patients for systematic review and meta-analysis. Compared with BC/UC, CDM was more conducive to diastolic blood pressure control after 3 to 4 months (MD with 95% CI −6.46 [−8.23, −4.69], moderate certainty). Comprehensive CDM was better for cardiovascular disease risk control than BC or only SMS after 6 months (SMD with 95% CI −1.82 [−3.06, −0.58], moderate certainty). Concerning self-efficacy improvement, comprehensive CDM was more beneficial than BC/UC (SMD with 95% CI 0.73 [0.45, 1.01] and 1.01 [0.72, 1.30] after 6 weeks and 3 months, moderate certainty).

**Conclusion:**

In summary, CDM improved hypertension control especially diastolic blood pressure after a 3-month follow-up, and comprehensive CDM instead of only SMS favored cardiovascular disease risk control after a 6-month follow-up. The evidence remains to be strengthened via further large-scale, targeted verification, and to be systematically accumulated to provide a basis for network comparisons among CDM models.

## 1. Introduction

Hypertension poses a global health challenge. Its prevalence (especially in low- and middle-income countries) and risks (especially cardiovascular morbidity and mortality) are rising despite controlled global mean blood pressure (BP) via antihypertensive medications in the past 4 decades.^[1,2]^ Differences in knowledge, lifestyle, and self-monitoring of BP and sodium intake provide some explanation of the regional heterogeneity in hypertension prevalence and control effectiveness.^[3]^

Thus effective, proactive, and comprehensive chronic disease management (CDM) strategies are urgently needed.^[4]^ CDM, defined as an intervention for chronic condition management or prevention using a systematic care approach and potentially multiple treatment modalities,^[5]^ covers primary to tertiary preventions of hypertension. The Chronic Care Model, a well-known CDM model, developed in the USA after extensive review,^[6]^ has 6 elements for chronic condition care improvement within the integration context of the community, health care system, and provider organization^[7]^: Delivery System Design (DSD), Self-Management Support (SMS), Decision Support (DS), Clinical Information Systems, Community Resources (CR), and Health Care Organization (HCO).

Evidence-based CDM is crucial for curbing hypertension burden, reducing costs, and mitigating risks, requiring an efficient combination of hypertension CDM models. However, previous reviews of CDM interventions’ clinical effectiveness for hypertension lack systematicity, emphasizing self-management effectiveness and neglecting other CDM models, like a 2017 systematic review (SR) about mobile health-based self-management.^[8]^ Previous research also focused more on specific intervention techniques or forms, as in a 2016 SR about digital intervention for hypertension self-management.^[9]^ There has been a lack of qualitative and quantitative summarization at the model level, causing a deficiency in systematic evidence regarding the combination and widespread application of hypertension CDM models. Hence, a preliminary assessment of evidence in this area is urgently required. Given the published amount of experimental evidence, this study included 4 models (except CR and HCO) and conducted a SR and meta-analysis (MA) comparing CDM with blank control/usual care (BC/UC) or comprehensive CDM with only SMS in health practice.

## 2. Methods

### 2.1. Protocol and registration

This research was performed in accordance with the Preferred Reporting Items for Systematic Reviews and Meta-Analyses (PRISMA) checklist (File S1, Supplemental Digital Content, https://links.lww.com/MD/P458).^[10]^ The protocol for this SR was pre-registered with International Prospective Register of Systematic Reviews (PROSPERO) (CRD42023472066). Ethics approval is not required for SR and all the articles included in this study had obtained the approval of the ethics committee.

### 2.2. Data source

In this study, a literature search was performed applying 3 electronic medical databases Medical Literature Analysis and Retrieval System Online via PubMed, Excerpta Medica dataBASE, and Cochrane Central Register of Controlled Trials with the inclusion date from database inception until September 30th, 2023 (updated to March 31st, 2025) and without language restriction. Retrievals were conducted according to PRISMA guidelines.^[11]^ The search terms included “hypertension” or “high blood pressure,” and “chronic disease management,” and “trials” (the search strategy was provided in File S2 Section 1, Supplemental Digital Content, https://links.lww.com/MD/P459).

### 2.3. Study selection

The inclusion criteria were predefined for eligible studies as follows: (1) participants: hypertension (BP exceeds 140/90 mm Hg, or exceeds 130/80 mm Hg combined with other cardiovascular and cerebrovascular risk diseases such as diabetes or taking any antihypertensive drug); (2) interventions: CDM methods which could be abstracted and classified according to Chronic Care Model except for CR and HCO (File S2 Section 2, Supplemental Digital Content, https://links.lww.com/MD/P459); (2) comparisons: BC, UC, etc; (3) study design: traditional or cluster randomized control trial (RCT/CRT), given that many of these studies were oriented to real environments. If more than 1 study evaluated the same population with the same outcome, only the larger study was included. The exclusion criteria were (1) reviews, case reports, protocols, short communications, personal opinions, letters, posters, conference abstracts, or laboratory research; (2) those without reporting sufficient data, and efforts to contact the authors failed; or (3) those applied outdated diagnostic or screening criteria for hypertension (BP exceeds 130/80 mm Hg); (4) those did not involve participants with CDM.

The duplicated studies were excluded initially via Endnote 9.3.3 first. The remaining articles were screened for relevance in the title and abstract, and then any article with a relevant abstract or without an abstract was selected for full-text review (by Danchun Lan and Ruchun Chang). A third reviewer (Liming Lu) adjudicated disagreements when necessary.

### 2.4. Data extraction

Two of the reviewers (Danchun Lan and Ruchun Chang) collected information from the included studies independently and cross-checked the information to ensure the integrity of the contents. Discrepancies were resolved by discussion with a third participant (Peiming Zhang). The gathered data included author, publication year, country or region, population (target disease), gender, age, sample size (SS), intervention, comparison, and outcome.

### 2.5. Quality assessment

#### 2.5.1. Assessment of risk of bias (ROB)

The ROB was assessed independently by 2 of us (Jiahua Wu and Weidan Yuan) and in duplicate applying the Cochrane Risk of Bias 2.0 (ROB2) tool^[12]^ in the following domains: randomization process (D1a), timing of identification or recruitment of participants (D1b), deviations from intended interventions (D2), missing outcome data (D3), measurement of the outcome (D4), and selection of the reported result (D5), 5 domains for RCTs (except for D1b) and 6 for CRTs. Each domain was rated as “low,” “some concerns” or “high,” and the overall ROB for each trial was based on the highest risk attributed to any 1 domain.

#### 2.5.2. Assessment of certainty of evidence

The certainty of evidence for each outcome was assessed by another 2 of us (Zeyu Li and Peiming Zhang) applying the Grading of Recommendations, Assessment, Development, and Evaluation (GRADE) approach.^[13]^ In keeping with GRADE methods, the terminology was used consistent with the overall certainty of evidence, including high, moderate, low, and very low certainty.

### 2.6. Data analysis

#### 2.6.1. Summary measures and synthesis of results

This study finally included 5 outcomes for data quantitative synthesis, that is, BP control rate, change in systolic blood pressure (SBP), diastolic blood pressure (DBP), cardiovascular disease (CVD) risk, and BP control self-efficacy. (1) BP control: for simple hypertensives, BPs were controlled below 140/90 mm Hg; for uncontrolled hypertensives at baseline or those with other cardio-cerebrovascular risks, BPs were finally controlled. (2) BP change: difference in home/resting SBP/DBP between endpoint and baseline. (3) CVD risk: measured by New Zealand Risk Score^[14]^ and Framingham Risk Score.^[15]^ New Zealand Risk Score calculates the 5-year risk for a first occurrence of a fatal or nonfatal cardiovascular event by gender, age, diabetes, smoking status, BP, and cholesterol levels, measuring range divided into 4 sections: mild (≤10%), moderate (10–15%), high (15–20%), very high (>20%). Framingham Risk Score calculates 10-year global CVD risk score based on Cox model involving age, cholesterol levels, SBP, smoking, and diabetes as the independent variables. (4) Self-efficacy: measured by subscale of Hypertension Self-Care Profile,^[16]^ with 20-item, 4-point Likert rating, high score indicating good self-efficacy in hypertension self-care.

Data analysis applied meta module in RevMan Manager 5.4.1. Considering multiple observation time points in most relevant studies, different assessment time points were included for subgroup analyses to understand short- and long-term CDM effects. SBP/DBP changes were pooled as mean difference (MD), while the changes in CVD risk and self-efficacy as standardized MD (SMD), using inverse variance weighting (Hedges 1981), with Mantel–Haenszel method (Greenland & Robins 1985, Robins et al 1986) for sensitivity analysis. BP control rate was pooled as risk ratio (RR) with Mantel–Haenszel method and inverse variance weighting for sensitivity analysis. All pooled effect sizes (ESs) had 95% confidence intervals (CIs). If change value was need for the outcome but only an endpoint value provided, it would be converted to the change value utilizing the baseline data setting R 0.5, and if medians and interquartile ranges (IQR) were reported instead of mean and standard deviation, normality in data distribution would be assumed and IQR would be converted to standard deviations by dividing the IQR by 1.35.

Trial heterogeneity was assessed by I^2^ statistic, Cochran *Q* test (for the between-study variance τ^2^, with *P* value at significance level *α* = 0.1), and forest plot. When there was significant heterogeneity based on the fixed effect model (FEM), the random effect model (REM) would be used to combine the ES. If REM still can not explain heterogeneity well, discussion of clinical heterogeneity and methodological heterogeneity would be tried, such as conducting some other subgroup analyses, meta-regression (using R 4.1.3) and some sensitivity analyses.

#### 2.6.2. Publication bias analysis

Publication bias analysis was planned when the study amount was enough (>10 studies), visualized via the funnel plot.

## 3. Results

### 3.1. Overview of the included studies

**Figure 1.**
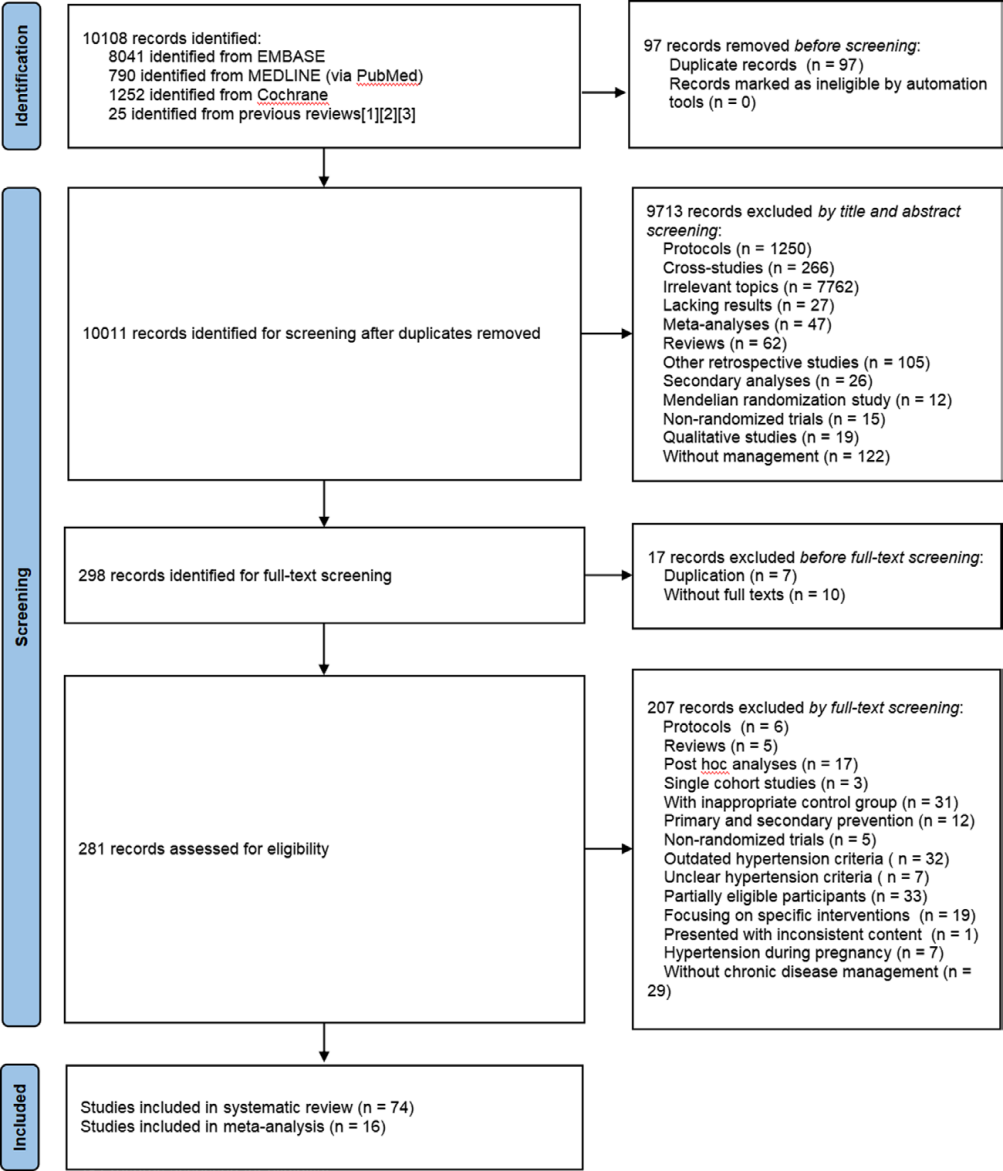
PRISMA flowchart. CENTRAL = Cochrane Central Register of Controlled Trials, EMBASE = Excerpta Medica dataBASE, MEDLINE = Medical Literature Analysis and Retrieval System Online, PRISMA = Preferred Reporting Items for Systematic Reviews and Meta-analyses, PubMed = Public/Publisher MEDLINE (NLM journal articles database). *Twenty-five articles came from 3 reviews with reference detailed in File S3, Supplemental Digital Content, https://links.lww.com/MD/P460.

The retrieval resulted in 10,108 records from the 3 databases, with the inclusion of 74 studies for SR (involving 52 RCTs and 22 CRTs, File S2 Section 3, Supplemental Digital Content, https://links.lww.com/MD/P459), of which 16 for MA (involving 10 RCTs and 6 CRTs),^[17–32]^ with SSs ranging from 43 to 8642 (totally 9109 participants involved in the MA), age ranging from 35 to 90 years, gender mixed, covering totally 25 countries, including USA (28 trials), China (14 trials), UK (4 trials), Spain (3 trials), 5 countries with 2 trials, and the others with 1 trial respectively. All trials applied SMS, 35 trials applied DS or DSD respectively, and 37 applied Clinical Information Systems; considering model combinations, 25 trials applied two-model CDM, 32 trials applied three-model CDM, and 5 applied four-model CDM. The PRISMA flow diagram shows the search history (Fig. 1).

### 3.2. ROB results of the included studies for MA

**Figure 2.**
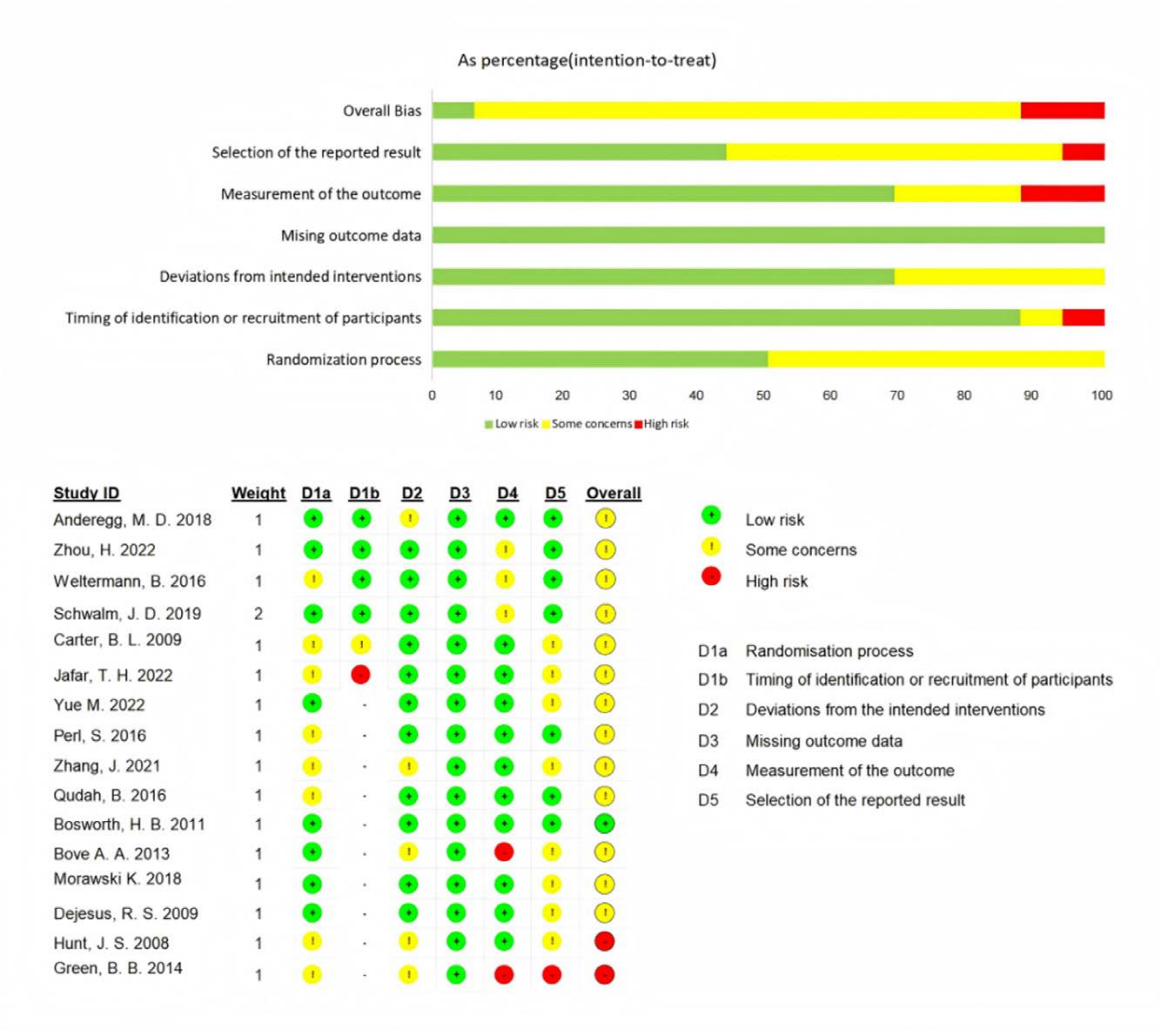
Risk of bias. *Note*: the results produced via ROB2 with 5 domains for RCTs and 6 for CRTs. The upper sub-figure demonstrates the summary ROB, while the lower demonstrates the individual ROB. CRT = cluster randomized control trial, RCT = randomized control trial, ROB = risk of bias.

Figure 2 summarized ROB assessment that identified the main limitations as high risk of D1b (6.3%), D4 (12.5%), and D5 (6.3%), and some concerns of D1a (50.0%), D1b (6.3%), D2 (31.3%), D4 (18.8%), and D5 (50.0%).

### 3.3. Meta-analysis

#### 3.3.1. BP control rate

**Figure 3.**
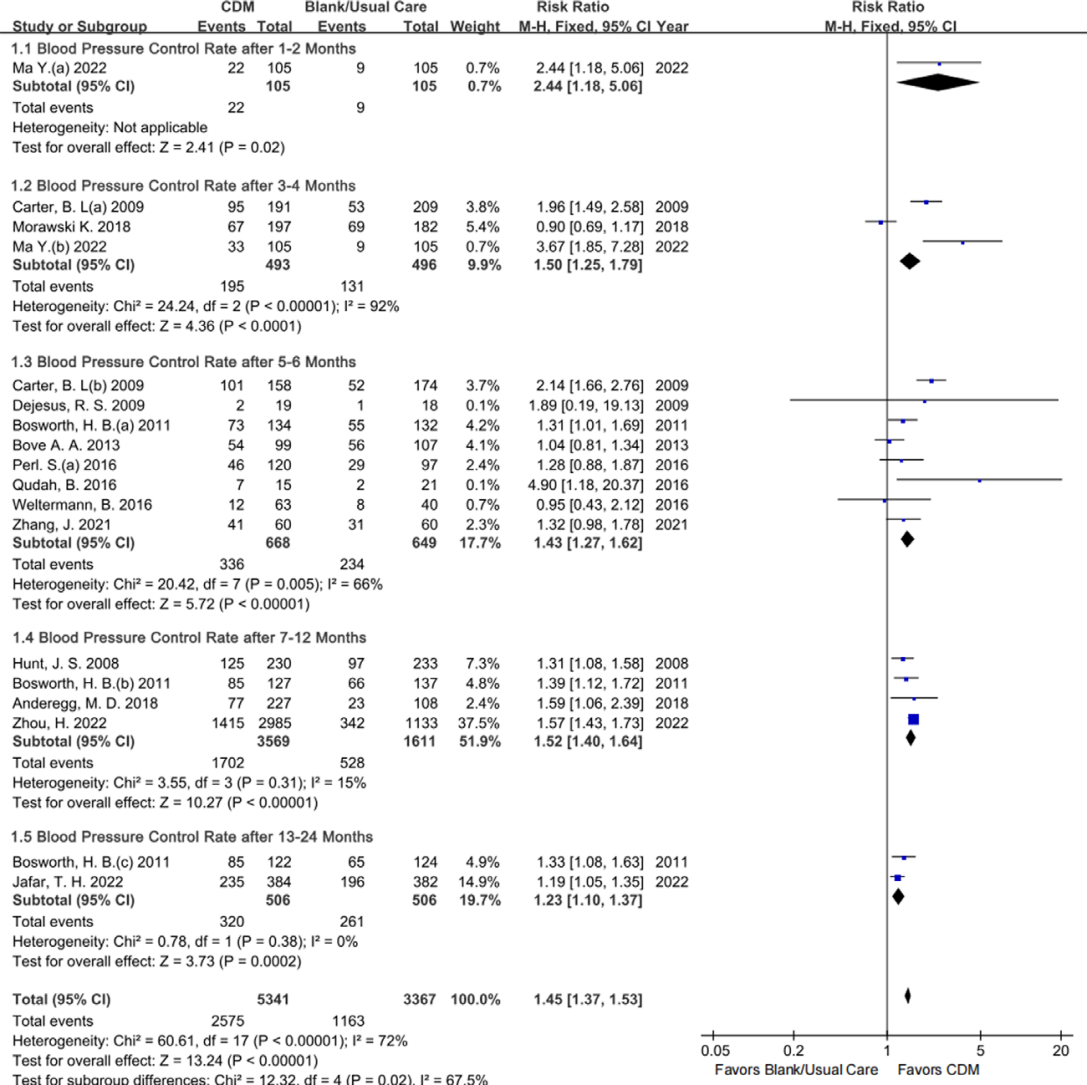
Forestplot of the BP control rate by follow-up time point. BP = blood pressure.

For the BP control rate, 1, 3, 8, 4, and 2 studies were directly pooled by follow-up timepoint of 1 to 2 months, 3 to 4 months, 5 to 6 months, 7 to 12 months, and 13 to 24 months respectively to show its effectiveness characteristics in and between subgroups (Fig. 3). Compared with BC/UC, CDM was more conducive to BP control, and the significance of its effect was more stable after 6 months (RR with 95% CI based on M–H method and FEM: after 7 to 12 months 1.52, 1.40 to 1.64, low certainty; after 13 to 24 months 1.23, 1.10 to 1.37, low certainty), without heterogeneity (I^2^ = 15%, Chi^2^ = 3.55, *P* = .31>0.1 and I^2^ = 0%, Chi^2^ = 0.78, *P* = .38>0.1). The sensitivity analyses based on the M–H with REM or applying the IV method provided similar results (File S2 Figures S1–S3, Supplemental Digital Content, https://links.lww.com/MD/P459). The exclusion of Morawski K 2018 (intervention without DSD) partially explained the heterogeneity in this outcome after 3 to 4 months applying REM and either M–H method (RR 2.48, 1.36–4.52, I^2^ = 65%, Chi^2^ = 2.85, *P* = .09 < 0.1) or IV method (RR 2.47, 1.37–4.46, I^2^ = 64%, Chi^2^ = 2.76, *P* = .10).

#### 3.3.2. Change in SBP

**Figure 4.**
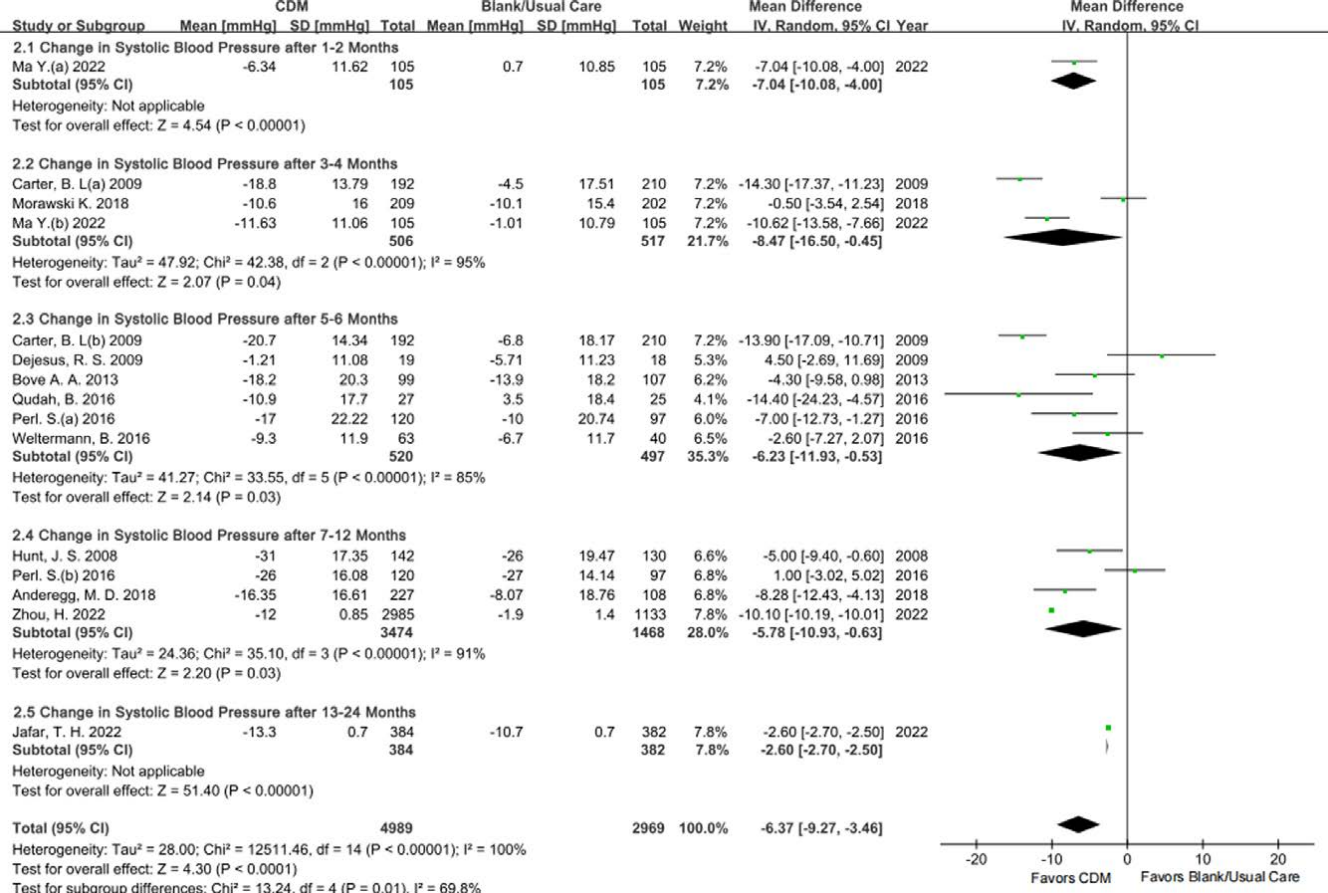
Forestplot of the change in SBP by follow-up time point. SBP = systolic blood pressure.

For the change in SBP, 1, 3, 6, 4, and 1 studies were directly pooled by follow-up times of 1 to 2 months, 3 to 4 months, 5 to 6 months, 7 to 12 months, and 13 to 24 months respectively to show its effect characteristics in and between subgroups (REM in Fig. 4, and FEM in File S2 Figure S4, Supplemental Digital Content, https://links.lww.com/MD/P459). Compared with BC/UC, CDM was more conducive to SBP control according to the subtotal ES with moderate certainty but high heterogeneity at the endpoints spanning 3 to 6 months. The exclusion of Morawski K 2018 explained partially the heterogeneity of the endpoint after 3 to 4 months, subgroup analysis according to comparison difference (Dejesus, R. S. 2009, Per. S 2016, and Weltermann, B. 2016 as 1 of the 2 subgroups) explained partially the heterogeneity of 5 to 6 months, and country difference (Perl, S.. 2016 in Austria and Zhou, H. 2022 in China) explained partially the heterogeneity of 7 to 12 months.

#### 3.3.3. Change in DBP

**Figure 5.**
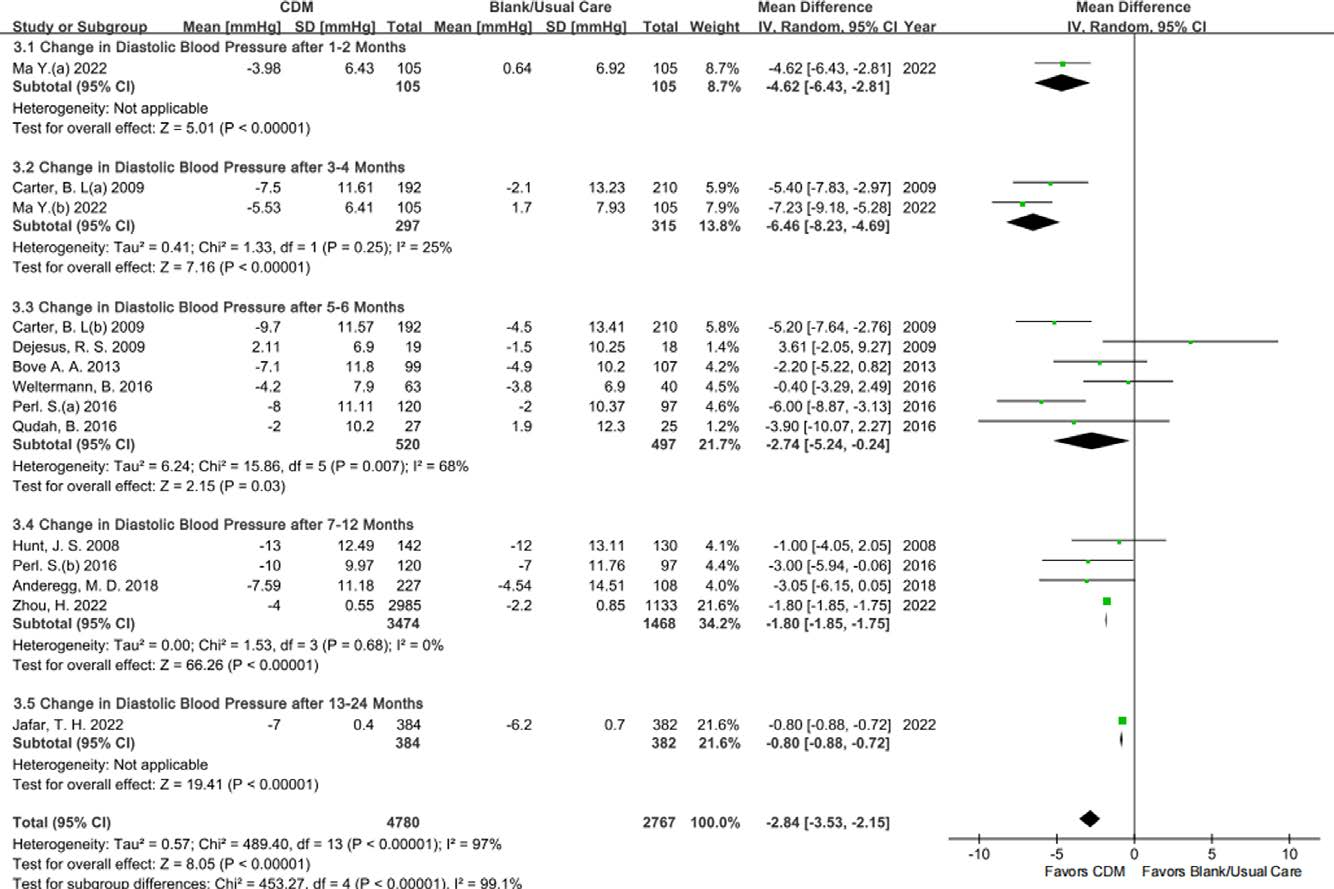
Forestplot of the change in DBP by follow-up time point. DBP = diastolic blood pressure.

For the change in DBP, 1, 2, 6, 4, and 1 studies were directly pooled by follow-up times of 1 to 2 months, 3 to 4 months, 5 to 6 months, 7 to 12 months, and 13 to 24 months respectively to show its effect characteristics in and between subgroups (REM in Fig. 5, and FEM in File S2 Figure S5, Supplemental Digital Content, https://links.lww.com/MD/P459). Compared with BC/UC, CDM was more conducive to DBP control according to the subtotal ES. This outcome after 3 to 4 months and 7 to 12 months showed the advantage of CDM (MD with 95% CI: after 3 to 4 months −6.46, −8.23 to −4.69, moderate certainty; after 7 to 12 months −1.80, −1.85 to −1.75, low certainty) with respectively the lower (I^2^ = 25%, Chi^2^ = 1.33, *P* = .25* > *0.1) and the zero heterogeneity (I^2^ = 0%, Chi^2^ = 1.53, *P* = .68* > *0.1). Involving the studies with follow-up time points between 5 to 6 months, the meta-regression showed the positive effectiveness for CDM more in the studies with larger SSs (File S2 Figure S6, Supplemental Digital Content, https://links.lww.com/MD/P459), indicating that SS was the potential heterogeneity source.

#### 3.3.4. Change in CVD risk

**Figure 6.**
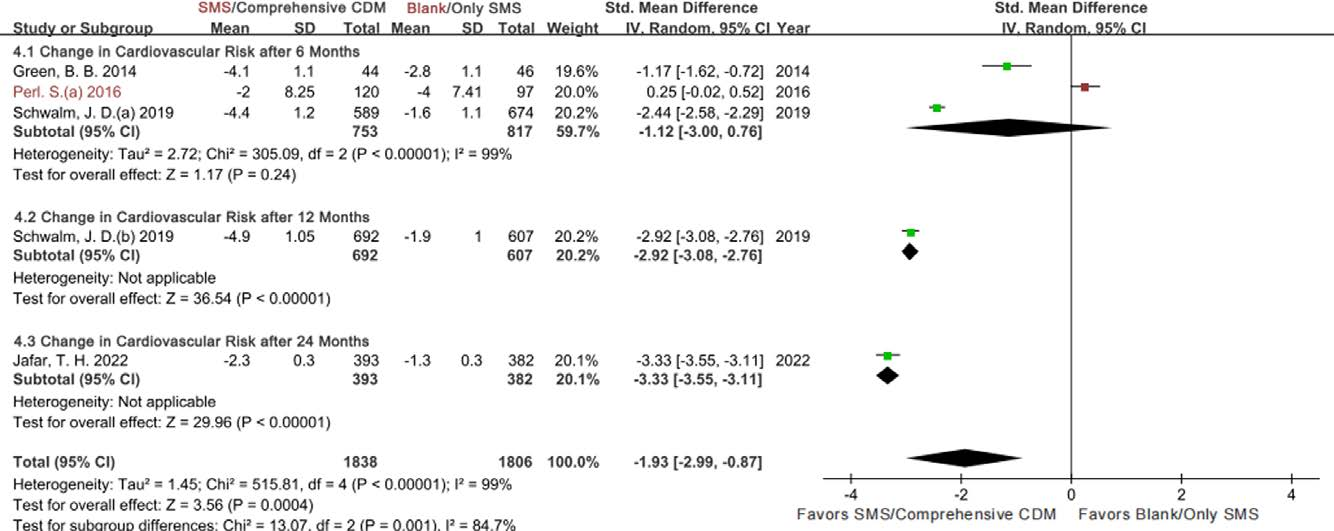
Forestplot of the change in CVD risk by follow-up time point. CVD = cardiovascular disease.

For the change in CVD risk, 3, 1, and 1 studies were directly pooled by 6-month, 12-month, and 24-month follow-up respectively to show its effect characteristics in and between subgroups (REM in Fig. 6, and FEM in File S2 Figure S7, Supplemental Digital Content, https://links.lww.com/MD/P459). Compared with BC or only SMS, SMS/comprehensive CDM was more conducive to CVD risk control after 6 months according to the total ES (SMD with 95% CI: −1.12, −3.00 to −0.76) but with a high heterogeneity (I^2^ = 99%, Chi^2^ = 305.09, *P* < 0.1); although ES of the study Perl, S. 2016 (SMS vs BC) impacted on the precise of point and interval estimates, its exclusion explained heterogeneity scarcely (ES: −1.82, −3.06 to −0.58, I^2^ = 96%, Chi^2^ = 27.66, *P*<0.1, moderate certainty). Merely 1 study (Schwalm. J. D.(b) 2019) was pooled for the 12-month follow-up comparison, indicating comprehensive CDM better than BC (ES: −2.92, −3.08 to −2.76, high certainty).

#### 3.3.5. Change in BP control self-efficacy

**Figure 7.**
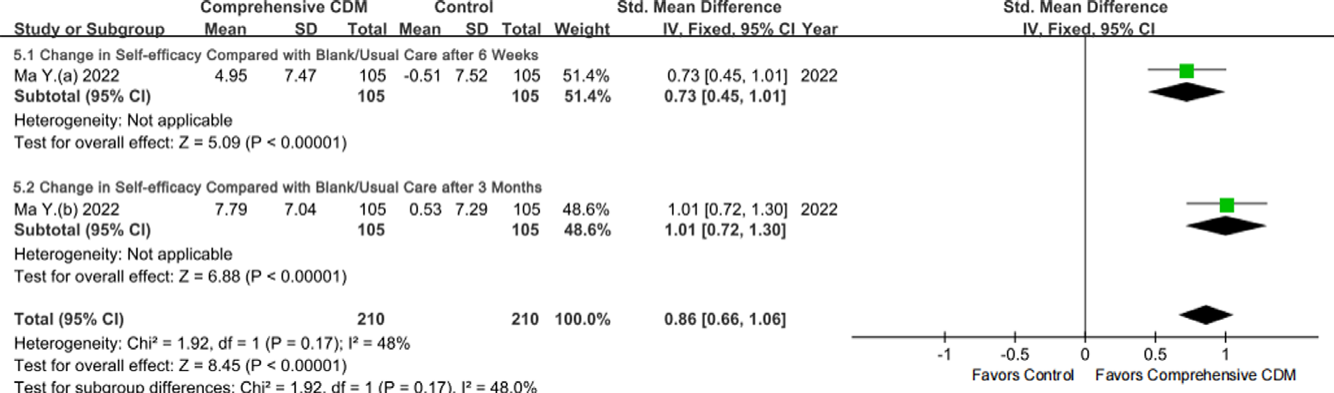
Forestplot of the change in blood pressure control self-efficacy by follow-up time point.

For the change in BP control self-efficacy, not sufficient studies provided ES for quantitative synthesis (1 study covering 2 time points, Fig. 7 or File S2 Figure S8, Supplemental Digital Content, https://links.lww.com/MD/P459). Compared with BC/UC, comprehensive CDM was more conducive to self-efficacy improvement according to the total ES (SMD with 95% CI: after 6 weeks 0.73, 0.45–1.01, moderate certainty; after 3 months: 1.01, 0.72–1.30, moderate certainty).

Supplementary result information is in File S2 4, Supplemental Digital Content, https://links.lww.com/MD/P459 and GRADE assessment is in File S2 Section 4.3–4.4, Supplemental Digital Content, https://links.lww.com/MD/P459.

### 3.4. Publication bias

Although the studies included were insufficient in each comparison, the funnel plots were still provided in File S2 Figure S9 to S13, Supplemental Digital Content, https://links.lww.com/MD/P459 to show the tendency.

## 4. Discussion

### 4.1. Overview

This SR and MA found that the CDM resulted in better BP control compared to BC/UC significantly after 3 months, especially DBP, and CVD risk could be controlled better via comprehensive CDM than only SMS after 6 months. Moreover, subgroup analysis and meta-regression showed the comparison and country difference and SS might contribute to the heterogeneity. Although the amount of evidence was still insufficient, the moderate certainty globally allowed the results of this study to provide valuable information for clinical practice.

### 4.2. Source of heterogeneity

For SBP change, the exclusion of Morawski K. 2018 partly explained heterogeneity of endpoint after 3 to 4 month perhaps owing to DSD absent from the intervention. Unlike other studies, this study set a smartphone application with medication as intervention group without offline interaction, which might limit the effectiveness.^[27]^ Comparison differences (SMS vs BC or comprehensive CDM vs UC/BC) partially explained the heterogeneity of 5 to 6 months, and CDM posed more effectiveness than SMS for SBP change based on ES tendency. Additionally, lower heterogeneity of 7 to 12 month SPG change in US and China^[17,20,31]^ perhaps related to the country or region’s ability of medium- and long-term health management on larger scientific research cohorts. For DBP change, larger SS showed more consistent effectiveness. Inconsistent point estimates of comprehensive CDM versus only SMS may be due to management component differences,^[23,30]^ highlighting the importance of DS.

### 4.3. Comparison with previous relevant reviews

Previous reviews focused on mobile techniques as intervention carriers and community-based nurse-led self-management. One study combined narrative synthesis and MA, pooling BP reduction results and reviewing economic evaluations, showing effectiveness of mHealth SMS in BP control, self-management, and medication adherence.^[33]^ One SR and MA showed interactive digital interventions reduced SBP and DBP compared with UC, but without multiple assessment time points.^[9]^ Both studies left intervention sustainability and long-term effectiveness uncertain. Another study found specially trained nurses might be better than physicians in educating patients on BP self-management in community settings.^[8]^ Similarily, in our study, the common SMS application in all studies included indicated self-management as a key focus of health policies for CDM in many countries, and DSD multidisciplinary teams most often consist of physician–pharmacist–nurse.^[34–36]^ This study provided a deeper review of previous clinical evidence from the CDM model perspective rather than specific intervention forms or carriers, demonstrating the ability of multi-dimensional CDM to control hypertension and risks within at least 2 years.

### 4.4. Strengths and limitations

This study showed some strengths and limitations. It was the first in this topic to conduct a multi-time point outcome MA to explore the trend of short- and long-term effectiveness of CDM on hypertension. In addition, this study applied multi-angle subgroup analyses, sensitivity analyses, and meta-regression to conduct an in-depth discussion on the sources of heterogeneity with data mining. However, due to insufficient literature on each outcome, there was considerable heterogeneity in study design (mixing RCTs and CRTs). Although the SS for some outcome comparisons is large, the evidence credibility remains to be further examined. Acknowledged, too little literature was included on CVD risk so more evidence needs to be accumulated in future to clarify trends and currently unexplained clinical heterogeneity.

### 4.5. Research and clinical implementation

First, SMS alone perhaps does not control BP and its risks well, thus comprehensive CDM is necessary. Therefore, the results of this study may provide evidence of the effectiveness of CDM at multiple assessment time points for future multidisciplinary hypertension management. Secondly, the MA indicated that CDM required a long-term process, and needed to be sustained for at least half a year to achieve expected CVD risk control. Hence, future CDM strategy research on BP and risks needs to consider extension of the follow-up time window and the cost-benefit balance of public health. Thirdly, multi-center, large-sample studies across countries/regions organized by scientifically experienced units on this topic may help enhance evidence credibility in time and space and enable learning from each other’s CDM strategy advantages. In addition, Future research should focus more on patient-centered outcomes for hypertensives, including self-efficacy. What is more, the DS-added comprehensive CDM has shown potential. Finally, studies on combining different CDM components and population stratification are still needed. In future, component-level comparison of different CDM strategies in clinical research may be carried out, or clinical big data mining for simulation and efficient comparison may be conducted, or network MA may be performed when there were sufficient studies.

## 5. Conclusions

In conclusion, the CDM improved hypertension control, and the comprehensive CDM (especially DS added in) instead of only SMS favored CVD risk control. Although in our view, the evidence is not yet robust enough and longer-term effectiveness, sustainability, and the active elements of CDM remain to be clarified, appropriate CDM may be recommended to hypertensive populations in clinical practice since its effectiveness is certain. Of course, how to personalize more efficient evidence-based CDM protocols is the main direction for future efforts.

### Acknowledgments

The authors would like to thank M.D. Mulin Luo (Shenzhen Longgang District Central Hospital, Shenzhen, Guangdong, China) for his assistance with literature information collection and management.

## Author contributions

**Conceptualization:** Peiming Zhang, Liming Lu, Ziyong Li.

**Data curation:** Jiahua Wu, Weidan Yuan.

**Formal analysis:** Jiahua Wu.

**Investigation:** Danchun Lan, Ruchun Chang.

**Methodology:** Liming Lu, Peiming Zhang, Zeyu Li.

**Project administration:** Peiming Zhang.

**Supervision:** Liming Lu.

**Writing – original draft:** Zeyu Li.

**Writing – review & editing:** Liming Lu, Peiming Zhang.

Supplemental Digital Content is available for this article (https://links.lww.com/MD/P461).

## Supplementary Material
