# Supplementary material for: The effectiveness of the chronic disease management for hypertension: A systematic review and meta-analysis
Source: Medicine (Baltimore). 2025 Jul 25;104(30):e42455. doi: 10.1097/MD.0000000000042455 (PMC12303511; doi:10.1097/MD.0000000000042455)
Supplement: Supplementary file 2 [file medi-104-e42455-s002.pdf]

The effectiveness of the chronic disease management for hypertension: a systematic review and meta-analysis

Supplementary File

CONTENTS

1 Retrieval Strategy

2 Identification and Classification of Chronic Disease Management

3 Included Literature

4 Supplementary Results of Meta Analysis

4.1 Forestplots

4.2 Funnel Plots

4.3 GRADE Results - Evidence Profile

4.4 GRADE Results - Summary of Finding Table

1 Retrieval Strategy

(1) Medline (via PubMed, totally 790, update to March 31st, 2025)

((((((Hypertension[MeSH Terms]) OR (Blood Pressure, High[Title/Abstract])) OR (Blood Pressures, High[Title/Abstract])) OR (High Blood Pressure[Title/Abstract])) OR (High Blood Pressures[Title/Abstract])) AND ((((Disease Management[MeSH Terms]) OR (Disease Management*[Title/Abstract])) OR (Management, Disease[Title/Abstract])) OR (Managements, Disease[Title/Abstract]))) AND (((((Prospective Studies[MeSH Terms]) OR (Prospective Stud*[Title/Abstract])) OR (Studies, Prospective[Title/Abstract])) OR (Study, Prospective[Title/Abstract])) OR ((((((Randomized Controlled Trials as Topic[MeSH Terms]) OR (Randomized Controlled Trial[Publication Type])) OR (Clinical Trials, Randomized[Title/Abstract])) OR (Trials, Randomized Clinical[Title/Abstract])) OR (Controlled Clinical Trials, Randomized[Title/Abstract])) OR (Randomly[Title/Abstract]))) AND ("1967/01/01"[Date - Publication] : "2025/03/31"[Date - Publication]) Filters: Clinical Trial, Randomized Controlled Trial, Humans

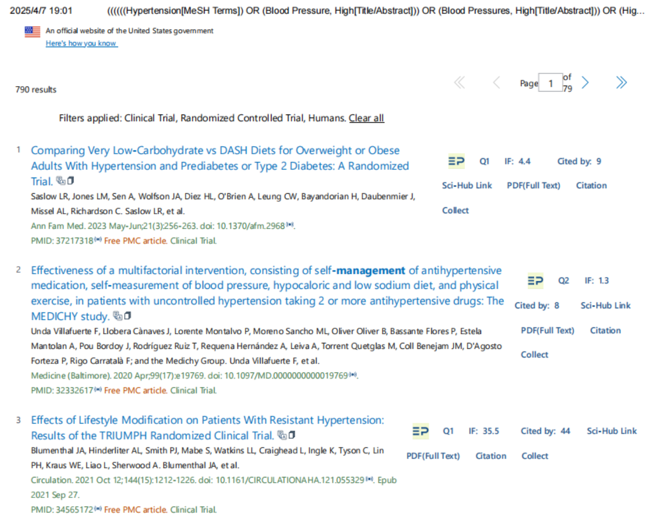
2025/4/7 19:01 ((((((Hypertension[MeSH Terms]) OR (Blood Pressure, High[Title/Abstract])) OR (Blood Pressures, High[Title/Abstract])) OR (Hig...

An official website of the United States government
Here's how you know

790 results

Page 1 of 79

Filters applied: Clinical Trial, Randomized Controlled Trial, Humans. Clear all

1 Comparing Very Low-Carbohydrate vs DASH Diets for Overweight or Obese Adults With Hypertension and Prediabetes or Type 2 Diabetes: A Randomized Trial.
Saslow LR, Jones LM, Sen A, Wolfson JA, Diez HL, O'Brien A, Leung CW, Bayandorian H, Daubenmier J, Missel AL, Richardson C. Saslow LR, et al.
Ann Fam Med. 2023 May-Jun;21(3):256-263. doi: 10.1370/afm.2968.
PMID: 37217318 Free PMC article. Clinical Trial.
Q1 IF: 4.4 Cited by: 9 Sci-Hub Link PDF(Full Text) Citation Collect

2 Effectiveness of a multifactorial intervention, consisting of self-**management** of antihypertensive medication, self-measurement of blood pressure, hypocaloric and low sodium diet, and physical exercise, in patients with uncontrolled hypertension taking 2 or more antihypertensive drugs: The MEDICHY study.
Unda Villafuerte F, Llobera Cànaves J, Lorente Montalvo P, Moreno Sancho ML, Oliver Oliver B, Bassante Flores P, Estela Mantolan A, Pou Bordoy J, Rodríguez Ruiz T, Requena Hernández A, Leiva A, Torrent Quetglas M, Coll Benejam JM, D'Agosto Forteza P, Rigo Carratalà F; and the Medichy Group. Unda Villafuerte F, et al.
Medicine (Baltimore). 2020 Apr;99(17):e19769. doi: 10.1097/MD.0000000000019769.
PMID: 32332617 Free PMC article. Clinical Trial.
Q2 IF: 1.3 Cited by: 8 Sci-Hub Link PDF(Full Text) Citation Collect

3 Effects of Lifestyle Modification on Patients With Resistant Hypertension: Results of the TRIUMPH Randomized Clinical Trial.
Blumenthal JA, Hinderliter AL, Smith PJ, Mabe S, Watkins LL, Craighead L, Ingle K, Tyson C, Lin PH, Kraus WE, Liao L, Sherwood A. Blumenthal JA, et al.
Circulation. 2021 Oct 12;144(15):1212-1226. doi: 10.1161/CIRCULATIONAHA.121.055329. Epub 2021 Sep 27.
PMID: 34565172 Free PMC article. Clinical Trial.
Q1 IF: 35.5 Cited by: 44 Sci-Hub Link PDF(Full Text) Citation Collect

(2) Cochrane Library (CINAHL 30, CT.gov 972, ICTRP 250, totally 1252, update to March 31st, 2025)

Search Name:

Date Run: 07/04/2025 17:34:02

#1 MeSH descriptor: [Hypertension] explode all trees 25159

#2 (Hypertension OR Blood Pressure, High OR High Blood Pressure OR High Blood Pressures OR Blood Pressures, High):ti,ab,kw (Word variations have been searched) 104200

#3 MeSH descriptor: [Disease Management] explode all trees 7507

#4 (Disease management OR Management, Disease OR Disease Managements OR Managements, Disease):ti,ab,kw (Word variations have been searched) 72499

#5 MeSH descriptor: [Randomized Controlled Trials as Topic] explode all trees 59432

#6 (Randomized Controlled Trials OR Trials, Randomized Clinical OR Clinical Trials, Randomized OR Controlled Clinical Trials, Randomized OR Random*):ti,ab,kw (Word variations have been searched) 1380317

#7 MeSH descriptor: [Prospective Studies] explode all trees 126165

#8 (Prospective Studies OR Studies, Prospective OR Prospective Study OR Study, Prospective):ti,ab,kw (Word variations have been searched) 271873

#9 #1 OR #2 104200

#10 #3 OR #4 77438

#11 #5 OR #6 OR #7 OR #8 1426656

#12 #9 AND #10 AND #11 5119 5669

(Publication date: inception to 31/03/2025; Trials: 5529)

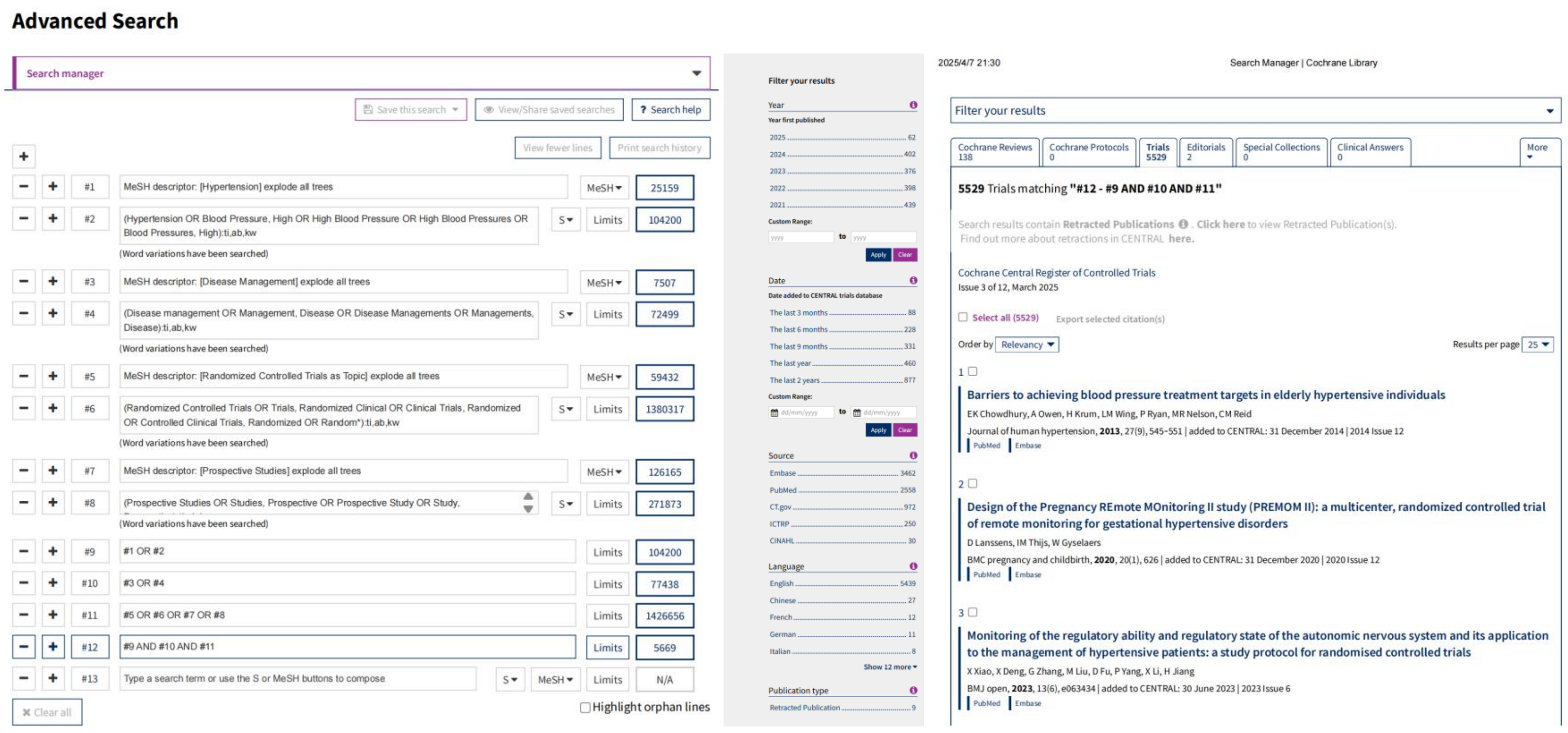

(3) EMBASE

Embase (totally 8041, update to March 31st, 2025)

#13. #12 AND ('clinical article'/de OR 'clinical trial'/de OR 'clinical trial topic'/de OR 'cohort analysis'/de OR 'comparative effectiveness'/de OR 'comparative study'/de OR 'controlled clinical trial'/de OR 'controlled study'/de OR 'double blind procedure'/de OR 'evidence based medicine'/de OR 'human'/de OR 'intervention study'/de OR 'longitudinal study'/de OR 'major clinical study'/de OR 'multicenter study'/de OR 'multicenter study topic'/de OR 'observational study'/de OR 'pilot study'/de OR 'prospective study'/de OR 'randomized controlled trial'/de OR 'randomized controlled trial topic'/de) AND 'article'/it AND [article]/lim AND [humans]/lim AND [clinical study]/lim AND [01-01-1966]/sd NOT [01-04-2025]/sd AND [<1966-2025]/py 8041

#12. #9 AND #10 AND #11 13888

#11. #5 OR #6 OR #7 OR #8 3275635

#10. #3 OR #4 854392

#9. #1 OR #2 1495478

#8. ((prospective AND method:ti,ab OR prospective) AND stud*:ti,ab OR study,) AND prospective:ti,ab 1179056

#7. prospective AND 'study'/exp 154

#6. (randomized AND controlled AND trial:ti,ab OR randomized) AND controlled AND trials:ti,ab OR random*:ti,ab 2223356

#5. randomized AND controlled AND trial AND topic 303495

#4. ((((((disease AND management:ti,ab OR diseases) AND management:ti,ab OR disorder) AND management:ti,ab OR disorders) AND management:ti,ab OR management) AND of AND disease:ti,ab OR management) AND of AND disorder:ti,ab OR illness) AND management:ti,ab 140374

#3. disease AND 'management'/exp 725564

#2. (((((hypertension:ti,ab OR high) AND ('blood'/exp OR blood) AND pressure:ti,ab OR htn) AND hypertension:ti,ab OR hypertensive) AND disease:ti,ab OR hypertensive) AND effect:ti,ab OR hypertensive) AND response:ti,ab 27418

#1. 'hypertension'/exp OR hypertension 1489207

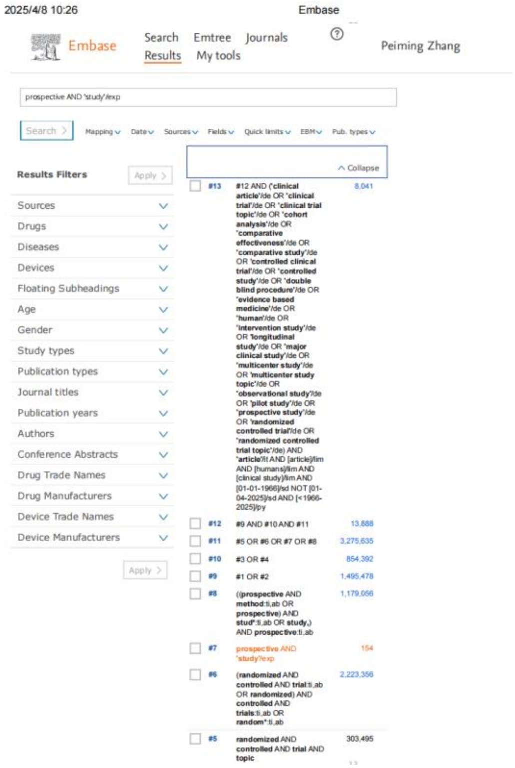

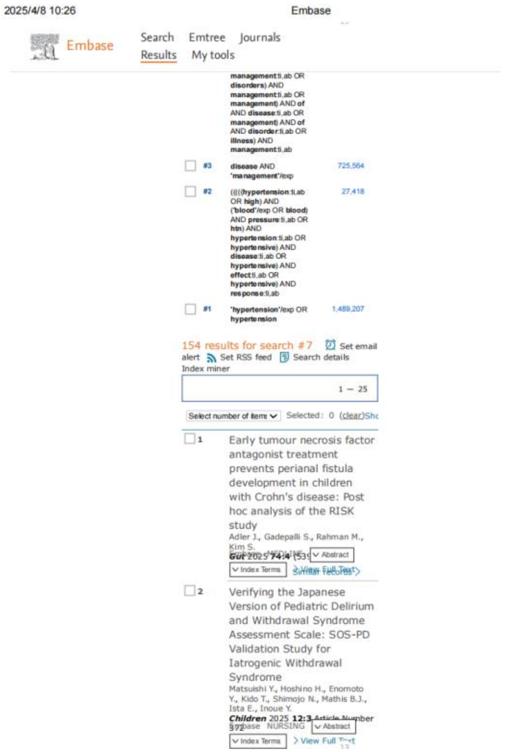

2 Identification and Classification of Chronic Disease Management

According to a systematic review by Zwar N. et al. (THE UNIVERSITY OF NEW SOUTH WALES SCHOOL OF PUBLIC HEALTH AND COMMUNITY MEDICINE, AUSTRALIAN PRIMARY HEALTHCARE RESEARCH INSTITUTE) in 2006[1], the chronic disease management (CDM)/Chronic Care Model (CCM) was developed in the USA after an extensive review of the literature and is the most widely known model of care for people with chronic conditions[2]. The model describes the elements essential for improvements in the care of people with chronic conditions, operating within the context of the triangle of the community, the health care system and the provider organisation[3]. The six elements of the CCM are[2][3]:

Delivery System Design (DSD) The structure of the medical practice to create teams with a clear division of labour and separating the acute from the planned care. Planned visits and follow up are important features.

Self Management Support (SMS) Collaboratively helping patients and their families to acquire the skills and confidence to manage their condition. Provide self management tools, referrals to community resources, routinely assessing progress.

Decision Support (DS) Integration of evidence based clinical guidelines into practice and reminder systems. Guidelines reinforced by clinical "champions" providing education to other health professionals.

Clinical Information Systems (CIS) Three important roles of computer information systems: Reminder system to improve compliance with guidelines, feedback on performance measures and registries for planning the care for CD.

Community Resources (CR) Linkages with hospitals providing patient education classes or home care agencies to provide case managers. Linkages with community based resources – exercise programs, self help groups, and senior centres

Health Care Organisation (HCO) The structure, goals and values of the provider organisation. Its relationship with purchaser, insurers and other providers underpins the model.

According to the review by Zwar N. et al.[1], CDM/CCM includes one or certain number of the operational forms respectively as follows:

(1) DS

Implementation of evidence-based guidelines

Educational meetings with professionals

Distributional of educational materials among professionals

(2) SMS

Patient educational sessions

Patient motivational counselling

Distribution of educational materials

(3) DSD

Multidisciplinary teams

(4) SMS+DSD

Multidisciplinary teams+Patient educational sessions

Multidisciplinary teams+Patient motivational counselling

(5) CIS

Audit and feedback

(6) HCO

Little published experimental evidence

(7) CR

Little published experimental evidence

Reference

[1] Zwar N, Harris M, Griffiths R, et al. A systematic review of chronic disease management[J]. 2006.

[2] Wagner E H, Austin B T, Von Korff M. Organizing care for patients with chronic illness[J]. The Milbank Quarterly, 1996: 511-544.

[3] Bodenheimer T, Wagner E H, Grumbach K. Improving primary care for patients with chronic illness[J]. Jama, 2002, 288(14): 1775-1779.

3 Included Literature

STable 1. Characteristics of the Included Studies

No.	Study	Inclusion Criteria	Age	Gender	Country/ Region	Specific Area	Management Mode (Sample Size)	Outcomes
1	Anderegg, M. D. 2018*	(1)SH (SBP/DBP≥140/90mmHg) (2)Hypertension (SBP/DBP≥130/80mmHg) with diabetes/ chronic kidney disease	62.15(11.80)	Male: 121(36.1%) Female: 214(63.9%)	USA	Community hospital	SMS+DS+CIS (227) vs None(108)	(1)SBP change (2)DBP change (3)BP control rate (SBP/DBP<140/90mmHg, or SBP/DBP<130/80mmHg with diabetes/ chronic kidney disease)
2	Aekplakorn W. 2016#	Hypertension (SBP/DBP≥140/90mmHg)	59.43(9.29)	Male: 78(34.8%) Female: 146(65.2%)	Thailand	Urban community hospital	SMS+CIS(111) vs SMS(113)	(1)SBP change (2)DBP change (3)BMI (4)BP out-of-control rate (SBP/DBP>140/90mmHg) (5)Adherence to Self-Monitoring Blood Pressure
3	Lee L L. 2007#	Hypertension (SBP=140to179mmHg)	71.3(6.05)	Male: 118(58.4%) Female: 84(41.6%)	Taiwan, China	Community	SMS+DSD(102) vs None(100)	(1)SBP change (2)Movement self-efficacy change

4	Li R. 2022#	Hypertension (SBP/DBP≥140/90mmHg) with type 2 diabetes	56.54(3.67)	Male: 43(63.2%) Female: 25(36.8%)	China	Outpatient and inpatient	SMS(34) vs None(34)	(1)Changes in knowledge about hypertension and diabetes (2)BP change (3)BS change (4)Changes in psychological change scales
5	Ling D. 2021#	Hypertension (SBP/DBP≥140/90mmHg)	35-50：14(4.67%) 50-65：13(45.33%) ≥65：150(50.00%)	Male: 210(70%) Female: 90(30%)	China	Outpatient and inpatient	SMS+DSD(162) vs None(138)	(1)Awareness rate of hypertension knowledge (2)SBP change (3)DBP change (4)BMI (5)WC (6)Psychological indicators (7)QoF
6	Ma Y. 2022#	Hypertension (SBP/DBP≥140/90mmHg) and taking medication	60.6±10.0	Male: 99(47.1%) Female: 111(52.9%)	China	Community	SMS+DSD(105) vs None(105)	(1)BP control rate (SBP/DBP＜140/90) (2)SBP change (3)DBP change (4)Weight change (5)WC change (6)BMI change (7)BMI normal rate change (8)Self-care effectiveness
7	Kuhmmer R. 2016#	Hypertension (SBP/DBP≥140/90mmHg)	59.5±10.50	Male: 75(29.3%)	Brazil	Hospital	SMS+DSD+CIS(128)	(1)BP control rate (SBP/DBP＜140/90)

				Female: 181(70.7%)			vs DSD(128)	(2)SBP change<br>(3)DBP change<br>(4)Medication compliance
8	Magid D. J. 2013#	Hypertension (SBP/DBP≥140/90mmHg)	59.55±11.09	Male: 210(60.3%)<br>Female: 138(39.7%)	USA	Community	SMS+DSD+CIS<br>Vs(175)<br>SMS(173)	(1)BP control rate (SBP/DBP＜140/90)<br>(2)SBP change<br>(3)DBP change<br>(4)Medication compliance
9	Manze M. G. 2015#	Hypertension (SBP/DBP≥140/90mmHg)	21-30：0.5<br>31-40：2.0<br>41-50：16.3<br>51-60：32.0<br>61-70：29.6<br>71-80：15.3<br>81-90: 4.4	Male: 89(27.6%)<br>Female: 233(72.4%)	USA	Community	SMS(119)<br>vs<br>None(84)	(1)Hypertension treatment positivity<br>(2)SBP change<br>(3)DBP change<br>(4)Medication compliance
10	Margolis K. L. 2018*	(1)SH (SBP/DBP≥140/90mmHg)<br>(2)Hypertension (SBP/DBP≥130/80mmHg) with diabetes/ chronic kidney disease	61.11±12.00	Male: 183(56.13%)<br>Female: 143(4.87%)	USA	Community	SMS+DSD(228)<br>vs<br>None(222)	(1)SBP change<br>(2)DBP change<br>(3)Drug compliance<br>(4)Self-efficacy change

								(5)Nursing satisfaction score<br>(6)The number of antihypertensive medication classes change<br>(7)The proportion of patients using a home BP monitor<br>(8)BP control mean score<br>(9)Add salt after served at table daily or more often rate
11	Marquez Contreras E. 2019*	Hypertension (Criteria ESH-ESC 2013)	57.39±9.49	Male: 71(48%)<br>Female: 77(52%)	Spain	Community	SMS+DSD(73)<br>vs<br>None(75)	(1)Follow-up dropout rate<br>(2)Blood pressure control rate (SBP/DBP＜140/90)<br>(3)Medication compliance
12	McManus R. J. 2010#	Hypertension (SBP/DBP≥140/90mmHg)	66.40±8.79	Male: 225(47%)<br>Female: 255(53%)	UK	Community	SMS+DSD(234)<br>vs<br>None(246)	(1)Preference for blood pressure monitoring method<br>(2)SBP change<br>(3)DBP change<br>(4)Drug side effects
13	Mendis S. 2010*	Hypertension (SBP/DBP≥140/90mmHg)	54.52(9.53)	Male: 1071(44.7%)<br>Female: 1326(55.3%)	China and Nigeria	Community	SMS+DS(1191)<br>vs<br>None(1206)	(1)SBP change<br>(2)DBP change<br>(3)Risk factors

14	Miao J. H. 2020#	Hypertension (SBP/DBP≥140/90mmHg)	67.4(7.8)	Male: 76(48.7%) Female: 80(51.3%)	China	Community	SMS(78) vs None(78)	(1)SBP change (2)DBP change (3)Self-care behavior score (4)Self-efficacy score (5)Patient satisfaction
15	Blumenthal J. A. 2021#	Resistant hypertension (defined as the use of 3 or more antihypertensive drugs for at least 6 weeks, clinical SBP/DBP ≥ 130/80 mmHg, or the need for 4 or more drugs to achieve SBP ≤ 130 mmHg and DBP ≤ 80 mmHg. or SBP ≥120 mmHg)	63 (9)	Male: 73(52%) Female: 67 (48%)	USA	Community	SMS+CIS(90) vs SMS(40)	(1)SBP change (2)DBP change (3)24-hour DBP (4)CVD biomarkers (5)Medication compliance (6)Aerobic fitness and functional ability changes (7)Biochemistry
16	Peiris D. 2019*	Hypertension (SBP/DBP>160/100mmHg)	60.6±10.79	Male: 3828(44.3%) Female: 4814(55.7%)	India	Rural community	DS+DSD+CIS (4348) vs SMS(4294)	(1)SBP change (2)DBP change (3)BMI change (4) QoF (5)BP control rate (SBP <140mmHg) (6)New CVD events (7)Use of antihypertensive drugs (8) Recent smoking (9) Health Enhancement Exercise

17	Perl S. 2016#	Hypertension (SBP/DBP＞140/90mmHg)	63.09(9.70)	Male: 160(62.5%) Female: 96(37.5%)	Austria	Community primary care center	SMS(137) vs None(119)	(1)SBP change<br>(2)DBP change<br>(3)BMI<br>(4)Weight<br>(5)BL profile<br>(6)BP control rate (<140/90 mmHg)<br>(7)CVD risk
18	Piette J. D. 2012#	Hypertension (non-diabetic SBP≥140mmHg, diabetic SBP≥130mmHg)	57.6±0.8	Male: 59(32.6%) Female: 122 (67.4%)	Honduras and Mexico	Community primary care center	SMS(89) vs None(92)	(1)SBP change<br>(2) Depression<br>(3) Drug problem count<br>(4)General health<br>(5)Satisfaction with BP control<br>(6)Time to discuss hypertension<br>(7)Satisfaction with the intervention
19	Zhou H. 2022*	Essential hypertension (SBP/DBP>140/90mmHg) or use of antihypertensive drugs	61.6(9.4)	Male: 1853(45.0%) Female: 2265 (55.0%)	China	Hospital	SMS(2985) vs None(1133)	(1) BP control rate (SBP/DBP ＜ 140/90 mm Hg, those with diabetes <130/80 mm Hg)<br>(2) SBP change<br>(3) DBP change

								(4)Changes in the number of people with various types of blood pressure<br>(5)Use rate of antihypertensive drugs<br>(6)Antihypertensive drug usage rate<br>(7)Lifestyle factors
20	Zhang, J. 2021#	Essential hypertension (SBP/DBP>140/90mmHg) or use of antihypertensive drugs	65-70：44(36.7%)<br>70-80：52(43.3%)<br>＞80：24(20.0%)	Male: 65(54.17%)<br>Female: 55 (45.83%)	China	Hospital	SMS+CIS(4243)<br>vs<br>Common(4268)	(1)Self-efficacy<br>(2)Self-management behavior<br>(3)Medication compliance<br>(4)QoL<br>(5)BP control rate (SBP/DBP <140/90mm Hg)<br>(6)SBP end value<br>(7)DBP end value
21	Zhang Y. 2017*	Hypertension (SBP/DBP>140/90mmHg)	Group1:64.5<br><br>Group2:65.5<br><br>Control:66.5 (mean/median)	Group1:<br>Male:48.1%<br>Female:51.9%<br>Group2:<br>Male:45.8%<br>Female:54.2%	China	Hospital	SMS+DS+DSD+CIS<br>vs<br>DS+DSD+CIS<br>vs<br>None<br><br>Total:1245	(1) Hospitalization rate<br>(2) Possibility of use in urban and rural hospitals<br>(3)Total hospitalization financial expenses<br>(4)QoL

				Control:<br>Male:44.1%<br>Female:55.9<br>%				
22	Whittle J. 2014*	Hypertension (non-diabetic SBP/DBP>140/90mmHg, or diabetic SBP/DBP>130/80mmHg), or use of at least one antihypertensive drug	68.16(10.15)	Male:<br>353(87.4%)<br>Female:<br>51 (12.6%)	USA	Community	SMS+DSD(21<br>9)<br>vs<br>SMS(185)	SBP change
23	Williams A. 2012#	Hypertension (SBP>130mmHg) with type 1 or 2 diabetes, and chronic kidney disease	67(9.62)	Male:<br>45(56.2%)<br>Female:<br>35 (43.8%)	Australia	Outpatient	SMS+DSD(39)<br>vs<br>None(41)	(1)Medication compliance<br>(2)Hematology indicators
24	Yoo H. J. 2009#	Hypertension (SBP/DBP>130/80mmHg), type 2 diabetes, BMI≥23.0kg/m2	58.17(8.76)	Male:<br>65(58.6%)<br>Female:<br>46 (41.4%)	South Korea	University hospital and community public health center	SMS+DSD+CI<br>S(57)<br>vs<br>None(54)	(1)Weight change<br>(2)BMI change<br>(3)WC change<br>(4)SBP change<br>(5)DBP change<br>(6)Arteriosclerosis<br>(7)Glycated hemoglobin HbA1c (%) change<br>(8)Rapid blood sugar change<br>(9)HOMA-IR change<br>(10)BL change

								(11)Change of adiponectin<br>(12)Changes of pro-inflammatory markers<br>(13)Exit rate<br>(14)Rate of actively sending information
25	Von Korff M. 2011#	Hypertension (SBP/DBP>140/90mmHg), accompanied by diabetes or coronary heart disease, or both, low-density lipoprotein concentration>3.37 mmol/L, or glycosylated hemoglobin 8.5% or higher, PHQ-9 depression score ≥ 10	56.8(11.32)	Not mentioned	USA	Community primary care center	SMS+DSD(106)<br>vs<br>None(108)	(1)Disability<br>(2)QoL
26	Ulm K. 2010#	Hypertension (office SBP>140mmHg)	65.46(8.69)	Male:<br>107(53.5%)<br>Female:<br>93 (46.5%)	Germany	Community hospital	SMS+DSD(102)<br>vs<br>None(98)	(1)SBP change<br>(2)DBP change<br>(3)Weight change<br>(4)BMI change<br>(5)Exercise amount change<br>(6)Smoking status<br>(7)Drinking quantity
27	Tonstad S. 2007#	Hypertension (SBP/DBP > 140/90mmHg measured in different scenarios at least two weeks apart, and mild hypertension (SBP 140-169mmHg and/or DBP 90-99mmHg) at study entry)	55(8.56)	Male:<br>36(73.5%)<br>Female:<br>13 (26.5%)	Norway	Urban Preventive Cardiology Specialist	SMS (31)<br>vs<br>None (18)	(1)smoking rate change<br>(2)SBP change<br>(3)DBP change<br>(4)Heart rate change<br>(5)BMI change<br>(6)Weight change<br>(7)WC change

								(8)HC change<br>(9)BL change<br>(10)BS change<br>(11)HbA1C%<br>(12)Total homocysteine<br>(13)Fibrinogen<br>(14)Cardiovascular Risk
28	Weltermann B. 2016*	Hypertension (office BP ≥140/90mmHg, ambulatory BP ≥130/80mmHg, or average home BP ≥135/80mmHg)	60.53(13.59)	Male: 58(56.3%)<br>Female: 45 (43.7%)	Germany	University general medicine practice network	SMS(63)<br>vs<br>None(40)	(1)BP control rate (ambulatory BP <130/80mm Hg)<br>(2)SBP change<br>(3)DBP change<br>(4)Changes in practical strategies<br>(5)Amount of antihypertensive drugs used
29	Shi W. 2022#	Hypertension (SBP/DBP≥140/90mmHg)	65.71±3.31	Male: 28(46.67%)<br>Female: 32 (53.33%)	China	Hospital	SMS+DSD+CIS(30)<br>Vs<br>SMS(30)	(1)Disease awareness rate<br>(2)Anxiety and depression scores<br>(3)Disease control ability<br>(4)QoL<br>(3)SBP change<br>(4)DBP change
30	Schwalm J. D. 2019*	Hypertension (SBP ≥ 160mmHg at 1 visit/ SBP 140-159mmHg at 1 visit, previously diagnosed with hypertension or taking	65.47±9.43	Male: 604(44.06%)	Colombia and Malaysia	Rural and urban	SMS+DS+DSD+CIS(644)<br>vs	(1) Cholesterol-adjustable risk score change<br>(2)Total cholesterol change

		antihypertensive medication; or SBP ≥ 130 mmHg at 1 visit, diagnosed with diabetes or taking medication to treat diabetes; or does not meet the above criteria but has a documented SBP of 140-159 mmHg on two separate visits, at least one day apart)		Female: 767 (55.94%)		communities	SMS(727)	(3) BL change<br>(4)BS change<br>(5)SBP change<br>(6)DBP change<br>(7)SBP control rate (SBP＜140mm Hg)<br>(8)BP control rate (SBP＜140mm Hg and DBP＜90mm Hg)<br>(9)INTERHEART Risk Score<br>(10)Rate of taking two or more antihypertensive drugs<br>(11)Rate of statin use<br>(12)Rates of co-administration of antihypertensive drugs and statins associated with free studies<br>(13)Medication compliance<br>(14)CVD risk<br>(15)Adverse event incidence
31	Rudd P. 2004#	Hypertension (SBP/DBP ≥ 140/90), and the patient must be treated with hypertension medication according to JNC VI standards.	59.51±9.49	Male: 70(46.67%)<br>Female:	USA	Medical health clinic	SMS+DSD+CIS(74)<br>Vs	(1)SBP change<br>(2)DBP change

				80 (53.33%)			None(76)	(3)Change of medication dosage<br>(4)Rate of reporting two or more antihypertensive drugs<br>(5)Rate of reporting no use of antihypertensive drugs<br>(6)Compliance
32	Rohla M. 2023*	Uncontrolled blood pressure: Individuals aged 18 years or older with HTN who have been pretreated with medication and have an automatically measured blood pressure AOBP exceeding the 135/85 mmHg threshold	67±11	Male: 221(44.5%)<br>Female: 276 (55.5%)	Austria	Community pharmacy	SMS+DSD+CIS(207)<br>Vs<br>SMS(290)	(1)BP control rate (SBP＜135mm Hg and DBP＜85mm Hg)<br>(2)Home BP control rate (SBP＜135mm Hg and DBP＜85mm Hg)<br>(3)SBP change<br>(4)DBP change
33	Rifkin D. E. 2013#	Hypertension (SBP/DBP>140/90mmHg), with stage 3 or greater chronic kidney disease; veteran	68.29±7.73	Male: 41(95.35%)<br>Female: 2 (4.65%)	USA	Specialty Clinic	SMS+DS+DSD+CIS(28)<br>vs<br>None(15)	(1)SBP change<br>(2)DBP change<br>(3)Mean arterial BP change<br>(4)Creatinine change<br>(5)Estimated glomerular filtration rate eGFR change<br>(6)Total medication dosage change<br>(7)Changes in the number of antihypertensive drugs taken

								(8)Medication compliance change
34	Qudah B. 2016#	Untreated hypertension (SBP/DBP>135/85 mmHg and not receiving any antihypertensive medication) or taking antihypertensive medication; receiving maintenance dialysis.	53.56±16.77	Male: 35(62.5%) Female: 21 (37.5%)	Jordan	Hospital	SMS+DS+DSD(29) vs None(27)	(1)SBP change before dialysis (2)SBP change after dialysis (3)DBP change before dialysis (4)DBP change after dialysis (5)Average SBP change during dialysis (6)Average DBP change during dialysis (7)Home SBP change (8)Home DBP change (9)Interdialytic weight gain rate (10)BP control rate (mean home blood pressure ≤135/85 mmHg) (11)Medication compliance
35	Pladevall M. 2010*	Essential hypertension (SBP/DBP≥140/90mmHg) , with the calculated 10-year cardiovascular disease risk 30% (World Health	43.34±5.83	Male: 46(58.23%) Female: 33 (41.77%)	Spain	Hospital	SMS+DS+CIS (40) Vs CIS(39)	(1)SBP change (2)DBP change (3)SBP out-of-control rate (≥140 mm Hg)

		Organization/International Society of Hypertension 1999 guidelines)						(4)DBP out-of-control rate (≥90 mm Hg)<br>(5)Cumulative main compliance<br>(6)Incidence rate of composite cardiovascular events
36	Bosworth H. B. 2011#	Hypertension (SBP/DBP>140/90mmHg), taking antihypertensive drugs; veteran	64±10	Male: 544(92%)<br>Female: 47 (8%)	USA	Community primary care center	Intensive SMS+DSD+CIS(147)<br>vs<br>SMS+CIS(148)<br>vs<br>SMS+DSD+CIS(149)<br>vs<br>None(147)	(1)SBP change<br>(2)DBP change<br>(3)BP control rate (SBP <140mmHg and DBP <90mmHg without diabetes, or SBP <130 and DBP <80mmHg with diabetes)
37	Meurer W. J. 2019#	Essential hypertension (SBP/DBP ≥ 160/100mmHg), taking antihypertensive drugs	49.49±12.41	Male: 31(56.36%)<br>Female: 24 (43.64%)	USA	University health system	SMS+DS+CIS (28)<br>Vs<br>None(27)	SBP change
38	Nolan R. P. 2012#	Grade 1 and 2 hypertension (SBP/DBP=140-159/90/99mmHg, or 16--180/100-110mmHg)	56.5±1.33	Male: 158(40.83%)	Canada	Family medicine outpatient	SMS(160)<br>vs<br>None(227)	(1)SBP change<br>(2)DBP change<br>(3)Pulse pressure change

				Female: 229 (59.17%)		clinics, tertiary care hospitals, university neurovascular laboratories (urban areas) and a public health unit (rural areas)		(4)Total cholesterol change<br>(5) Shedding rate
39	Nolan R. P. 2018#	Grade 1 and 2 hypertension (SBP/DBP=140-159/90/99mmHg, or 16--180/100-110mmHg); among participants not taking prescribed medications, hypertension was confirmed by family physicians and clinically assessed as SBP/DBP ≥140/90 mmHg; participants taking antihypertensive medications were required to have a baseline SBP/DBP ≥130/85mmHg.	57.63±1.83	Male: 109(41.44%)<br>Female: 154 (58.56%)	Canada	Roughly the same as above	SMS(132) vs None(131)	(1)SBP change<br>(2)DBP change<br>(3)PP change<br>(4)BL change<br>(5)Drug usage rate<br>(6)Change in cardiovascular risk
40	Varleta P. 2017#	Hypertension (SBP/DBP≥140/90mmHg); first-time prescription antihypertensive medication within the past 1 to 6 months	60.32±10.54	Male: 111(35.35%)<br>Female:203	Chile	Community	SMS(163) vs None(151)	Compliance

				(64.65%)				
41	Bove A. A. 2013#	Hypertension (SBP≥140mmHg)	59.59±13.59	Male: 84(34.85%) Female:157 (65.15%)	USA	University medical center and healthcare center	SMS+DS+CIS (120) vs None(121)	(1)BP control rate (SBP＜140mmHg)<br>(2)SBP change<br>(3)DBP change<br>(4)Rapid BS change<br>(5)BL change<br>(6)BMI change<br>(7)Medication compliance
42	Marquez Contreras E. 2019*	Mild to moderate hypertension (based on ESH-ESC 2013), i.e. ≥140/90mmHg, treated with antihypertensive medication for at least 1 year	57.39±9.49	Male: 71(47.97%) Female:77 (52.03%)	Spain	Community primary care center	SMS+DS+CIS (73) vs None(75)	(1)Compliance<br>(2)SBP change<br>(3)DBP change
43	McManus R. J. 2018#	Uncontrolled hypertension (SBP/DBP ≥ 140/90mmHg), and taking no more than 3 antihypertensive drugs	66.93±9.43	Male: 628(53.54%) Female: 545(46.46%)	UK	Community primary care center	SMS+DS+CIS (389) vs SMS(391) vs None(393)	(1)SBP change<br>(2)DBP change<br>(3)Total daily defined dose DDD<br>(4)DDD of thiazides and related diuretics<br>(5)DDD of β-blockers<br>(6)DDD for angiotensin-converting enzyme inhibitors and angiotensin II blockers

								(7)DDD of calcium antagonists<br>(8)Incidence of pain, joint stiffness, sleep disturbance, fatigue, cough, leg or ankle swelling, eye pain, dry mouth, numbness, and weakness<br>(9)Symptoms specific to high blood pressure<br>(10)Compliance
44	Morawski K. 2018#	Hypertension (SBP ≥ 140mmHg) and receiving at least 1 but no more than 3 first-line antihypertensive drugs treatment	52.04±10.3	Male: 164(39.9%)<br>Female: 247(60.1%)	USA	Community primary care center	SMS+DS+CIS (209)<br>vs<br>None(202)	(1)Medication compliance<br>(2)SBP change<br>(3)BP control rate (SBP/DBP≤140/90mmHg)
45	Bennett G. G. 2010#	High blood pressure (SBP/DBP>140/90mmHg), taking antihypertensive drugs	54.4±8.1	Male: 53(52.5%)<br>Female: 48(47.5%)	USA	Outpatient	SMS+DS+DSD+CIS(51)<br>vs<br>SMS(50)	(1)Weight change<br>(2)BMI<br>(3)WC<br>(4)SBP change<br>(5)DBP change
46	Bosworth H. B. 2005#	Hypertension (primary or secondary, without diabetes SBP/DBP>140/90mmHg, or with diabetes mellitus SBP/DBP >130/85mmHg) and a prescription for hypertension medications	63.5±11.36	Male: 576(97.96%)<br>Female: 12(2.04%)	USA	Community primary care center	SMS+DSD+CIS(294)<br>vs<br>None(294)	(1)BP control rate % (<140/90 mmHg non-diabetic, and <130/85 mmHg diabetic)<br>(2)Confidence in treatment

								(3)Knowledge about high blood pressure<br>(4)Self-reported compliance
47	Carter B. L. 2015(A)*	Uncontrolled hypertension (patients without diabetes SBP/DBP>140/90mmHg; with diabetes or CKD (SBP/DBP>130/80mmHg)	60.48±12.81	Male: 248(39.68%)<br>Female: 377(60.32%)	USA	Community primary care center	SMS+DS+DSD(401)<br>vs<br>None(224)	(1)BP control rate % (<140/90 mmHg non-diabetic, and <130/80mmHg with diabetes or CKD)<br>(2)Comorbidities<br>(3)Medication compliance<br>(4)Number of antihypertensive drugs<br>(5)SBP change<br>(6)DBP change
48	Carter B. L. 2015(B)#	Hypertension (≥140/90mmHg in non-diabetic patients or ≥130/80mmHg in diabetic patients); veteran	64.81±10.83	Male: 120(97.56%)<br>Female: 3(2.44%)	USA	Community outpatient clinic	SMS(61)<br>vs<br>None(62)	(1)BP control rate % (<140/90 mmHg non-diabetic, and <130/80mmHg diabetes)<br>(2)Use rate of thiazide drugs<br>(3)SBP change<br>(4)DBP change
49	Carter B. L. 2009*	Essential hypertension (without diabetes SBP/DBP=140-179/90-109mmHg, or with diabetes SBP/DBP=130-179/80-	58.29±14.06	Male: 165(41.04%)<br>Female:	USA	Community outpatient clinic	SMS+DS+DSD(192)<br>vs<br>None(210)	(1)BP control rate (<140/90 mmHg non-diabetic, and <130/80mmHg diabetes)

		109mmHg); and taking 0-3 antihypertensive medications)		237(58.96%)				(2)Average guideline compliance<br>(3)SBP change<br>(4)DBP change<br>(5)Number of antihypertensive drugs<br>(6)Medication compliance
50	Chang A. R. 2016*	Uncontrolled hypertension (patients with SBP/DBP ≥ 150/85mmHg, at least two outpatient BP measurements in the past 12 months, and planned serum creatinine within the next 30 days)	67.23±11.97	Male: 20(42.55%)<br>Female: 27(57.45%)	USA	Community health clinic	SMS+DS+DSD(24)<br>vs<br>None(23)	(1)BP control rate(<140/90 for non-proteinuric CKD and <130/80 for Proteinuric CKD)<br>(2)Impact of statin therapy<br>(3)Proportion of proteinuric CKD patients taking ACEI or ARB<br>(4)Proteinuria screening<br>(5)BL screening<br>(6)Urine albumin/creatinine ratio
51	Dean S. C. 2014#	Hypertension according to British Hypertension Society review criteria (BP≥150/90mmHg, or ≥140/80mmHg with cardiovascular, diabetes, or chronic kidney disease)	62±13.46	Male: 177(50.14%)<br>Female: 176(49.86%)	UK	Community polyclinic	SMS+DS(167)<br>vs<br>None(186)	(1)SBP change<br>(2)DBP change<br>(3)Percentage of patients with BP reaching target<br>(4)Participate in nurse-led clinic

								(5)Change in antihypertensive medication
52	Chen S. 2022*	Hypertension (SBP/DBP>140/90mmHg, accompanied by diabetes, coronary heart disease or kidney disease, SBP/DBP>130/80mmHg), taking antihypertensive drugs; with depression	74.46±8.23	Male: 789(33%) Female: 1576(67%)	China	Community primary care clinic	DSD+SMS+CIS+DS(1232) vs DS(1133)	(1)BP control rate % (<140/90 mmHg, and <130/80mmHg, with diabetes, coronary heart disease or kidney disease) (2)Daily living ability (3)Instrumental activities of daily living (4)Depression
53	Dejesus R. S. 2009#	Hypertension (SBP/DBP>140/90mmHg), with diabetes	Unknown:356(87%) Age≤60:13(3%) Age ＞ 60:41(10%)	Unknown:213(52%) Male: 92(23%) Female: 104(25%)	USA	Multi-specialty clinic	SMS(Education, 24) vs SMS(Education+ Self-monitoring, 30) vs None(356)	(1)BP control rate (SBP/DBP≤130/80mmHg) (2)SBP change (3)DBP change (4)Number of visits from nurses and doctors
54	Denver E A. 2003#	Hypertension (SBP/DBP>140/80mmHg, type 2 diabetes)	60.25±11.84	Male: 76(63.33%) Female: 44(36.67%)	UK	Hospital	SMS+CIS+DS (60) vs None(60)	(1)DBP change (2)SBP change (3)BL change (4)Changes in absolute stroke and coronary heart disease risk

55	Fairall L. R. 2016*	Hypertension (SBP/DBP>140/90mmHg), accompanied by hypertension, diabetes, and chronic respiratory diseases or depression	52.01±13.73	Male: 1199(27.29 %) Female: 3194(72.71 %)	South Africa	Primary care clinic	DS+CIS(2166) vs None(2227)	(1)Increase in drug dosage or quantity or change in drug category (2)Cardiovascular disease risks and risk factors (3)Intensive treatment rate (4)BMI
56	Farahmand F. 2019#	Hypertension (SBP/DBP ≥ 140/90mmHg on at least two occasions, receiving antihypertensive medication for at least 1 year, and no cognitive impairment)	70.27±5.26	Male: 28(56%) Female: 22(44%)	Iran	Medical center	SMS+CIS+DS (28) vs None(28)	(1)DBP change (2)SBP change (3)Incidence of hypertension
57	Fretheim A. 2006*	Hypertension (SBP/DBP>140/90mmHg) with hypercholesterolemia	60.88±13.5	Male: 2901(46.95 %) Female: 3278(53.05 %)	Norway	Clinic	SMS+CIS+DS (3316) vs None(2863)	(1)Proportion of first-time prescriptions for hypertension (including prescriptions of thiazides) (2)Proportion of patients who assess cardiovascular risk before prescribing antihypertensive or cholesterol-lowering drugs (3)Proportion of patients receiving treatment for hypertension or hypercholesterolemia for 3 months or longer and

								achieving recommended treatment goals
58	Frias J. 2017*	Hypertension (SBP ≥140mm Hg)<br>HbA 1c (≥7%) and failure of antihypertensive (≥2 drugs) and oral diabetes treatment; with type 2 diabetes	58.81±2.17	Male: 54(49.54%)<br>Female: 55(50.46%)	USA	Outpatient primary care center	SMS+CIS+DS (40)<br>vs<br>SMS+CIS+DS (40)<br>vs<br>None(29)	(1)SBP change<br>(2)HbA 1c changes<br>(3)LDL-C in weeks 4 and 12<br>(4)BP control rate % (<140/90 mm Hg)<br>(5)Medication compliance<br>(6)Average daily step count and duration of physical activity and rest and medical decisions<br>(7)LDL-C change<br>(8)Changes in PAM scores
59	Friedman R. H. 1996#	Hypertension (SBP/DBP≥160/90mm Hg)	76.5 (no discrete trends reported)	Male: 62(23.22%)<br>Female: 205(76.78%)	USA	Community	CIS+SMS(133)<br>vs<br>None(134)	(1)Medication compliance<br>(2)SBP change<br>(3)DBP change<br>(4)Patient user satisfaction<br>(5)Doctors' perceived utility and cost-effectiveness
60	Garcia-Peña C. 2001#	Hypertension (SBP/DBP＞160/90mmHg)	70.57±7.02	Male: 259(36.07%)<br>Female: 459(63.93%)	Mexico	Family medicine center	SMS+DS(364)<br>vs<br>None(354)	(1)DBP change value<br>(2)SBP change value<br>(3)Weight loss<br>(4)Sodium excretion

61	Gong K. 2020#	Essential hypertension (SBP/DBP≥140/90mmHg)	58.73±7.47	Male: 241(54.4%)<br>Female: 202(45.6%)	China	Hospital	SMS+DS+CIS (218)<br>vs<br>SMS(225)	(1)DBP change<br>(2)SBP change<br>(3)BP control rate % (general patient: BP ＜ 140/90mmHg; age ≥65：BP ＜150/90mmHg； chronic kidney disease or diabetes：BP＜130/80mmHg)<br>(4)Medication compliance
62	Green B. B. 2008#	Hypertension (SBP=140-199mmHg, or DBP=90-109mmHg)	59.1±8.5	Male: 372(47.8%)<br>Female: 406(52.2%)	USA	Medical center	SMS+CIS(259)<br>vs<br>SMS+CIS+DSD(261)<br>vs<br>SMS(258)	(1)DBP change<br>(2)SBP change<br>(3)BP control rate % (<140/90 mmHg)<br>(4)Number of types of antihypertensive drugs<br>(5)Use of aspirin<br>(6)BMI<br>(7)Physical activity<br>(8) Health-related quality of life<br>(9)Satisfaction with health plan<br>(10)Utilization of health care services

63	Green B. B. 2014#	Hypertension (SBP/DBP>140/90mmHg) BMI>26, Fragrance CVD risk score between 10%-25%	56.9±7	Male: 59(58%) Female: 42(42%)	USA	Healthcare system	SMS+CIS+DSD (51) vs SMS(50)	(1)DBP change (2)SBP change (3)Weight change (4)CVD risk score (5)BP control rate % (<140/90 mmHg) (6)Weight loss ≥4kg (7)BL change (8)Hypertension medication use
64	He J. 2023*	Hypertension (SBP/DBP ≥ 140/90mmHg, for people at high risk of CVD or those taking antihypertensive drugs, SBP/DBP ≥ 130/80mmHg)	63±9.2	Male: 13170(38.7 %) Female: 20825(61.3 %)	China	Village	SMS+DS+CIS (17407) vs DS(16588)	(1)DBP change value (2)SBP change value (3)Incidence of CVD (4)Mortality due to cardiovascular causes
65	Hotu C. 2010#	Hypertension (BP >130/80mmHg, diabetic nephropathy (>0.5 g proteinuria/24-h and serum creatinine 130-300 μmol/l)	61.5±6.96	Male: 35(53.85%) Female: 30(46.15%)	USA	Outpatient	SMS+DSD(33) vs None(32)	(1)DBP change (2)SBP change (3)24-hour urinary protein excretion (4)HbA1 c change (5)Change value of total cholesterol

								(6)Changes in echocardiographic parameters
66	Hunt J. S. 2008#	Highly uncontrolled BP (last SBP/DBP ≥ 160/100mmHg) or mildly uncontrolled BP (140-159/90-99 mmHg)	68±12.5	Male: 164(35.42%) Female: 299(64.58%)	USA	Primary care research network	SMS+DSD+DS(230) vs None(233)	(1)DBP change (2)SBP change (3)BP control rate (<140/90 mmHg) (4)Medication compliance (5)SF-36 investigation and evaluation (6)Satisfaction score
67	Jackson G. L. 2012#	Hypertension (SBP/DBP>140/90mmHg); with diabetes	63.5±10.3	Male: 626(91.8%) Female: 47(8.2%)	USA	Primary care clinic	SMS+DS+CIS (439) vs None(134)	(1)DBP change (2)SBP change (3)BP control rate % (<140/90 mmHg, or <130/80mmHg with diabetes)
68	Jafar T. H. 2022*	Hypertension (SBP/DBP>140/90mmHg)	64.5±9.8	Male: 462(50.4%) Female: 454(49.6%)	Singapore	Government clinic	SMS+DS+DSD(447) vs None(469)	(1)DBP change (2)SBP change (3)BP control rate (<140/90 mmHg)

								(4)CVD risk<br>(5)Urine ACR, average value<br>(6)Number of antihypertensive drugs per day
69	Woollard J. 2003#	Hypertension (SBP/DBP>140/90mmHg) or being treated with antihypertensive drugs; accompanied by non-insulin-dependent diabetes mellitus or coronary heart disease	60.25±2	Male: 107(50.71%)<br>Female: 104(49.29%)	Australia	General clinic	SMS+DS(143) vs None(68)	(1)BL change<br>(2)BMI<br>(3)Weight<br>(4)WC/HC<br>(5)Diet variables
70	Junling G. 2015#	Hypertension (SBP/DBP>140/90mmHg)	66.5±9.8	Male: 499(41.4%)<br>Female: 705(58.6%)	China	Community health center	SMS+DS+DSD(600) vs None(604)	(1)DBP change<br>(2)SBP change<br>(3)BMI<br>(4)Medication compliance<br>(5)Physical activity and diet<br>(6)Self-efficacy<br>(7)Social support score<br>(8)Coping skills score<br>(9)Belief score<br>(10)Doctor-patient communication<br>(11)Depression and health distress

71	Kario K. 2021#	Essential hypertension (office SBP 140-179 mmHg and/or DBP 90-109 mmHg); antihypertensive medication - >30 days after initial use or prescription of antihypertensive medication	56.8±9.2	Male: 98(67.12%)<br>Female: 48(32.88%)	Japan	Clinic	SMS+CIS(73)<br>vs<br>SMS(73)	(1)DBP change<br>(2)SBP change<br>(3)Weight change<br>(4)BMI change<br>(5)Pulse pressure change<br>(6)WC change
72	Khan S. A. 2023#	Hypertension (SBP/DBP≥140/90mmHg)	51.9±14.6	Male: 151(62.92%)<br>Female: 89(37.08%)	Pakistan	Hospital	SMS+CIS(120)<br>vs<br>None(120)	(1)DBP change<br>(2)SBP change<br>(3)Hypertension control value (① BP <140/90 mmHg; ② BP <130/80 mmHg; ③ BP <120/80 mmHg)
73	Khani Jeihooni A. 2023#	Hypertension (SBP/DBP≥140/90mmHg) with type 2 diabetes	Intervention group: 52.36 Control group 54.11 (no discrete trends reported)	Male: 135(45%)<br>Female: 165(55%)	Iran	Diabetes center	SMS+DSD(150)<br>vs<br>None(150)	(1)DBP change<br>(2)SBP change<br>(3)PRECEDE model structure score<br>(4)Lifestyle score<br>(5)Self-management score
74	Burla M. J. 2014#	Subclinical hypertensive heart disease (SBP/DBP >140/90mmHg for the first time and repeated measurements within 1 hour)	49.44±8.16	Male: 43(34.96%)<br>Female: 80(65.04%)	USA	Hospital emergency department	SMS+DSD(58)<br>vs<br>None(65)	(1)BP control rate % (control: BP <140/90 mmHg/diabetes or chronic kidney disease was present < 130/80 mmHg

								Intervention: BP<120/80 mmHg)<br>(2)QoL

#RCT: randomized controlled trial; *CRT: cluster randomized trial; SH: simple hypertension; SBP: systolic blood pressure; DBP: diastolic blood pressure; AOBP: automated office blood pressure; PP: pulse pressure; BS: blood sugar; BL: blood lipid; BMI: body mass index; WC: waist circumference; HC: hip circumference; LDL-C: low-density lipoprotein cholesterol; QoF: quality of life; CVD: cardiovascular disease; JNC: Joint National Committee; PAM：Patient Activation Measure (a validated measure of patient activation that includes person’s beliefs, motivation, and actions for self-care at these visits); ACR: albumin to creatinine ratio; PRECEDE: Predisposing, Reinforcing, Enabling Constructs in Educational, Diagnosis, and Evaluation; SMS: self-management support; DSD: delivery system design; DS: decision support; CIS: clinical information system.

Reference

[1]Anderegg M D, Gums T H, Uribe L, et al. Pharmacist intervention for blood pressure control in patients with diabetes and/or chronic kidney disease[J]. Pharmacotherapy: The Journal of Human Pharmacology and Drug Therapy, 2018, 38(3): 309-318.

[2]Aekplakorn W, Suriyawongpaisal P, Tansirisithikul R, et al. Effectiveness of self-monitoring blood pressure in primary care: a randomized controlled trial[J]. Journal of primary care & community health, 2016, 7(2): 58-64.

[3]Lee L L, Arthur A, Avis M. Evaluating a community-based walking intervention for hypertensive older people in Taiwan: a randomized controlled trial[J]. Preventive medicine, 2007, 44(2): 160-166.

[4]Li R, Xu W, Yang P, et al. The nursing effect of individualized management on patients with diabetes mellitus type 2 and hypertension[J]. Frontiers in Endocrinology, 2022, 13: 846419.

[5]Ling D, Wang R, Chen Q, et al. Assessment of chronic disease management mode (CDMM) on participants with primary hypertension[J]. Tropical Medicine & International Health, 2021, 26(7): 829-837.

[6]Ma Y, Cheng H Y, Sit J W H, et al. The effects of a smartphone-enhanced nurse-facilitated self-care intervention for Chinese hypertensive patients: A randomised controlled trial[J]. International Journal of Nursing Studies, 2022, 134: 104313.

[7]Kuhmmer R, Lazzaretti R K, Guterres C M, et al. Effectiveness of multidisciplinary intervention on blood pressure control in primary health care: a randomized clinical trial[J]. BMC health services research, 2016, 16: 1-13.

[8]Magid D J, Olson K L, Billups S J, et al. A pharmacist-led, American Heart Association Heart360 Web-enabled home blood pressure monitoring program[J]. Circulation: Cardiovascular Quality and Outcomes, 2013, 6(2): 157-163.

[9]Manze M G, Orner M B, Glickman M, et al. Brief provider communication skills training fails to impact patient hypertension outcomes[J]. Patient education and counseling, 2015, 98(2): 191-198.

[10]Margolis K L, Asche S E, Dehmer S P, et al. Long-term outcomes of the effects of home blood pressure telemonitoring and pharmacist management on blood pressure among adults with uncontrolled hypertension: follow-up of a cluster randomized clinical trial[J]. JAMA network open, 2018, 1(5): e181617-e181617.

[11]Marquez Contreras E, Marquez Rivero S, Rodriguez Garcia E, et al. Specific hypertension smartphone application to improve medication adherence in hypertension: a cluster-randomized trial[J]. Current Medical Research and Opinion, 2019, 35(1): 167-173.

[12]McManus R J, Mant J, Bray E P, et al. Telemonitoring and self-management in the control of hypertension (TASMINH2): a randomised controlled trial[J]. The Lancet, 2010, 376(9736): 163-172.

[13]Mendis S, Johnston S C, Fan W, et al. Cardiovascular risk management and its impact on hypertension control in primary care in low-resource settings: a

cluster-randomized trial[J]. Bulletin of the World Health Organization, 2010, 88(6): 412-419.

[14]Miao J H, Wang H S, Liu N. The evaluation of a nurse-led hypertension management model in an urban community healthcare: A randomized controlled trial[J]. Medicine, 2020, 99(27).

[15]Blumenthal J A, Hinderliter A L, Smith P J, et al. Effects of lifestyle modification on patients with resistant hypertension: results of the TRIUMPH randomized clinical trial[J]. Circulation, 2021, 144(15): 1212-1226.

[16]Peiris D, Praveen D, Mogulluru K, et al. SMARThealth India: a stepped-wedge, cluster randomised controlled trial of a community health worker managed mobile health intervention for people assessed at high cardiovascular disease risk in rural India[J]. PLoS One, 2019, 14(3): e0213708.

[17]Perl S, Niederl E, Kos C, et al. Randomized evaluation of the effectiveness of a structured educational program for patients with essential hypertension[J]. American journal of hypertension, 2016, 29(7): 866-872.

[18]Piette J D, Datwani H, Gaudioso S, et al. Hypertension management using mobile technology and home blood pressure monitoring: results of a randomized trial in two low/middle-income countries[J]. Telemedicine and e-Health, 2012, 18(8): 613-620.

[19]Zhou H, Wang X, Yang Y, et al. Effect of a Multicomponent Intervention Delivered on a Web-Based Platform on Hypertension Control: A Cluster Randomized Clinical Trial[J]. JAMA Network Open, 2022, 5(12): e2245439-e2245439.

[20]Zhang J, Guo L, Mao J, et al. The effects of nursing of Roy adaptation model on the elderly hypertensive: a randomised control study[J]. Annals of palliative medicine, 2021, 10(12): 12149-12158.

[21]Zhang Y, Tang W, Zhang Y, et al. Effects of integrated chronic care models on hypertension outcomes and spending: a multi-town clustered randomized trial in China[J]. BMC public health, 2017, 17: 1-11.

[22]Whittle J, Schapira M M, Fletcher K E, et al. A randomized trial of peer-delivered self-management support for hypertension[J]. American journal of

hypertension, 2014, 27(11): 1416-1423.

[23]Williams A, Manias E, Walker R, et al. A multifactorial intervention to improve blood pressure control in co- existing diabetes and kidney disease: a feasibility randomized controlled trial[J]. Journal of advanced nursing, 2012, 68(11): 2515-2525.

[24]Yoo H J, Park M S, Kim T N, et al. A ubiquitous chronic disease care system using cellular phones and the internet[J]. Diabetic Medicine, 2009, 26(6): 628-635.

[25]Von Korff M, Katon W J, Lin E H B, et al. Functional outcomes of multi-condition collaborative care and successful ageing: results of randomised trial[J]. Bmj, 2011, 343.

[26]Ulm K, Huntgeburth U, Gnahn H, et al. Effect of an intensive nurse-managed medical care programme on ambulatory blood pressure in hypertensive patients[J]. Archives of cardiovascular diseases, 2010, 103(3): 142-149.

[27]Tonstad S, Alm C S, Sandvik E. Effect of nurse counselling on metabolic risk factors in patients with mild hypertension: a randomised controlled trial[J]. European journal of cardiovascular nursing, 2007, 6(2): 160-164.

[28]Weltermann B, Kersting C, Viehmann A. Hypertension Management in Primary Care: A Cluster Randomized Trial of a Physician-focused Educational Intervention[J]. Deutsches Ärzteblatt International, 2016, 113(10): 167.

[29]Shi W, Cheng L, Li Y. Influence of “hospital-community-family” integrated management on blood pressure, quality of life, anxiety and depression in hypertensive patients[J]. Computational and Mathematical Methods in Medicine, 2022, 2022.

[30]Schwalm J D, McCready T, Lopez-Jaramillo P, et al. A community-based comprehensive intervention to reduce cardiovascular risk in hypertension (HOPE 4): a cluster-randomised controlled trial[J]. The Lancet, 2019, 394(10205): 1231-1242.

[31]Rudd P, Miller N H, Kaufman J, et al. Nurse management for hypertension: a systems approach[J]. American journal of hypertension, 2004, 17(10): 921-

927.

[32]Rohla M, Haberfeld H, Tscharre M, et al. Pharmacist interventions to improve blood pressure control in primary care: a cluster randomised trial[J]. International journal of clinical pharmacy, 2023, 45(1): 126-136.

[33]Rifkin D E, Abdelmalek J A, Miracle C M, et al. Linking clinic and home: a randomized, controlled clinical effectiveness trial of real-time, wireless blood pressure monitoring for older patients with kidney disease and hypertension[J]. Blood pressure monitoring, 2013, 18(1): 8.

[34]Qudah B, Albsoul-Younes A, Alawa E, et al. Role of clinical pharmacist in the management of blood pressure in dialysis patients[J]. International journal of clinical pharmacy, 2016, 38: 931-940.

[35]Pladevall M, Brotons C, Gabriel R, et al. Multicenter cluster-randomized trial of a multifactorial intervention to improve antihypertensive medication adherence and blood pressure control among patients at high cardiovascular risk (the COM99 study)[J]. Circulation, 2010, 122(12): 1183-1191.

[36]Bosworth H B, Powers B J, Olsen M K, et al. Home blood pressure management and improved blood pressure control: results from a randomized controlled trial[J]. Archives of internal medicine, 2011, 171(13): 1173-1180.

[37]Meurer W J, Dome M, Brown D, et al. Feasibility of emergency department–initiated, mobile health blood pressure intervention: an exploratory, randomized clinical trial[J]. Academic Emergency Medicine, 2019, 26(5): 517-527.

[38]Nolan R P, Liu S, Shoemaker J K, et al. Therapeutic benefit of internet-based lifestyle counselling for hypertension[J]. Canadian Journal of Cardiology, 2012, 28(3): 390-396.

[39]Nolan R P, Feldman R, Dawes M, et al. Randomized controlled trial of e-counseling for hypertension: REACH[J]. Circulation: Cardiovascular Quality and Outcomes, 2018, 11(7): e004420.

[40]Varleta P, Acevedo M, Akel C, et al. Mobile phone text messaging improves antihypertensive drug adherence in the community[J]. The Journal of Clinical

Hypertension, 2017, 19(12): 1276-1284.

[41]Bove A A, Homko C J, Santamore W P, et al. Managing hypertension in urban underserved subjects using telemedicine—a clinical trial[J]. American heart journal, 2013, 165(4): 615-621.

[42]Marquez Contreras E, Marquez Rivero S, Rodriguez Garcia E, et al. Specific hypertension smartphone application to improve medication adherence in hypertension: a cluster-randomized trial[J]. Current Medical Research and Opinion, 2019, 35(1): 167-173.

[43]McManus R J, Mant J, Franssen M, et al. Efficacy of self-monitored blood pressure, with or without telemonitoring, for titration of antihypertensive medication (TASMINH4): an unmasked randomised controlled trial[J]. The Lancet, 2018, 391(10124): 949-959.

[44]Morawski K, Ghazinouri R, Krumme A, et al. Association of a smartphone application with medication adherence and blood pressure control: the MedISAFE-BP randomized clinical trial[J]. JAMA internal medicine, 2018, 178(6): 802-809.

[45]Bennett G G, Herring S J, Puleo E, et al. Web- based weight loss in primary care: a randomized controlled trial[J]. Obesity, 2010, 18(2): 308-313.

[46]Bosworth H B, Olsen M K, Gentry P, et al. Nurse administered telephone intervention for blood pressure control: a patient-tailored multifactorial intervention[J]. Patient education and counseling, 2005, 57(1): 5-14.

[47]Carter B L, Coffey C S, Ardery G, et al. Cluster-randomized trial of a physician/pharmacist collaborative model to improve blood pressure control[J]. Circulation: Cardiovascular Quality and Outcomes, 2015, 8(3): 235-243.

[48]Carter B L, Vander Weg M W, Parker C P, et al. Sustained blood pressure control following discontinuation of a pharmacist intervention for veterans[J]. The Journal of Clinical Hypertension, 2015, 17(9): 701-708.

[49]Carter B L, Ardery G, Dawson J D, et al. Physician and pharmacist collaboration to improve blood pressure control[J]. Archives of internal medicine, 2009, 169(21): 1996-2002.

[50]Chang A R, Evans M, Yule C, et al. Using pharmacists to improve risk stratification and management of stage 3A chronic kidney disease: a feasibility study[J]. BMC nephrology, 2016, 17(1): 1-9.

[51]Dean S C, Kerry S M, Khong T K, et al. Evaluation of a specialist nurse-led hypertension clinic with consultant backup in two inner city general practices: randomized controlled trial[J]. Family practice, 2014, 31(2): 172-179.

[52]Chen S, Conwell Y, Xue J, et al. Effectiveness of integrated care for older adults with depression and hypertension in rural China: A cluster randomized controlled trial[J]. PLoS Medicine, 2022, 19(10): e1004019.

[53]DeJesus R S, Chaudhry R, Leutink D J, et al. Effects of efforts to intensify management on blood pressure control among patients with type 2 diabetes mellitus and hypertension: a pilot study[J]. Vascular health and risk management, 2009: 705-711.

[54]Denver E A, Barnard M, Woolfson R G, et al. Management of uncontrolled hypertension in a nurse-led clinic compared with conventional care for patients with type 2 diabetes[J]. Diabetes care, 2003, 26(8): 2256-2260.

[55]Fairall L R, Folb N, Timmerman V, et al. Educational outreach with an integrated clinical tool for nurse-led non-communicable chronic disease management in primary care in South Africa: a pragmatic cluster randomised controlled trial[J]. PLoS medicine, 2016, 13(11): e1002178.

[56]Farahmand F, Khorasani P, Shahriari M. Effectiveness of a self-care education program on hypertension management in older adults discharged from cardiac-internal wards[J]. ARYA atherosclerosis, 2019, 15(2): 44.

[57]Fretheim A, Oxman A D, Håvelsrud K, et al. Rational prescribing in primary care (RaPP): a cluster randomized trial of a tailored intervention[J]. PLoS medicine, 2006, 3(6): e134.

[58]Frias J, Virdi N, Raja P, et al. Effectiveness of digital medicines to improve clinical outcomes in patients with uncontrolled hypertension and type 2 diabetes: prospective, open-label, cluster-randomized pilot clinical trial[J]. Journal of medical Internet research, 2017, 19(7): e246.

[59]Friedman R H, Kazis L E, Jette A, et al. A telecommunications system for monitoring and counseling patients with hypertension: impact on medication adherence and blood pressure control[J]. American journal of hypertension, 1996, 9(4): 285-292.

[60]Garcia-Peña C, Thorogood M, Armstrong B, et al. Pragmatic randomized trial of home visits by a nurse to elderly people with hypertension in Mexico[J]. International journal of epidemiology, 2001, 30(6): 1485-1491.

[61]Gong K, Yan Y L, Li Y, et al. Mobile health applications for the management of primary hypertension: A multicenter, randomized, controlled trial[J]. Medicine, 2020, 99(16): e19715.

[62]Green B B, Cook A J, Ralston J D, et al. Effectiveness of home blood pressure monitoring, Web communication, and pharmacist care on hypertension control: a randomized controlled trial[J]. Jama, 2008, 299(24): 2857-2867.

[63]Green B B, Anderson M L, Cook A J, et al. e-Care for heart wellness: a feasibility trial to decrease blood pressure and cardiovascular risk[J]. American journal of preventive medicine, 2014, 46(4): 368-377.

[64]He J, Ouyang N, Guo X, et al. Effectiveness of a non-physician community health-care provider-led intensive blood pressure intervention versus usual care on cardiovascular disease (CRHCP): an open-label, blinded-endpoint, cluster-randomised trial[J]. The Lancet, 2023, 401(10380): 928-938.

[65]Hotu C, Bagg W, Collins J, et al. A community-based model of care improves blood pressure control and delays progression of proteinuria, left ventricular hypertrophy and diastolic dysfunction in Māori and Pacific patients with type 2 diabetes and chronic kidney disease: a randomized controlled trial[J]. Nephrology Dialysis Transplantation, 2010, 25(10): 3260-3266.

[66]Hunt J S, Siemienczuk J, Pape G, et al. A randomized controlled trial of team-based care: impact of physician-pharmacist collaboration on uncontrolled hypertension[J]. Journal of general internal medicine, 2008, 23: 1966-1972.

[67]Jackson G L, Oddone E Z, Olsen M K, et al. Racial differences in the effect of a telephone-delivered hypertension disease management program[J]. Journal

of general internal medicine, 2012, 27: 1682-1689.

[68]Jafar T H, Tan N C, Shirore R M, et al. Integration of a multicomponent intervention for hypertension into primary healthcare services in Singapore—A cluster randomized controlled trial[J]. Plos Medicine, 2022, 19(6): e1004026.

[69]Woollard J, Burke V, Beilin L J, et al. Effects of a general practice-based intervention on diet, body mass index and blood lipids in patients at cardiovascular risk[J]. Journal of cardiovascular risk, 2003, 10(1): 31-40.

[70]Junling G, Yang L, Junming D, et al. Evaluation of group visits for Chinese hypertensives based on primary health care center[J]. Asia Pacific Journal of Public Health, 2015, 27(2): NP350-NP360.

[71]Kario K, Nomura A, Kato A, et al. Digital therapeutics for essential hypertension using a smartphone application: A randomized, open- label, multicenter pilot study[J]. The Journal of Clinical Hypertension, 2021, 23(5): 923-934.

[72]Khan S A, Hafeez A, Zaka A, et al. A Randomized Controlled Trial of Blood Pressure Reduction Based on Disease Control Priorities 3 in Pakistan to Manage and Control Hypertension[J]. High Blood Pressure & Cardiovascular Prevention, 2023, 30(4): 357-366.

[73]Khani Jeihooni A, Sobhani A, Afzali Harsini P, et al. Effect of educational intervention based on PRECEDE model on lifestyle modification, self-management behaviors, and hypertension in diabetic patients[J]. BMC endocrine disorders, 2023, 23(1): 6.

[74]Burla M J, Brody A M, Ference B A, et al. Blood pressure control and perceived health status in African Americans with subclinical hypertensive heart disease[J]. Journal of the American Society of Hypertension, 2014, 8(5): 321-329.

4 Supplementary Results of Meta Analysis

4.1 Forestplots

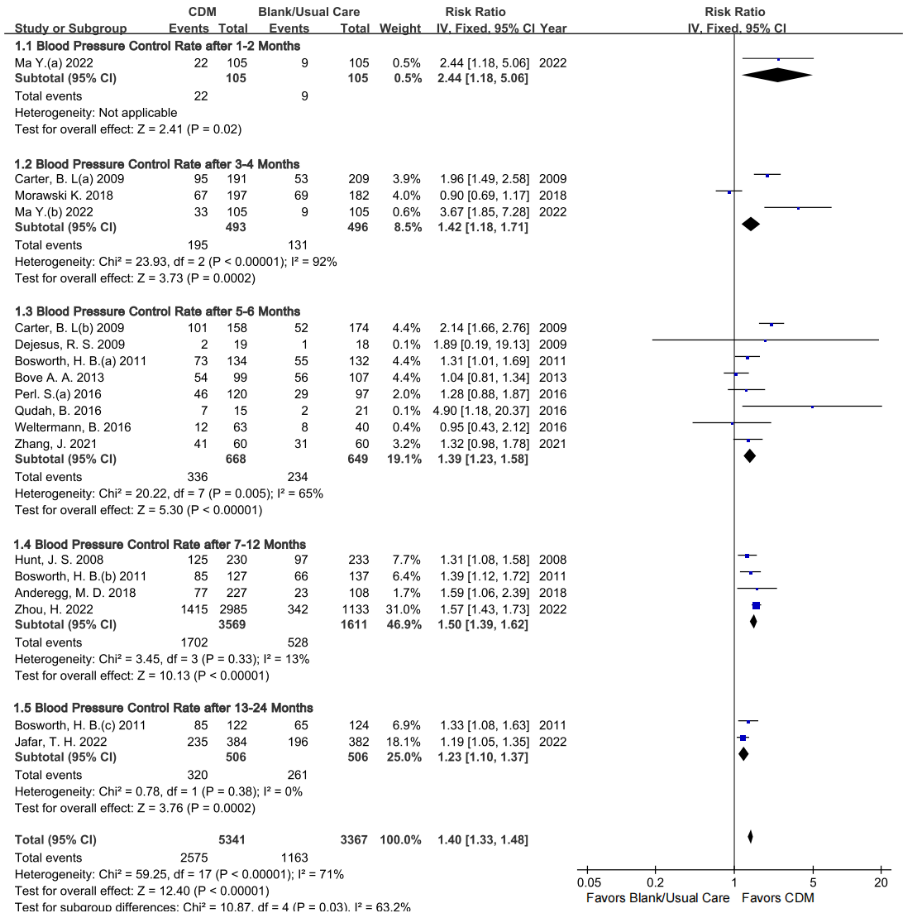

SFigure 1 Forestplot of the Blood Pressure Control Rate by Follow-up Time Point (IV, FEM)

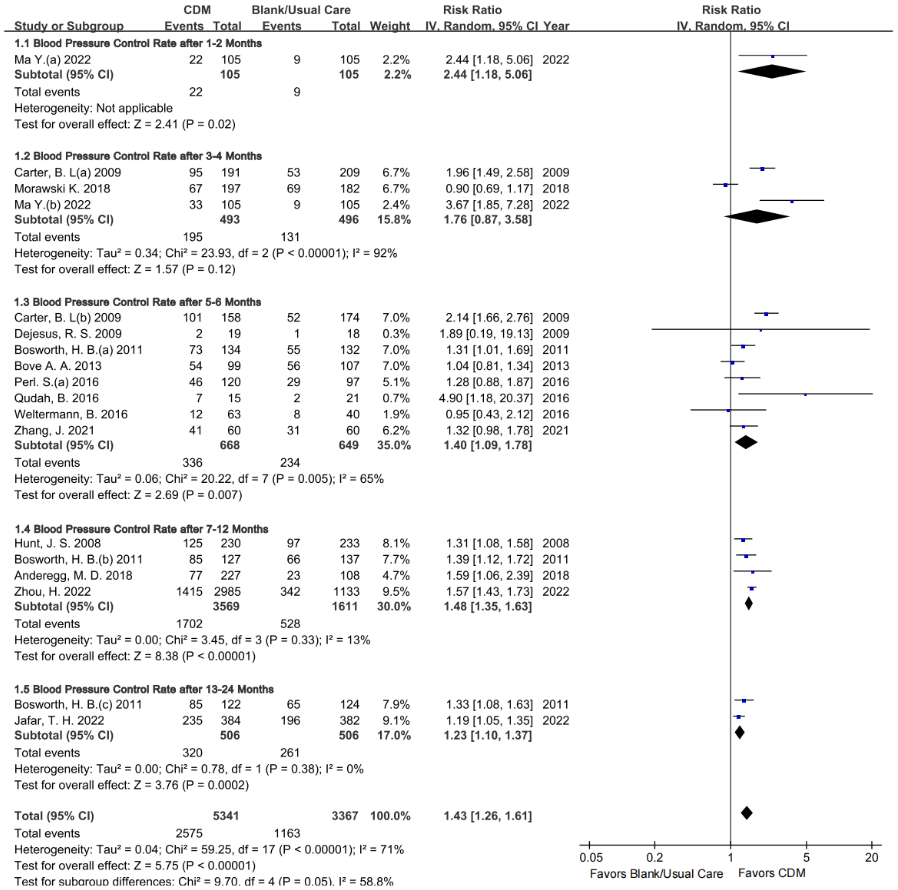

SFigure 2 Forestplot of the Blood Pressure Control Rate by Follow-up Time Point (IV, REM)

Study or Subgroup	CDM Events	CDM Total	Blank/Usual Care Events	Blank/Usual Care Total	Weight	Risk Ratio M-H, Random, 95% CI	Year
**1.1 Blood Pressure Control Rate after 1-2 Months**							
Ma Y.(a) 2022	22	105	9	105	2.2%	2.44 [1.18, 5.06]	2022
**Subtotal (95% CI)**		**105**		**105**	**2.2%**	**2.44 [1.18, 5.06]**	
Total events	22		9				
Heterogeneity: Not applicable							
Test for overall effect: Z = 2.41 (P = 0.02)							
**1.2 Blood Pressure Control Rate after 3-4 Months**							
Carter, B. L(a) 2009	95	191	53	209	6.7%	1.96 [1.49, 2.58]	2009
Morawski K. 2018	67	197	69	182	6.7%	0.90 [0.69, 1.17]	2018
Ma Y.(b) 2022	33	105	9	105	2.4%	3.67 [1.85, 7.28]	2022
**Subtotal (95% CI)**		**493**		**496**	**15.8%**	**1.76 [0.86, 3.60]**	
Total events	195		131				
Heterogeneity: Tau² = 0.35; Chi² = 24.24, df = 2 (P < 0.00001); I² = 92%							
Test for overall effect: Z = 1.56 (P = 0.12)							
**1.3 Blood Pressure Control Rate after 5-6 Months**							
Carter, B. L(b) 2009	101	158	52	174	7.0%	2.14 [1.66, 2.76]	2009
Dejesus, R. S. 2009	2	19	1	18	0.3%	1.89 [0.19, 19.13]	2009
Bosworth, H. B.(a) 2011	73	134	55	132	7.0%	1.31 [1.01, 1.69]	2011
Bove A. A. 2013	54	99	56	107	7.0%	1.04 [0.81, 1.34]	2013
Perl. S.(a) 2016	46	120	29	97	5.1%	1.28 [0.88, 1.87]	2016
Qudah, B. 2016	7	15	2	21	0.7%	4.90 [1.18, 20.37]	2016
Weltermann, B. 2016	12	63	8	40	1.9%	0.95 [0.43, 2.12]	2016
Zhang, J. 2021	41	60	31	60	6.3%	1.32 [0.98, 1.78]	2021
**Subtotal (95% CI)**		**668**		**649**	**35.1%**	**1.40 [1.09, 1.79]**	
Total events	336		234				
Heterogeneity: Tau² = 0.07; Chi² = 20.42, df = 7 (P = 0.005); I² = 66%							
Test for overall effect: Z = 2.67 (P = 0.007)							
**1.4 Blood Pressure Control Rate after 7-12 Months**							
Hunt, J. S. 2008	125	230	97	233	8.0%	1.31 [1.08, 1.58]	2008
Bosworth, H. B.(b) 2011	85	127	66	137	7.7%	1.39 [1.12, 1.72]	2011
Anderegg, M. D. 2018	77	227	23	108	4.8%	1.59 [1.06, 2.39]	2018
Zhou, H. 2022	1415	2985	342	1133	9.4%	1.57 [1.43, 1.73]	2022
**Subtotal (95% CI)**		**3569**		**1611**	**29.9%**	**1.48 [1.35, 1.63]**	
Total events	1702		528				
Heterogeneity: Tau² = 0.00; Chi² = 3.55, df = 3 (P = 0.31); I² = 15%							
Test for overall effect: Z = 8.13 (P < 0.00001)							
**1.5 Blood Pressure Control Rate after 13-24 Months**							
Bosworth, H. B.(c) 2011	85	122	65	124	7.8%	1.33 [1.08, 1.63]	2011
Jafar, T. H. 2022	235	384	196	382	9.1%	1.19 [1.05, 1.35]	2022
**Subtotal (95% CI)**		**506**		**506**	**16.9%**	**1.23 [1.10, 1.37]**	
Total events	320		261				
Heterogeneity: Tau² = 0.00; Chi² = 0.78, df = 1 (P = 0.38); I² = 0%							
Test for overall effect: Z = 3.76 (P = 0.0002)							
**Total (95% CI)**		**5341**		**3367**	**100.0%**	**1.43 [1.26, 1.61]**	
Total events	2575		1163				
Heterogeneity: Tau² = 0.04; Chi² = 60.61, df = 17 (P < 0.00001); I² = 72%							
Test for overall effect: Z = 5.70 (P < 0.00001)							
Test for subgroup differences: Chi² = 9.47, df = 4 (P = 0.05), I² = 57.8%							

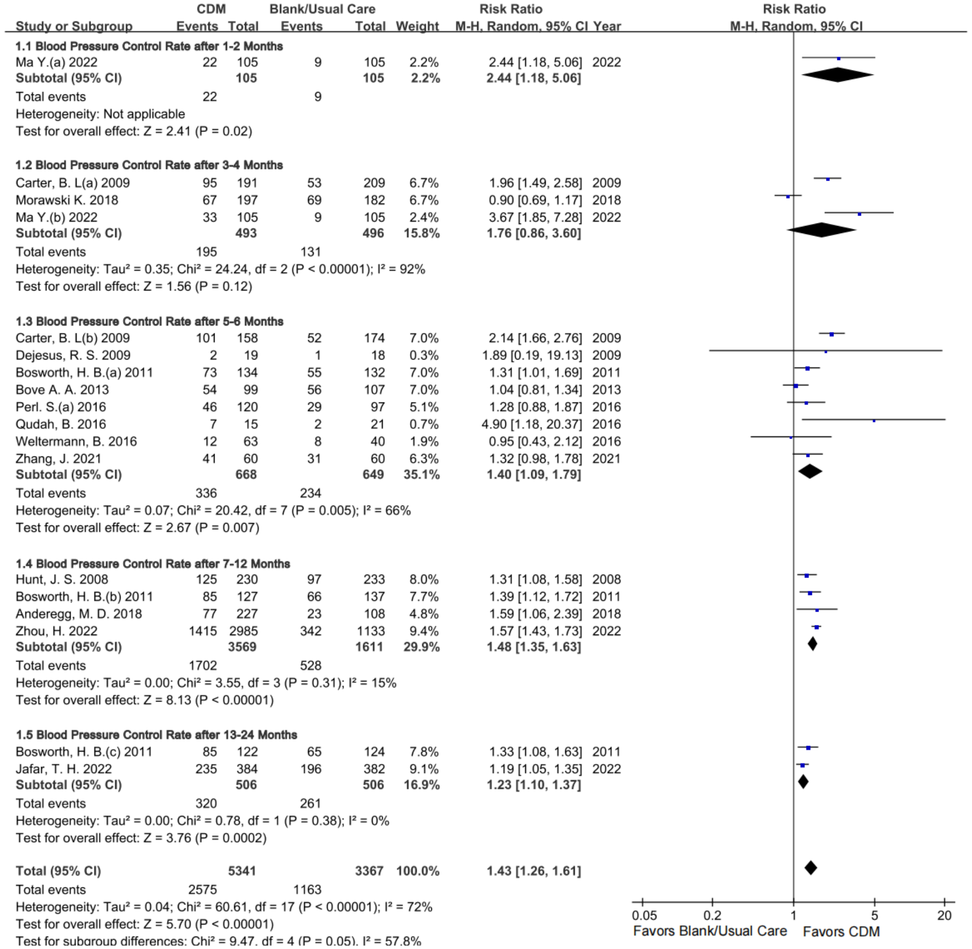

SFigure 3 Forestplot of the Blood Pressure Control Rate by Follow-up Time Point (MH, REM)

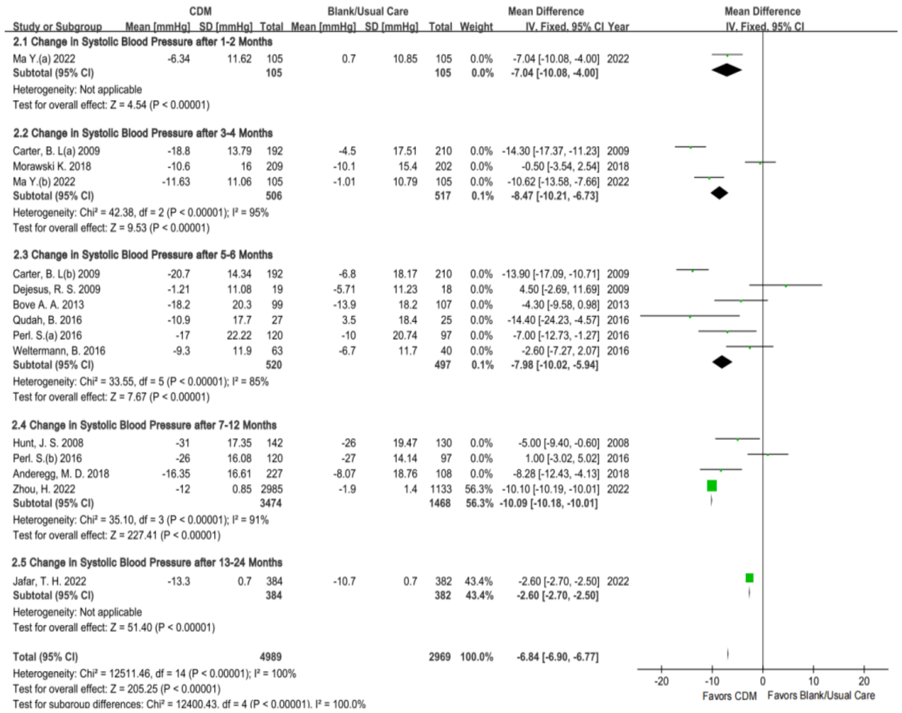

SFigure 4 Forestplot of the Change in SBP by Follow-up Time Point (IV, FEM)

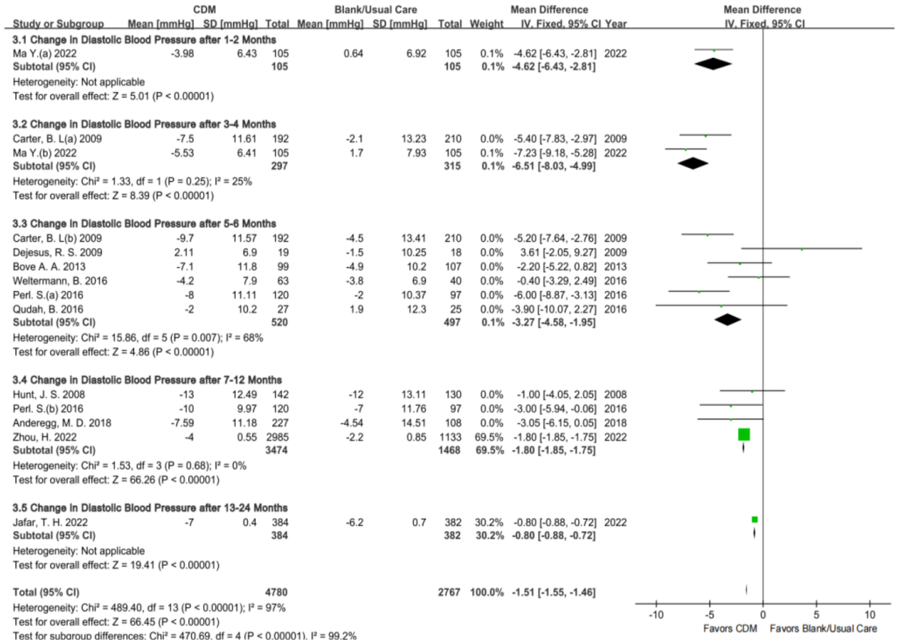

SFigure 5 Forestplot of the Change in DBP by Follow-up Time Point (IV, FEM)

*** Meta Regression for the Change in DBP with follow-up Time Points between 5 to 6 months (R 4.1.3)**

Mixed-Effects Model (k = 6; tau^2 estimator: REML) (**SFigure 6**)

tau^2 (estimated amount of residual heterogeneity): 2.8313 (SE = 4.3546)

tau (square root of estimated tau^2 value): 1.6826

I^2 (residual heterogeneity / unaccounted variability): 46.96%

H^2 (unaccounted variability / sampling variability): 1.89

R^2 (amount of heterogeneity accounted for): 57.90%

Test for Residual Heterogeneity:

QE(df = 4) = 7.9138, p-val = 0.0948

Test of Moderators (coefficient 2):

QM(df = 1) = 3.9962, p-val = 0.0456

Model Results:

	estimate	se	zval	pval	ci.lb	ci.ub	
intrcpt	0.4018	1.9386	0.2073	0.8358	-3.3978	4.2015	
Sample_Size	-0.0162	0.0081	-1.9991	0.0456	-0.0320	-0.0003	*

Signif. codes: 0 ‘***’ 0.001 ‘**’ 0.01 ‘*’ 0.05 ‘.’ 0.1 ‘ ’ 1

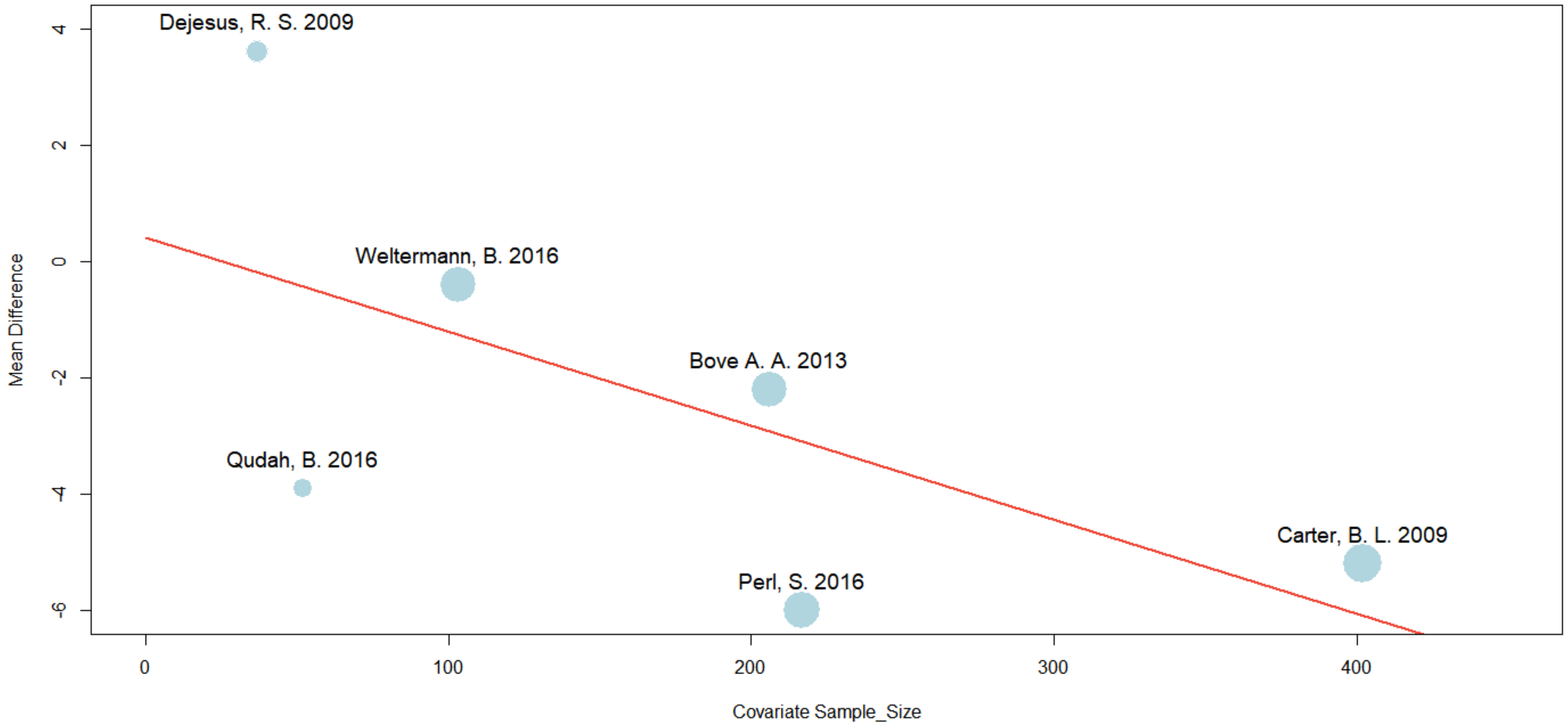

SFigure 6 Meta Regression for the Change in DBP with Follow-up Time Points between 5 to 6 months

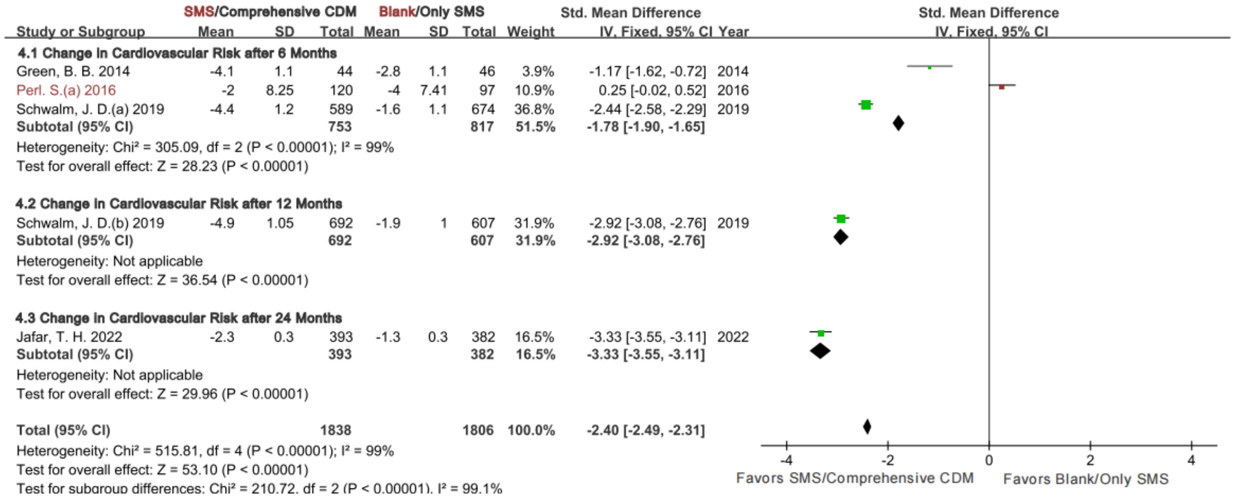

SFigure 7 Forestplot of the Change in Cardiovascular Risk by Follow-up Time Point (IV, FEM)

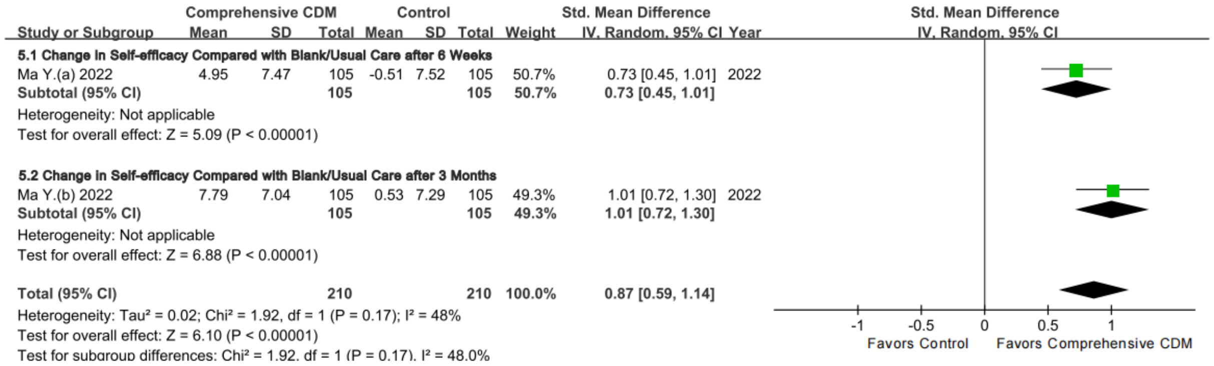

SFigure 8 Forestplot of the Change in Blood Pressure Control Self-efficacy by Follow-up Time Point (IV, REM)

4.2 Funnel Plots

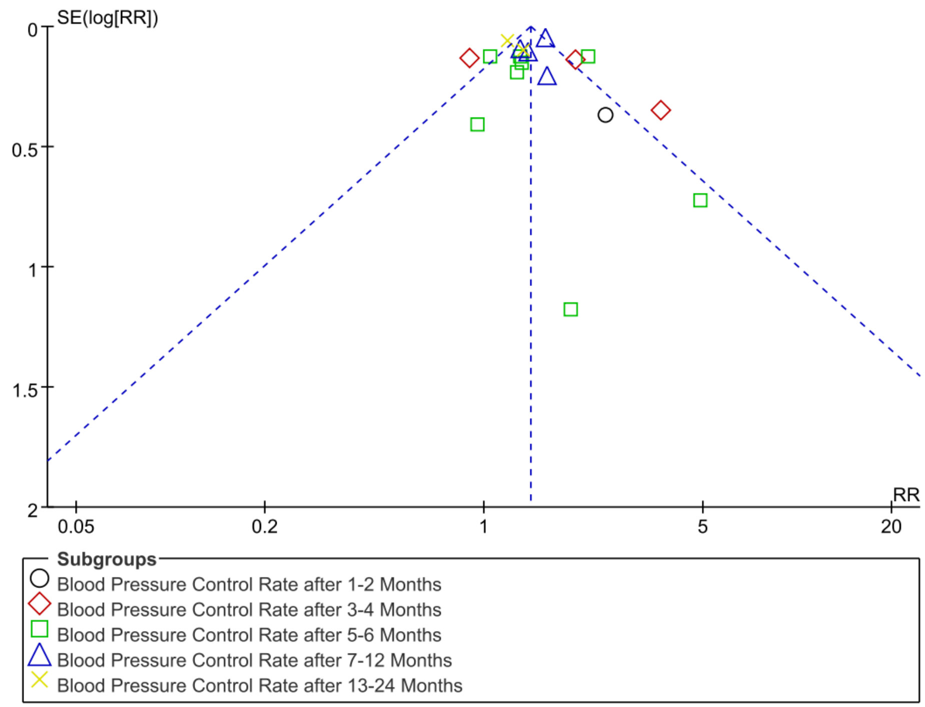

SFigure 9 Funnel Plot of the Blood Pressure Control Rate by Follow-up Time Point

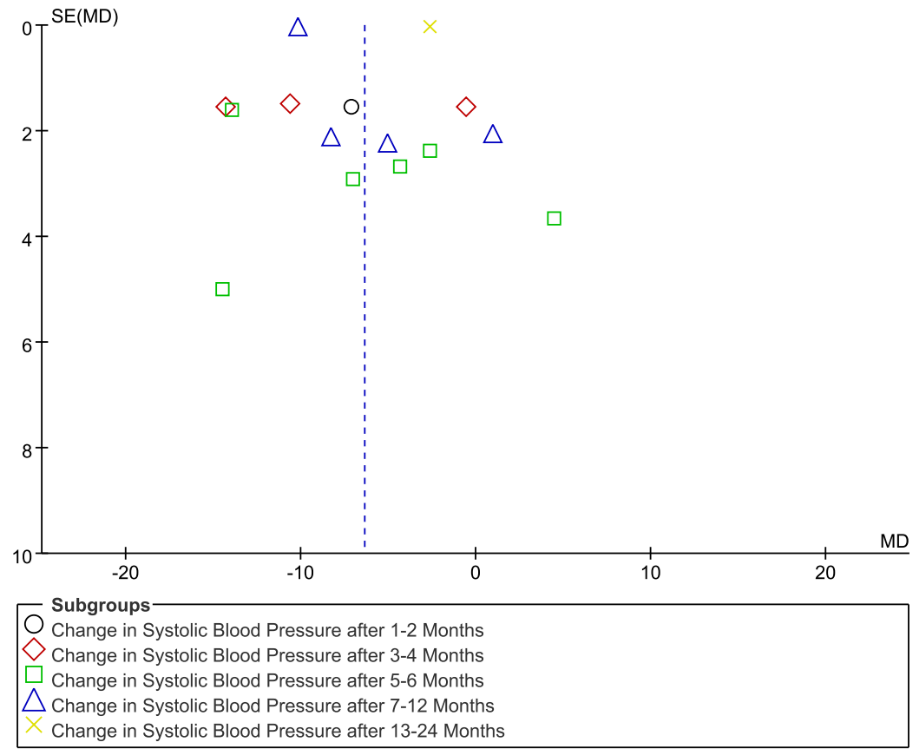

SFigure 10 Funnel Plot of the Change in SBP by Follow-up Time Point

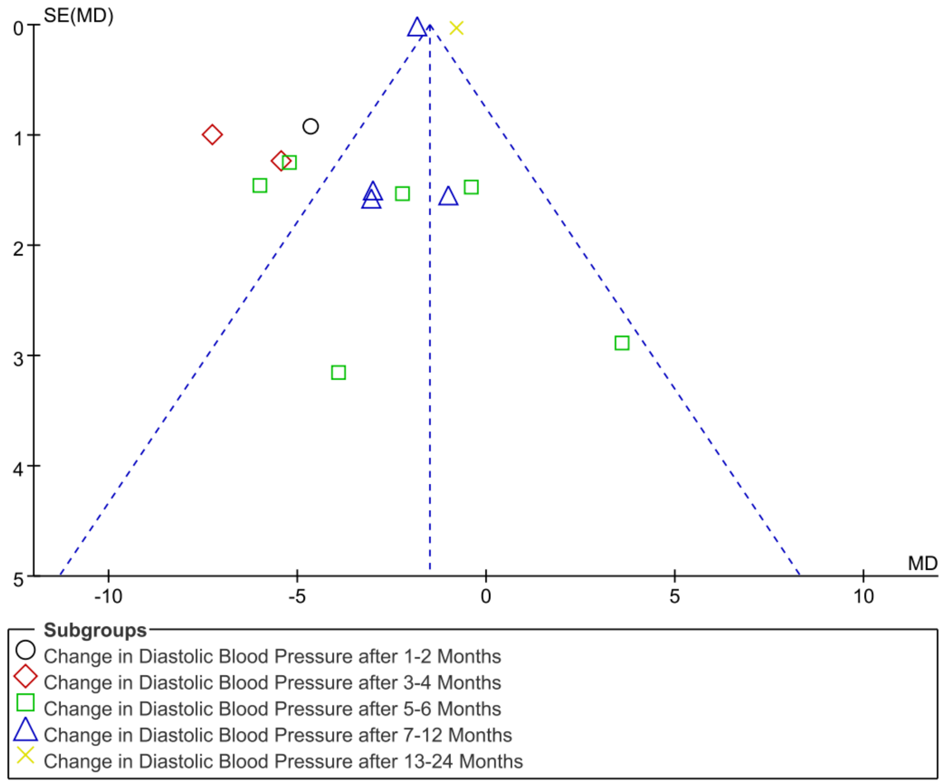

SFigure 11 Funnel Plot of the Change in DBP by Follow-up Time Point

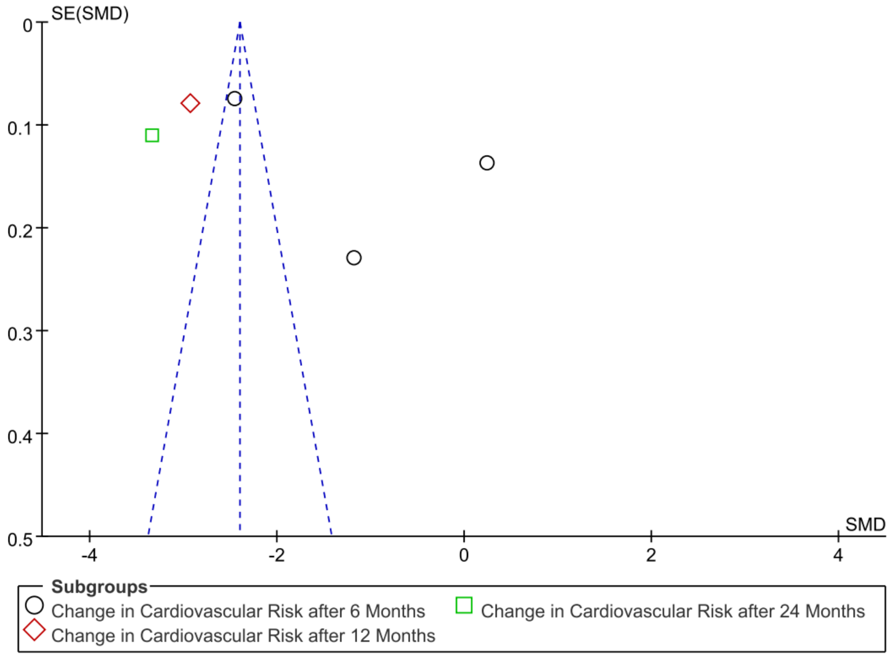

SFigure 12 Funnel Plot of the Change in Cardiovascular Risk by Follow-up Time Point

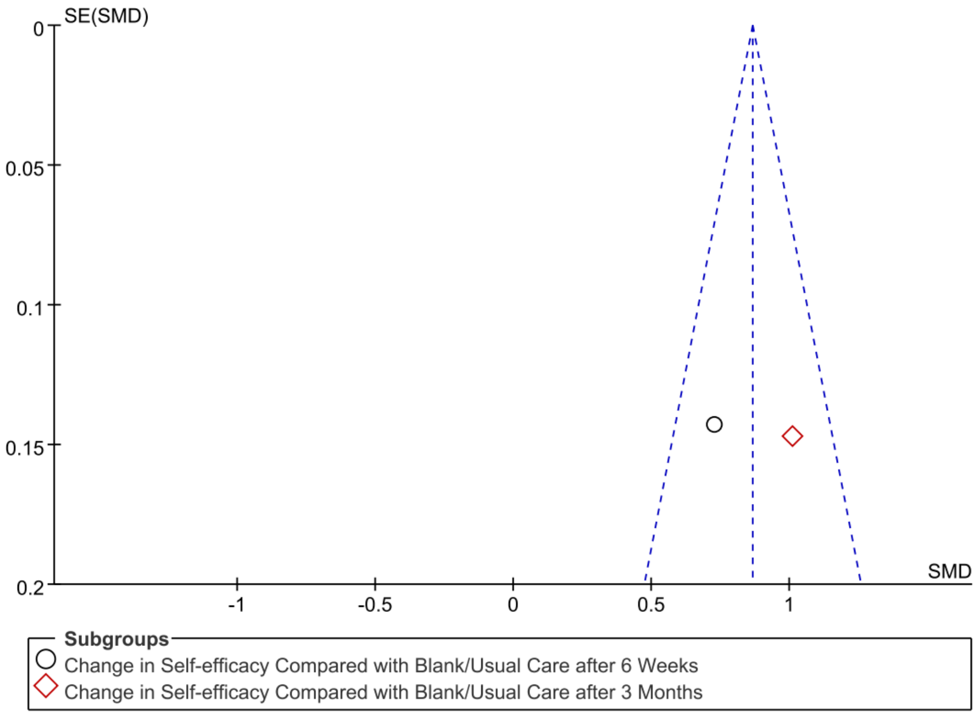

SFigure 13 Funnel Plot of the Change in Blood Pressure Control Self-efficacy by Follow-up Time Point

4.3 GRADE Results - Evidence Profile

Question 1: CDM compared to Blank/Usual Care for hypertension management after 1-2 Month follow-up

Certainty assessment							№ of patients		Effect		Certainty	Importance
№ of studies	Study design	Risk of bias	Inconsistency	Indirectness	Imprecision	Other considerations	CDM	Blank/Usual Care	Relative (95% CI)	Absolute (95% CI)		
**Blood Pressure Control Rate (follow-up: range 1 months to 2 months; assessed with: An automated BP measurement device (Omron U30))**												
1	randomised trials	serious[a]	not serious	not serious	serious[b]	strong association	22/105 (21.0%)	9/105 (8.6%)	**RR 2.44** (1.18 to 5.06)	**123 more per 1,000** (from 15 more to 348 more)	⨁⨁⨁◯ Moderate	

Certainty assessment							№ of patients		Effect		Certainty	Importance
№ of studies	Study design	Risk of bias	Inconsistency	Indirectness	Imprecision	Other considerations	CDM	Blank/Usual Care	Relative (95% CI)	Absolute (95% CI)		
**Change in Systolic Blood Pressure (follow-up: range 1 months to 2 months; assessed with: An automated BP measurement device)**												
1	randomised trials	serious[a]	not serious	not serious	serious[b]	none	105	105	-	SMD **0.62 SD lower** (0.9 lower to 0.35 lower)	⊕⊕◯◯ Low	
**Change in Diastolic Blood Pressure (follow-up: range 1 months to 2 months; assessed with: An automated BP measurement device)**												

Certainty assessment							№ of patients		Effect		Certainty	Importance
№ of studies	Study design	Risk of bias	Inconsistency	Indirectness	Imprecision	Other considerations	CDM	Blank/Usual Care	Relative (95% CI)	Absolute (95% CI)		
1	randomised trials	serious[a]	not serious	not serious	serious[b]	none	105	105	-	SMD **0.69 SD lower** (9.7 lower to 0.41 lower)	⊕⊕◯◯ Low	

Change in Blood Pressure Control Self-efficacy (follow-up: range 1 months to 2 months; assessed with: a self-efficacy scale score)

Certainty assessment							№ of patients		Effect		Certainty	Importance
№ of studies	Study design	Risk of bias	Inconsistency	Indirectness	Imprecision	Other considerations	CDM	Blank/Usual Care	Relative (95% CI)	Absolute (95% CI)		
1	randomised trials	serious[a]	not serious	not serious	serious[b]	strong association	105	105	-	SMD **0.73 SD higher** (0.45 higher to 1.01 higher)	⊕⊕⊕◯ Moderate	

CI: confidence interval; **RR:** risk ratio; **SMD:** standardised mean difference

Explanations

a. Selective outcome reporting

b. The sample size is small and the confidence interval is wide.

Question 2: CDM compared to Blank/Usual Care for hypertension management after 3-4 Month follow-up

Certainty assessment							№ of patients		Effect		Certainty	Importance
№ of studies	Study design	Risk of bias	Inconsistency	Indirectness	Imprecision	Other considerations	CDM	Blank/Usual Care	Relative (95% CI)	Absolute (95% CI)		
**Blood Pressure Control Rate (follow-up: range 3 months to 4 months; assessed with: An automated BP measurement device)**												
3	randomised trials	serious[a]	serious[b,c,d]	not serious	not serious	all plausible residual confounding would reduce the demonstrated effect	195/493 (39.6%)	131/496 (26.4%)	**RR 1.50** (1.25 to 1.79)	**132 more per 1,000** (from 66 more to 209 more)	⊕⊕⊕◯ Moderate	

Certainty assessment							№ of patients		Effect		Certainty	Importance
**№ of studies**	**Study design**	**Risk of bias**	**Inconsistency**	**Indirectness**	**Imprecision**	**Other considerations**	**CDM**	**Blank/Usual Care**	**Relative (95% CI)**	**Absolute (95% CI)**		
**Change in Systolic Blood Pressure (follow-up: range 3 months to 4 months; assessed with: An automated BP measurement device)**												
3	randomised trials	serious[a]	serious[b,c,d]	not serious	not serious	all plausible residual confounding would reduce the demonstrated effect	506	517	-	SMD **0.54 SD lower** (0.67 lower to 0.42 lower)	⨁⨁⨁◯ Moderate	
**Change in Diastolic Blood Pressure (follow-up: range 3 months to 4 months; assessed with: An automated BP measurement device)**												

Certainty assessment							№ of patients		Effect		Certainty	Importance
№ of studies	Study design	Risk of bias	Inconsistency	Indirectness	Imprecision	Other considerations	CDM	Blank/Usual Care	Relative (95% CI)	Absolute (95% CI)		
2	randomised trials	serious[a]	not serious	not serious	serious[e]	all plausible residual confounding would reduce the demonstrated effect	297	315	-	SMD **0.61 SD lower** (0.78 lower to 0.45 lower)	⊕⊕⊕◯ Moderate	

Change in Blood Pressure Control Self-efficacy (follow-up: range 3 months to 4 months; assessed with: a self-efficacy scale)

Certainty assessment							№ of patients		Effect		Certainty	Importance
№ of studies	Study design	Risk of bias	Inconsistency	Indirectness	Imprecision	Other considerations	CDM	Blank/Usual Care	Relative (95% CI)	Absolute (95% CI)		
1	randomised trials	serious[f]	not serious	not serious	serious[e]	strong association	105	105	-	SMD **1.01 SD higher** (0.72 higher to 1.3 higher)	⊕⊕⊕◯ Moderate	

CI: confidence interval; **RR:** risk ratio; **SMD:** standardised mean difference

Explanations

a. Selective outcome reporting;Lack of allocation concealment

b. The mean differences and confidence intervals are distributed on both sides

c. Confidence intervals are uneven

d. The heterogeneity is large

e. The sample size is small and the confidence interval is wide.

f. Selective outcome reporting

Question 3: CDM compared to Blank/Usual Care for hypertension management after 5-6 Month follow-up

Certainty assessment							№ of patients		Effect		Certainty	Importance
№ of studies	Study design	Risk of bias	Inconsistency	Indirectness	Imprecision	Other considerations	CDM	Blank/Usual Care	Relative (95% CI)	Absolute (95% CI)		
**Blood Pressure Control Rate (follow-up: range 5 months to 6 months; assessed with: An automated BP measurement device)**												

Certainty assessment							№ of patients		Effect		Certainty	Importance
№ of studies	Study design	Risk of bias	Inconsistency	Indirectness	Imprecision	Other considerations	CDM	Blank/Usual Care	Relative (95% CI)	Absolute (95% CI)		
8	randomised trials	serious[a,b]	serious[c]	not serious	not serious	all plausible residual confounding would reduce the demonstrated effect	336/668 (50.3%)	234/649 (36.1%)	**RR 1.43** (1.27 to 1.62)	**155 more per 1,000** (from 97 more to 224 more)	⊕⊕⊕◯ Moderate	

Change in Systolic Blood Pressure (follow-up: range 5 months to 6 months; assessed with: An automated BP measurement device)

Certainty assessment							№ of patients		Effect		Certainty	Importance
№ of studies	Study design	Risk of bias	Inconsistency	Indirectness	Imprecision	Other considerations	CDM	Blank/Usual Care	Relative (95% CI)	Absolute (95% CI)		
6	randomised trials	serious[a,b]	serious[c,d]	not serious	not serious	all plausible residual confounding would reduce the demonstrated effect	520	497	-	SMD **0.49 SD lower** (0.61 lower to 0.36 lower)	⨁⨁⨁◯ Moderate	

Change in Diastolic Blood Pressure (follow-up: range 5 months to 6 months; assessed with: An automated BP measurement device)

Certainty assessment							№ of patients		Effect		Certainty	Importance
№ of studies	Study design	Risk of bias	Inconsistency	Indirectness	Imprecision	Other considerations	CDM	Blank/Usual Care	Relative (95% CI)	Absolute (95% CI)		
6	randomised trials	serious[a,b]	serious[c]	not serious	not serious	all plausible residual confounding would reduce the demonstrated effect	520	497	-	SMD **0.33 SD lower** (0.45 lower to 0.2 lower)	⊕⊕⊕◯ Moderate	

Change in Cardiovascular Risk (follow-up: range 5 months to 6 months; assessed with: Framingham 10-year global risk scores;New Zealand Risk Score)

Certainty assessment							№ of patients		Effect		Certainty	Importance
**№ of studies**	**Study design**	**Risk of bias**	**Inconsistency**	**Indirectness**	**Imprecision**	**Other considerations**	**CDM**	**Blank/Usual Care**	**Relative (95% CI)**	**Absolute (95% CI)**		
1 （Perl.S，2016）	randomised trials	serious[e]	not serious	not serious	serious[f]	all plausible residual confounding would reduce the demonstrated effect	120	97	-	SMD **0.25 SD higher** (0.02 lower to 0.52 higher)	⊕⊕⊕◯ Moderate	

CI: confidence interval; **RR:** risk ratio; **SMD:** standardised mean difference

Explanations

a. Selective outcome reporting;Lack of allocation concealment； Deviations from the intended interventions

b. Measurement of the outcome are some concerns

c. The mean differences and confidence intervals are distributed on both sides;Confidence intervals are uneven

d. The heterogeneity is large

e. Randomisation process are some concerns

f. Cross both valid and invalid lines;The sample size is small and the confidence interval is wide.

Question 4: CDM compared to Blank/Usual Care for hypertension management after 7-12 Month follow-up

Certainty assessment							№ of patients		Effect		Certainty	Importance
№ of studies	Study design	Risk of bias	Inconsistency	Indirectness	Imprecision	Other considerations	CDM	Blank/Usual Care	Relative (95% CI)	Absolute (95% CI)		
**Blood Pressure Control Rate (follow-up: range 7 months to 12 months; assessed with: An automated BP measurement device)**												

Certainty assessment							№ of patients		Effect		Certainty	Importance
№ of studies	Study design	Risk of bias	Inconsistency	Indirectness	Imprecision	Other considerations	CDM	Blank/Usual Care	Relative (95% CI)	Absolute (95% CI)		
4	randomised trials	serious [a]	not serious	not serious	not serious	publication bias strongly suspected[b]	1702/3569 (47.7%)	528/1611 (32.8%)	**RR 1.52** (1.40 to 1.64)	**170 more per 1,000** (from 131 more to 210 more)	⨁⨁◯◯ Low	

Change in Systolic Blood Pressure (follow-up: range 7 months to 12 months; assessed with: An automated BP measurement device)

Certainty assessment							№ of patients		Effect		Certainty	Importance
№ of studies	Study design	Risk of bias	Inconsistency	Indirectness	Imprecision	Other considerations	CDM	Blank/Usual Care	Relative (95% CI)	Absolute (95% CI)		
4	randomised trials	serious[a]	serious[c,d]	not serious	not serious	publication bias strongly suspected all plausible residual confounding would reduce the demonstrated effect[b]	3474	1468	-	SMD **3 SD lower** (3.12 lower to 2.88 lower)	⊕⊕◯◯ Low	

Change in Diastolic Blood Pressure (follow-up: range 7 months to 12 months; assessed with: An automated BP measurement device)

Certainty assessment							№ of patients		Effect		Certainty	Importance
№ of studies	Study design	Risk of bias	Inconsistency	Indirectness	Imprecision	Other considerations	CDM	Blank/Usual Care	Relative (95% CI)	Absolute (95% CI)		
4	randomised trials	serious[a]	not serious	not serious	not serious	publication bias strongly suspected[b]	3474	1468	-	SMD **2.02 SD lower** (2.1 lower to 1.94 lower)	⊕⊕◯◯ Low	

CI: confidence interval; **RR:** risk ratio; **SMD:** standardised mean difference

Explanations

a. Lack of allocation concealment；Deviations from the intended interventions;Timing of identification or recruitment of participants;Measurement of the outcome are some concerns

b. The asymmetry of the funnel plot

c. The mean differences and confidence intervals are distributed on both sides;Confidence intervals are uneven

d. The heterogeneity is large

Question 5: CDM compared to Blank/Usual Care for hypertension management after 13-24 Month follow-up

Certainty assessment							№ of patients		Effect		Certainty	Importance
№ of studies	Study design	Risk of bias	Inconsistency	Indirectness	Imprecision	Other considerations	CDM	Blank/Usual Care	Relative (95% CI)	Absolute (95% CI)		
**Blood Pressure Control Rate (follow-up: range 13 months to 24 months; assessed with: An automated BP measurement device)**												

Certainty assessment							№ of patients		Effect		Certainty	Importance
№ of studies	Study design	Risk of bias	Inconsistency	Indirectness	Imprecision	Other considerations	CDM	Blank/Usual Care	Relative (95% CI)	Absolute (95% CI)		
2	randomised trials	serious[a]	not serious	not serious	not serious	publication bias strongly suspected[b]	320/506 (63.2%)	261/506 (51.6%)	**RR 1.23** (1.10 to 1.37)	**119 more per 1,000** (from 52 more to 191 more)	⊕⊕◯◯ Low	

Change in SBP (follow-up: range 13 months to 24 months; assessed with: An automated BP measurement device)

Certainty assessment							№ of patients		Effect		Certainty	Importance
№ of studies	Study design	Risk of bias	Inconsistency	Indirectness	Imprecision	Other considerations	CDM	Blank/Usual Care	Relative (95% CI)	Absolute (95% CI)		
1	randomised trials	serious[a]	not serious	not serious	serious[c]	none	384	382	-	SMD **1.4 SD lower** (1.56 lower to 1.24 lower)	⊕⊕◯◯ Low	

Change in DBP (follow-up: range 13 months to 24 months; assessed with: An automated BP measurement device)

Certainty assessment							№ of patients		Effect		Certainty	Importance
№ of studies	Study design	Risk of bias	Inconsistency	Indirectness	Imprecision	Other considerations	CDM	Blank/Usual Care	Relative (95% CI)	Absolute (95% CI)		
1	randomised trials	serious[a]	not serious	not serious	serious[c]	none	384	382	-	SMD **1.4 SD lower** (1.56 lower to 1.24 lower)	⊕⊕◯◯ Low	

Change in Cardiovascular Risk (follow-up: range 13 months to 24 months; assessed with: FRS 10-year CVD risk score)

Certainty assessment							№ of patients		Effect		Certainty	Importance
№ of studies	Study design	Risk of bias	Inconsistency	Indirectness	Imprecision	Other considerations	CDM	Blank/Usual Care	Relative (95% CI)	Absolute (95% CI)		
1	randomised trials	serious[a]	not serious	not serious	serious[c]	none	393	382	-	SMD **3.33 SD lower** (3.55 lower to 3.11 lower)	⊕⊕◯◯ Low	

CI: confidence interval; **RR:** risk ratio; **SMD:** standardised mean difference

Explanations

a. Selective outcome reporting;Lack of allocation concealment;Timing of identification or recruitment of participants

b. The asymmetry of the funnel plot

c. The sample size is small

Question 6: Comprehensive CDM compared to Only SMS for hypertension management after 6 Month follow-up

Certainty assessment							№ of patients		Effect		Certainty	Importance
**№ of studies**	**Study design**	**Risk of bias**	**Inconsistency**	**Indirectness**	**Imprecision**	**Other considerations**	**CDM**	**Only SMS**	**Relative (95% CI)**	**Absolute (95% CI)**		
**Change in Cardiovascular Risk (follow-up: mean 6 months; assessed with: FRS 10-year CVD risk score)**												
2	randomised trials	serious[a,b]	serious[c,d]	not serious	not serious	all plausible residual confounding would reduce the demonstrated effect	633	720	-	SMD **1.82 SD lower** (3.06 lower to 0.58 lower)	⨁⨁⨁◯ Moderate	

CI: confidence interval; **SMD:** standardised mean difference

Explanations

a. Selective outcome reporting;Lack of allocation concealment； Deviations from the intended interventions

b. Selective outcome reporting and measurement of the outcome are high risk

c. Confidence intervals are uneven

d. The heterogeneity is large

Question 7: Comprehensive CDM compared to Only SMS for hypertension management after 12 Month follow-up

Certainty assessment							№ of patients		Effect		Certainty	Importance
№ of studies	Study design	Risk of bias	Inconsistency	Indirectness	Imprecision	Other considerations	CDM	Only SMS	Relative (95% CI)	Absolute (95% CI)		
**Change in Cardiovascular Risk (follow-up: mean 12 months; assessed with: FRS 10-year CVD risk score)**												

Certainty assessment							№ of patients		Effect		Certainty	Importance
**№ of studies**	**Study design**	**Risk of bias**	**Inconsistency**	**Indirectness**	**Imprecision**	**Other considerations**	**CDM**	**Only SMS**	**Relative (95% CI)**	**Absolute (95% CI)**		
1	randomised trials	serious[a]	not serious	not serious	not serious	all plausible residual confounding would reduce the demonstrated effect	692	607	-	SMD **2.92 SD lower** (3.08 lower to 2.76 lower)	⊕⊕⊕⊕ High	

CI: confidence interval; **SMD:** standardised mean difference

Explanations

a. Measurement of the outcome are some concerns

b. The sample size is small and the confidence interval is wide.

4.4 GRADE Results - Summary of Finding Table

Summary of findings:

1. CDM compared to Blank/Usual Care for hypertension management after 1-2 Month follow-up

Patient or population: hypertension management after 1-2 Month follow-up

Intervention: CDM

Comparison: Blank/Usual Care

Outcomes	**Anticipated absolute effects*** (95% CI)		Relative effect (95% CI)	№ of participants (studies)	Certainty of the evidence (GRADE)	Comments
	**Risk with Blank/Usual Care**	**Risk with CDM**				
Blood Pressure Control Rate (BP Control Rate) assessed with: An automated BP measurement device (Omron U30) follow-up: range 1 months to 2 months	86 per 1,000	**209 per 1,000** (101 to 434)	**RR 2.44** (1.18 to 5.06)	210 (1 RCT)	⨁⨁⨁◯ Moderate[a,b]	

Summary of findings:

1. CDM compared to Blank/Usual Care for hypertension management after 1-2 Month follow-up

Patient or population: hypertension management after 1-2 Month follow-up

Intervention: CDM

Comparison: Blank/Usual Care

Outcomes	**Anticipated absolute effects*** (95% CI)		Relative effect (95% CI)	№ of participants (studies)	Certainty of the evidence (GRADE)	Comments
	**Risk with Blank/Usual Care**	**Risk with CDM**				
Change in Systolic Blood Pressure (Change in SBP)<br>assessed with: An automated BP measurement device<br>follow-up: range 1 months to 2 months	-	SMD **0.62 SD lower**<br>(0.9 lower to 0.35 lower)	-	210<br>(1 RCT)	⊕⊕◯◯<br>Low[a,b]	-

Summary of findings:

1. CDM compared to Blank/Usual Care for hypertension management after 1-2 Month follow-up

Patient or population: hypertension management after 1-2 Month follow-up

Intervention: CDM

Comparison: Blank/Usual Care

Outcomes	**Anticipated absolute effects*** (95% CI)		Relative effect (95% CI)	№ of participants (studies)	Certainty of the evidence (GRADE)	Comments
	**Risk with Blank/Usual Care**	**Risk with CDM**				
Change in Diastolic Blood Pressure (Change in DBP)<br><br>assessed with: An automated BP measurement device<br><br>follow-up: range 1 months to 2 months	-	SMD **0.69 SD lower**<br><br>(9.7 lower to 0.41 lower)	-	210<br><br>(1 RCT)	⨁⨁◯◯<br><br>Low[a,b]	

Summary of findings:

1. CDM compared to Blank/Usual Care for hypertension management after 1-2 Month follow-up

Patient or population: hypertension management after 1-2 Month follow-up

Intervention: CDM

Comparison: Blank/Usual Care

Outcomes	**Anticipated absolute effects*** (95% CI)		Relative effect (95% CI)	№ of participants (studies)	Certainty of the evidence (GRADE)	Comments
	**Risk with Blank/Usual Care**	**Risk with CDM**				
Change in Blood Pressure Control Self-efficacy (Change in BP Control Self-efficacyChange in Blood Pressure Control Self-efficacy)<br><br>assessed with: a self-efficacy scale score<br><br>follow-up: range 1 months to 2 months	-	SMD **0.73 SD higher**<br><br>(0.45 higher to 1.01 higher)	-	210<br><br>(1 RCT)	⨁⨁⨁◯<br><br>Moderate[a,b]	

Summary of findings:

1. CDM compared to Blank/Usual Care for hypertension management after 1-2 Month follow-up

Patient or population: hypertension management after 1-2 Month follow-up

Intervention: CDM

Comparison: Blank/Usual Care

Outcomes	**Anticipated absolute effects*** (95% CI)		Relative effect (95% CI)	№ of participants (studies)	Certainty of the evidence (GRADE)	Comments
	**Risk with Blank/Usual Care**	**Risk with CDM**				

***The risk in the intervention group** (and its 95% confidence interval) is based on the assumed risk in the comparison group and the **relative effect** of the intervention (and its 95% CI).

CI: confidence interval; **RR:** risk ratio; **SMD:** standardised mean difference

Summary of findings:

1. CDM compared to Blank/Usual Care for hypertension management after 1-2 Month follow-up

Patient or population: hypertension management after 1-2 Month follow-up

Intervention: CDM

Comparison: Blank/Usual Care

Outcomes	**Anticipated absolute effects*** (95% CI)		Relative effect (95% CI)	№ of participants (studies)	Certainty of the evidence (GRADE)	Comments
	**Risk with Blank/Usual Care**	**Risk with CDM**				

GRADE Working Group grades of evidence

High certainty: we are very confident that the true effect lies close to that of the estimate of the effect.

Moderate certainty: we are moderately confident in the effect estimate: the true effect is likely to be close to the estimate of the effect, but there is a possibility that it is substantially different.

Low certainty: our confidence in the effect estimate is limited: the true effect may be substantially different from the estimate of the effect.

Very low certainty: we have very little confidence in the effect estimate: the true effect is likely to be substantially different from the estimate of effect.

Explanations

a. Selective outcome reporting

b. The sample size is small and the confidence interval is wide

Summary of findings:

2.CDM compared to Blank/Usual Care for hypertension management after 3-4 Month follow-up

Patient or population: hypertension management after 3-4 Month follow-up

Intervention: CDM

Comparison: Blank/Usual Care

Outcomes	**Anticipated absolute effects*** (95% CI)		Relative effect (95% CI)	№ of participants (studies)	Certainty of the evidence (GRADE)	Comments
	**Risk with Blank/Usual Care**	**Risk with CDM**				
Blood Pressure Control Rate (BP Control Rate) assessed with: An automated BP measurement device follow-up: range 3 months to 4 months	264 per 1,000	**396 per 1,000** (330 to 473)	**RR 1.50** (1.25 to 1.79)	989 (3 RCTs)	⊕⊕⊕◯ Moderate[a,b,c,d]	

Summary of findings:

2.CDM compared to Blank/Usual Care for hypertension management after 3-4 Month follow-up

Patient or population: hypertension management after 3-4 Month follow-up

Intervention: CDM

Comparison: Blank/Usual Care

Outcomes	**Anticipated absolute effects*** (95% CI)		Relative effect (95% CI)	№ of participants (studies)	Certainty of the evidence (GRADE)	Comments
	**Risk with Blank/Usual Care**	**Risk with CDM**				
Change in Systolic Blood Pressure (Change in SBP)<br><br>assessed with: An automated BP measurement device<br><br>follow-up: range 3 months to 4 months	-	SMD **0.54 SD lower**<br><br>(0.67 lower to 0.42 lower)	-	1023<br><br>(3 RCTs)	⨁⨁⨁◯<br><br>Moderate[a,b,c,d]	

Summary of findings:

2.CDM compared to Blank/Usual Care for hypertension management after 3-4 Month follow-up

Patient or population: hypertension management after 3-4 Month follow-up

Intervention: CDM

Comparison: Blank/Usual Care

Outcomes	**Anticipated absolute effects*** (95% CI)		Relative effect (95% CI)	№ of participants (studies)	Certainty of the evidence (GRADE)	Comments
	**Risk with Blank/Usual Care**	**Risk with CDM**				
Change in Diastolic Blood Pressure (Change in DBP)<br><br>assessed with: An automated BP measurement device<br><br>follow-up: range 3 months to 4 months	-	SMD **0.61 SD lower**<br><br>(0.78 lower to 0.45 lower)	-	612<br><br>(2 RCTs)	⊕⊕⊕◯<br><br>Moderate[a,e]	

Summary of findings:

2.CDM compared to Blank/Usual Care for hypertension management after 3-4 Month follow-up

Patient or population: hypertension management after 3-4 Month follow-up

Intervention: CDM

Comparison: Blank/Usual Care

Outcomes	**Anticipated absolute effects*** (95% CI)		Relative effect (95% CI)	№ of participants (studies)	Certainty of the evidence (GRADE)	Comments
	**Risk with Blank/Usual Care**	**Risk with CDM**				
Change in Blood Pressure Control Self-efficacy (Change in BP Control Self-efficacy)<br>assessed with: a self-efficacy scale<br>follow-up: range 3 months to 4 months	-	SMD **1.01 SD higher**<br>(0.72 higher to 1.3 higher)	-	210<br>(1 RCT)	⨁⨁⨁◯<br>Moderate[e,f]	

Summary of findings:

2.CDM compared to Blank/Usual Care for hypertension management after 3-4 Month follow-up

Patient or population: hypertension management after 3-4 Month follow-up

Intervention: CDM

Comparison: Blank/Usual Care

Outcomes	**Anticipated absolute effects*** (95% CI)		Relative effect (95% CI)	№ of participants (studies)	Certainty of the evidence (GRADE)	Comments
	**Risk with Blank/Usual Care**	**Risk with CDM**				

***The risk in the intervention group** (and its 95% confidence interval) is based on the assumed risk in the comparison group and the **relative effect** of the intervention (and its 95% CI).

CI: confidence interval; **RR:** risk ratio; **SMD:** standardised mean difference

Summary of findings:

2.CDM compared to Blank/Usual Care for hypertension management after 3-4 Month follow-up

Patient or population: hypertension management after 3-4 Month follow-up

Intervention: CDM

Comparison: Blank/Usual Care

Outcomes	**Anticipated absolute effects*** (95% CI)		Relative effect (95% CI)	№ of participants (studies)	Certainty of the evidence (GRADE)	Comments
	**Risk with Blank/Usual Care**	**Risk with CDM**				

GRADE Working Group grades of evidence

High certainty: we are very confident that the true effect lies close to that of the estimate of the effect.

Moderate certainty: we are moderately confident in the effect estimate: the true effect is likely to be close to the estimate of the effect, but there is a possibility that it is substantially different.

Low certainty: our confidence in the effect estimate is limited: the true effect may be substantially different from the estimate of the effect.

Very low certainty: we have very little confidence in the effect estimate: the true effect is likely to be substantially different from the estimate of effect.

Explanations

a. Selective outcome reporting;Lack of allocation concealment

b. The mean differences and confidence intervals are distributed on both sides

c. Confidence intervals are uneven

d. The heterogeneity is large

e. The sample size is small and the confidence interval is wide.

f. Selective outcome reportingn

Summary of findings:

3.CDM compared to Blank/Usual Care for hypertension management after 5-6 Month follow-up

Patient or population: hypertension management after 5-6 Month follow-up

Intervention: CDM

Comparison: Blank/Usual Care

Outcomes	**Anticipated absolute effects*** (95% CI)		Relative effect (95% CI)	№ of participants (studies)	Certainty of the evidence (GRADE)	Comments
	**Risk with Blank/Usual Care**	**Risk with CDM**				
Blood Pressure Control Rate (BP Control Rate) assessed with: An automated BP measurement device follow-up: range 5 months to 6 months	361 per 1,000	**516 per 1,000** (458 to 584)	**RR 1.43** (1.27 to 1.62)	1317 (8 RCTs)	⊕⊕⊕◯ Moderate[a,b,c]	

Summary of findings:

3.CDM compared to Blank/Usual Care for hypertension management after 5-6 Month follow-up

Patient or population: hypertension management after 5-6 Month follow-up

Intervention: CDM

Comparison: Blank/Usual Care

Outcomes	**Anticipated absolute effects*** (95% CI)		Relative effect (95% CI)	№ of participants (studies)	Certainty of the evidence (GRADE)	Comments
	**Risk with Blank/Usual Care**	**Risk with CDM**				
Change in Systolic Blood Pressure (Change in SBP)<br>assessed with: An automated BP measurement device<br>follow-up: range 5 months to 6 months	-	SMD **0.49 SD lower**<br>(0.61 lower to 0.36 lower)	-	1017<br>(6 RCTs)	⊕⊕⊕◯<br>Moderate[a,b,c,d]	

Summary of findings:

3.CDM compared to Blank/Usual Care for hypertension management after 5-6 Month follow-up

Patient or population: hypertension management after 5-6 Month follow-up

Intervention: CDM

Comparison: Blank/Usual Care

Outcomes	**Anticipated absolute effects*** (95% CI)		Relative effect (95% CI)	№ of participants (studies)	Certainty of the evidence (GRADE)	Comments
	**Risk with Blank/Usual Care**	**Risk with CDM**				
Change in Diastolic Blood Pressure (Change in DBP)<br><br>assessed with: An automated BP measurement device<br><br>follow-up: range 5 months to 6 months	-	SMD **0.33 SD lower**<br><br>(0.45 lower to 0.2 lower)	-	1017<br><br>(6 RCTs)	⨁⨁⨁◯<br><br>Moderate[a,b,c]	

Summary of findings:

3.CDM compared to Blank/Usual Care for hypertension management after 5-6 Month follow-up

Patient or population: hypertension management after 5-6 Month follow-up

Intervention: CDM

Comparison: Blank/Usual Care

Outcomes	**Anticipated absolute effects*** (95% CI)		Relative effect (95% CI)	№ of participants (studies)	Certainty of the evidence (GRADE)	Comments
	**Risk with Blank/Usual Care**	**Risk with CDM**				
Change in Cardiovascular Risk (Change in Cardiovascular Risk)<br>assessed with: Framingham 10-year global risk scores;New Zealand Risk Score<br>follow-up: range 5 months to 6 months	-	SMD **0.25 SD higher**<br>(0.02 lower to 0.52 higher)	-	217<br>(1 RCT)<br>（Perl.S，2016）	⊕⊕⊕◯<br>Moderate[e,f]	

Summary of findings:

3.CDM compared to Blank/Usual Care for hypertension management after 5-6 Month follow-up

Patient or population: hypertension management after 5-6 Month follow-up

Intervention: CDM

Comparison: Blank/Usual Care

Outcomes	**Anticipated absolute effects*** (95% CI)		Relative effect (95% CI)	№ of participants (studies)	Certainty of the evidence (GRADE)	Comments
	**Risk with Blank/Usual Care**	**Risk with CDM**				

*__The risk in the intervention group__ (and its 95% confidence interval) is based on the assumed risk in the comparison group and the **relative effect** of the intervention (and its 95% CI).

CI: confidence interval; **RR:** risk ratio; **SMD:** standardised mean difference

GRADE Working Group grades of evidence

High certainty: we are very confident that the true effect lies close to that of the estimate of the effect.

Moderate certainty: we are moderately confident in the effect estimate: the true effect is likely to be close to the estimate of the effect, but there is a possibility that it is substantially different.

Low certainty: our confidence in the effect estimate is limited: the true effect may be substantially different from the estimate of the effect.

Very low certainty: we have very little confidence in the effect estimate: the true effect is likely to be substantially different from the estimate of effect.

Explanations

a. Selective outcome reporting;Lack of allocation concealment；Deviations from the intended interventions

b. Measurement of the outcome are some concerns

c. The mean differences and confidence intervals are distributed on both sides;Confidence intervals are uneven

d. The heterogeneity is large

e. Randomisation process are some concerns

f. Cross both valid and invalid lines;The sample size is small and the confidence interval is wide.

Summary of findings:

4.CDM compared to Blank/Usual Care for hypertension management after 7-12 Month follow-up

Patient or population: hypertension management after 7-12 Month follow-up

Intervention: CDM

Comparison: Blank/Usual Care

Outcomes	**Anticipated absolute effects*** (95% CI)		Relative effect (95% CI)	№ of participants (studies)	Certainty of the evidence (GRADE)	Comments
	**Risk with Blank/Usual Care**	**Risk with CDM**				
Blood Pressure Control Rate (BP Control Rate) assessed with: An automated BP measurement device follow-up: range 7 months to 12 months	328 per 1,000	**498 per 1,000** (459 to 538)	**RR 1.52** (1.40 to 1.64)	5180 (4 RCTs)	⊕⊕◯◯ Low[a,b]	

Summary of findings:

4.CDM compared to Blank/Usual Care for hypertension management after 7-12 Month follow-up

Patient or population: hypertension management after 7-12 Month follow-up

Intervention: CDM

Comparison: Blank/Usual Care

Outcomes	**Anticipated absolute effects*** (95% CI)		Relative effect (95% CI)	№ of participants (studies)	Certainty of the evidence (GRADE)	Comments
	**Risk with Blank/Usual Care**	**Risk with CDM**				
Change in Systolic Blood Pressure (Change in SBP)<br><br>assessed with: An automated BP measurement device<br><br>follow-up: range 7 months to 12 months	-	SMD **3 SD lower**<br><br>(3.12 lower to 2.88 lower)	-	4942<br><br>(4 RCTs)	⊕⊕◯◯<br><br>Low[a,b,c,d]	

Summary of findings:

4.CDM compared to Blank/Usual Care for hypertension management after 7-12 Month follow-up

Patient or population: hypertension management after 7-12 Month follow-up

Intervention: CDM

Comparison: Blank/Usual Care

Outcomes	**Anticipated absolute effects*** (95% CI)		Relative effect (95% CI)	№ of participants (studies)	Certainty of the evidence (GRADE)	Comments
	**Risk with Blank/Usual Care**	**Risk with CDM**				
Change in Diastolic Blood Pressure (Change in DBP)<br>assessed with: An automated BP measurement device<br>follow-up: range 7 months to 12 months	-	SMD **2.02 SD lower**<br>(2.1 lower to 1.94 lower)	-	4942<br>(4 RCTs)	⊕⊕◯◯<br>Low[a,b]	

Summary of findings:

4.CDM compared to Blank/Usual Care for hypertension management after 7-12 Month follow-up

Patient or population: hypertension management after 7-12 Month follow-up

Intervention: CDM

Comparison: Blank/Usual Care

Outcomes	**Anticipated absolute effects*** (95% CI)		Relative effect (95% CI)	№ of participants (studies)	Certainty of the evidence (GRADE)	Comments
	**Risk with Blank/Usual Care**	**Risk with CDM**				

*__The risk in the intervention group__ (and its 95% confidence interval) is based on the assumed risk in the comparison group and the **relative effect** of the intervention (and its 95% CI).

CI: confidence interval; **RR:** risk ratio; **SMD:** standardised mean difference

GRADE Working Group grades of evidence

High certainty: we are very confident that the true effect lies close to that of the estimate of the effect.

Moderate certainty: we are moderately confident in the effect estimate: the true effect is likely to be close to the estimate of the effect, but there is a possibility that it is substantially different.

Low certainty: our confidence in the effect estimate is limited: the true effect may be substantially different from the estimate of the effect.

Very low certainty: we have very little confidence in the effect estimate: the true effect is likely to be substantially different from the estimate of effect.

Explanations

a. Lack of allocation concealment； Deviations from the intended interventions;Timing of identification or recruitment of participants;Measurement of the outcome are some concerns

b. The asymmetry of the funnel plot

c. The mean differences and confidence intervals are distributed on both sides;Confidence intervals are uneven

d. The heterogeneity is large

Summary of findings:

5.CDM compared to Blank/Usual Care for hypertension management after 13-24 Month follow-up

Patient or population: hypertension management after 13-24 Month follow-up

Intervention: CDM

Comparison: Blank/Usual Care

Outcomes	**Anticipated absolute effects*** (95% CI)		Relative effect (95% CI)	№ of participants (studies)	Certainty of the evidence (GRADE)	Comments
	**Risk with Blank/Usual Care**	**Risk with CDM**				
Blood Pressure Control Rate (BP Control Rate)<br>assessed with: An automated BP measurement device<br>follow-up: range 13 months to 24 months	516 per 1,000	**634 per 1,000**<br>(567 to 707)	**RR 1.23**<br>(1.10 to 1.37)	1012<br>(2 RCTs)	⨁⨁◯◯<br>Low[a,b]	

Summary of findings:

5.CDM compared to Blank/Usual Care for hypertension management after 13-24 Month follow-up

Patient or population: hypertension management after 13-24 Month follow-up

Intervention: CDM

Comparison: Blank/Usual Care

Outcomes	**Anticipated absolute effects*** (95% CI)		Relative effect (95% CI)	№ of participants (studies)	Certainty of the evidence (GRADE)	Comments
	**Risk with Blank/Usual Care**	**Risk with CDM**				
Change in SBP (Change in SBP)<br>assessed with: An automated BP measurement device<br>follow-up: range 13 months to 24 months	-	SMD **3.71 SD lower**<br>(3.94 lower to 3.48 lower)	-	766<br>(1 RCT)	⊕⊕◯◯<br>Low[a,c]	

Summary of findings:

5.CDM compared to Blank/Usual Care for hypertension management after 13-24 Month follow-up

Patient or population: hypertension management after 13-24 Month follow-up

Intervention: CDM

Comparison: Blank/Usual Care

Outcomes	**Anticipated absolute effects*** (95% CI)		Relative effect (95% CI)	№ of participants (studies)	Certainty of the evidence (GRADE)	Comments
	**Risk with Blank/Usual Care**	**Risk with CDM**				
Change in DBP (Change in DBP)<br>assessed with: An automated BP measurement device<br>follow-up: range 13 months to 24 months	-	SMD **1.4 SD lower**<br>(1.56 lower to 1.24 lower)	-	766<br>(1 RCT)	⨁⨁◯◯<br>Low[a,c]	

Summary of findings:

5.CDM compared to Blank/Usual Care for hypertension management after 13-24 Month follow-up

Patient or population: hypertension management after 13-24 Month follow-up

Intervention: CDM

Comparison: Blank/Usual Care

Outcomes	**Anticipated absolute effects*** (95% CI)		Relative effect (95% CI)	№ of participants (studies)	Certainty of the evidence (GRADE)	Comments
	**Risk with Blank/Usual Care**	**Risk with CDM**				
Change in Cardiovascular Risk (Change in CVD Risk)<br><br>assessed with: FRS 10-year CVD risk score<br><br>follow-up: range 13 months to 24 months	-	SMD **3.33 SD lower**<br><br>(3.55 lower to 3.11 lower)	-	775<br><br>(1 RCT)	⨁⨁◯◯<br><br>Low[a,c]	

Summary of findings:

5.CDM compared to Blank/Usual Care for hypertension management after 13-24 Month follow-up

Patient or population: hypertension management after 13-24 Month follow-up

Intervention: CDM

Comparison: Blank/Usual Care

Outcomes	**Anticipated absolute effects*** (95% CI)		Relative effect (95% CI)	№ of participants (studies)	Certainty of the evidence (GRADE)	Comments
	**Risk with Blank/Usual Care**	**Risk with CDM**				

*__The risk in the intervention group__ (and its 95% confidence interval) is based on the assumed risk in the comparison group and the **relative effect** of the intervention (and its 95% CI).

CI: confidence interval; **RR:** risk ratio; **SMD:** standardised mean difference

Summary of findings:

5.CDM compared to Blank/Usual Care for hypertension management after 13-24 Month follow-up

Patient or population: hypertension management after 13-24 Month follow-up

Intervention: CDM

Comparison: Blank/Usual Care

Outcomes	**Anticipated absolute effects*** (95% CI)		Relative effect (95% CI)	№ of participants (studies)	Certainty of the evidence (GRADE)	Comments
	**Risk with Blank/Usual Care**	**Risk with CDM**				

GRADE Working Group grades of evidence

High certainty: we are very confident that the true effect lies close to that of the estimate of the effect.

Moderate certainty: we are moderately confident in the effect estimate: the true effect is likely to be close to the estimate of the effect, but there is a possibility that it is substantially different.

Low certainty: our confidence in the effect estimate is limited: the true effect may be substantially different from the estimate of the effect.

Very low certainty: we have very little confidence in the effect estimate: the true effect is likely to be substantially different from the estimate of effect.

Explanations

a. Selective outcome reporting;Lack of allocation concealment;Timing of identification or recruitment of participants

b. The asymmetry of the funnel plot

c. The sample size is small

Summary of findings:

6.Comprehensive CDM compared to Only SMS for hypertension management after 6 Month follow-up

Patient or population: hypertension management after 6 Month follow-up

Intervention: Comprehensive CDM

Comparison: Only SMS

Outcomes	**Anticipated absolute effects*** (95% CI)		Relative effect (95% CI)	№ of participants (studies)	Certainty of the evidence (GRADE)	Comments
	**Risk with Only SMS**	**Risk with CDM**				
Change in Cardiovascular Risk (Change in CVD Risk)<br><br>assessed with: FRS 10-year CVD risk score<br><br>follow-up: mean 6 months	-	SMD **1.82 SD lower**<br><br>(3.06 lower to 0.58 lower)	-	1353<br><br>(2 RCTs)	⊕⊕⊕◯<br><br>Moderate[a,b,c,d]	

Summary of findings:

6.Comprehensive CDM compared to Only SMS for hypertension management after 6 Month follow-up

Patient or population: hypertension management after 6 Month follow-up

Intervention: Comprehensive CDM

Comparison: Only SMS

Outcomes	**Anticipated absolute effects*** (95% CI)		Relative effect (95% CI)	№ of participants (studies)	Certainty of the evidence (GRADE)	Comments
	**Risk with Only SMS**	**Risk with CDM**				

*__The risk in the intervention group__ (and its 95% confidence interval) is based on the assumed risk in the comparison group and the **relative effect** of the intervention (and its 95% CI).

CI: confidence interval; **SMD:** standardised mean difference

Summary of findings:

6.Comprehensive CDM compared to Only SMS for hypertension management after 6 Month follow-up

Patient or population: hypertension management after 6 Month follow-up

Intervention: Comprehensive CDM

Comparison: Only SMS

Outcomes	**Anticipated absolute effects*** (95% CI)		Relative effect (95% CI)	№ of participants (studies)	Certainty of the evidence (GRADE)	Comments
	**Risk with Only SMS**	**Risk with CDM**				

GRADE Working Group grades of evidence

High certainty: we are very confident that the true effect lies close to that of the estimate of the effect.

Moderate certainty: we are moderately confident in the effect estimate: the true effect is likely to be close to the estimate of the effect, but there is a possibility that it is substantially different.

Low certainty: our confidence in the effect estimate is limited: the true effect may be substantially different from the estimate of the effect.

Very low certainty: we have very little confidence in the effect estimate: the true effect is likely to be substantially different from the estimate of effect.

Explanations

a. Selective outcome reporting;Lack of allocation concealment；Deviations from the intended interventions

b. Selective outcome reporting and measurement of the outcome are high risk

c. Confidence intervals are uneven

d. The heterogeneity is large

Summary of findings:

7.Comprehensive CDM compared to Only SMS for hypertension management after 12 Month follow-up

Patient or population: hypertension management after 12 Month follow-up

Intervention: Comprehensive CDM

Comparison: Only SMS

Outcomes	**Anticipated absolute effects*** (95% CI)		Relative effect (95% CI)	№ of participants (studies)	Certainty of the evidence (GRADE)	Comments
	**Risk with Only SMS**	**Risk with CDM**				
Change in Cardiovascular Risk (Change in CVD Risk)<br>assessed with: FRS 10-year CVD risk score<br>follow-up: mean 12 months	-	SMD **2.92 SD lower**<br>(3.08 lower to 2.76 lower)	-	1299<br>(1 RCT)	⨁⨁⨁⨁<br>High[a]	

Summary of findings:

7.Comprehensive CDM compared to Only SMS for hypertension management after 12 Month follow-up

Patient or population: hypertension management after 12 Month follow-up

Intervention: Comprehensive CDM

Comparison: Only SMS

Outcomes	**Anticipated absolute effects*** (95% CI)		Relative effect (95% CI)	№ of participants (studies)	Certainty of the evidence (GRADE)	Comments
	**Risk with Only SMS**	**Risk with CDM**				

*__The risk in the intervention group__ (and its 95% confidence interval) is based on the assumed risk in the comparison group and the **relative effect** of the intervention (and its 95% CI).

CI: confidence interval; **SMD:** standardised mean difference

Summary of findings:

7.Comprehensive CDM compared to Only SMS for hypertension management after 12 Month follow-up

Patient or population: hypertension management after 12 Month follow-up

Intervention: Comprehensive CDM

Comparison: Only SMS

Outcomes	**Anticipated absolute effects*** (95% CI)		Relative effect (95% CI)	№ of participants (studies)	Certainty of the evidence (GRADE)	Comments
	**Risk with Only SMS**	**Risk with CDM**				

GRADE Working Group grades of evidence

High certainty: we are very confident that the true effect lies close to that of the estimate of the effect.

Moderate certainty: we are moderately confident in the effect estimate: the true effect is likely to be close to the estimate of the effect, but there is a possibility that it is substantially different.

Low certainty: our confidence in the effect estimate is limited: the true effect may be substantially different from the estimate of the effect.

Very low certainty: we have very little confidence in the effect estimate: the true effect is likely to be substantially different from the estimate of effect.

Explanations

a. Measurement of the outcome are some concerns

Reference

[1] Puhan, M. A., Schünemann, H. J., Murad, M. H., Li, T., Brignardello-Petersen, R., Singh, J. A., Kessels, A. G., Guyatt, G. H., & GRADE Working Group (2014). A GRADE Working Group approach for rating the quality of treatment effect estimates from network meta-analysis. BMJ (Clinical research ed.), 349, g5630. https://doi.org/10.1136/bmj.g5630
[2] Guyatt, G., Oxman, A. D., Akl, E. A., Kunz, R., Vist, G., Brozek, J., Norris, S., Falck-Ytter, Y., Glasziou, P., DeBeer, H., Jaeschke, R., Rind, D., Meerpohl, J., Dahm, P., & Schünemann, H. J. (2011). GRADE guidelines: 1. Introduction-GRADE evidence profiles and summary of findings tables. Journal of clinical epidemiology, 64(4), 383–394. https://doi.org/10.1016/j.jclinepi.2010.04.026.
[3] Guyatt, G.H., Oxman, A.D., Kunz, R., Atkins, D., Brozek, J., Vist, G., Alderson, P., Glasziou, P., Falck-Ytter, Y., Schünemann, H.J.. (2011). GRADE guidelines: 2. Framing the question and deciding on important outcomes. Journal of Clinical Epidemiology, 2011. 64(4): p. 395-400.
[4] Balshem H, Helfand M, Schünemann HJ, Oxman AD, Kunz R, Brozek J, et al. (2011). GRADE guidelines: 3. Rating the quality of evidence. J Clin Epidemiol 2011;64:401–6.
[5] Guyatt GH, Oxman AD, Vist G, Kunz R, Brozek J, Alonso-Coello P, et al. (2011). GRADE guidelines: 4. Rating the quality of evidence--study limitations (risk of bias). J Clin Epidemiol 2011;64:407–15.
[6] Guyatt GH, Oxman AD, Montori V, Vist G, Kunz R, Brozek J, et al. (2011). GRADE guidelines: 5. Rating the quality of evidence--publication bias. J Clin Epidemiol 2011;64:1277–82.
[7] Guyatt, G. H., Oxman, A. D., Kunz, R., Brozek, J., Alonso-Coello, P., Rind, D., Devereaux, P. J., Montori, V. M., Freyschuss, B., Vist, G., Jaeschke, R., Williams, J. W., Jr, Murad, M. H., Sinclair, D., Falck-Ytter, Y., Meerpohl, J., Whittington, C., Thorlund, K., Andrews, J., & Schünemann, H. J. (2011). GRADE guidelines 6. Rating the quality of evidence--imprecision. Journal of clinical epidemiology, 64(12), 1283–1293. https://doi.org/10.1016/j.jclinepi.2011.01.012
[8] Guyatt GH, Oxman AD, Kunz R, , Woodcock J, Brozek J, Helfand M, et al. (2011). GRADE guidelines: 7. Rating the quality of evidence--inconsistency. J Clin Epidemiol 2011;64:1294-302.

[9] Guyatt GH, Oxman AD, Kunz R, Woodcock J, Brozek J, Helfand M, et al. GRADE guidelines: 8. Rating the quality of evidence--indirectness. J Clin Epidemiol 2011;64:1303-10.
[10] Guyatt, G. H., Oxman, A. D., Sultan, S., Glasziou, P., Akl, E. A., Alonso-Coello, P., Atkins, D., Kunz, R., Brozek, J., Montori, V., Jaeschke, R., Rind, D., Dahm, P., Meerpohl, J., Vist, G., Berliner, E., Norris, S., Falck-Ytter, Y., Murad, M. H., Schünemann, H. J., … GRADE Working Group (2011). GRADE guidelines: 9. Rating up the quality of evidence. Journal of clinical epidemiology, 64(12), 1311–1316. https://doi.org/10.1016/j.jclinepi.2011.06.004
