# Supplementary material for: The effectiveness of the chronic disease management for hypertension: A systematic review and meta-analysis
Source: Medicine (Baltimore). 2025 Jul 25;104(30):e42455. doi: 10.1097/MD.0000000000042455 (PMC12303511; doi:10.1097/MD.0000000000042455)
Supplement: Supplementary file 3 [file medi-104-e42455-s003.pdf]

PRISMA 2020 flow diagram for new systematic reviews which included searches of databases and registers only

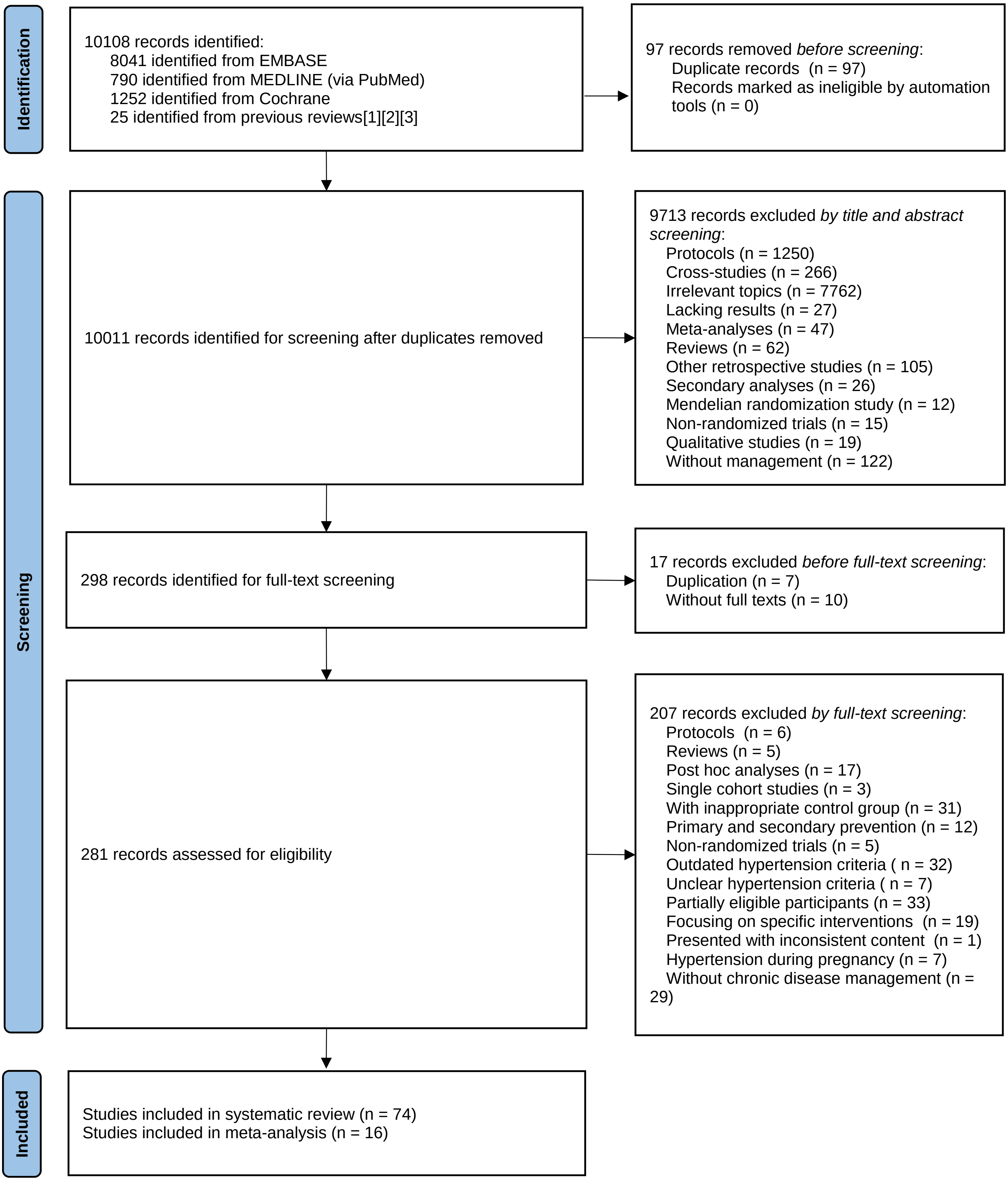

Figure – PRISMA flowchart. PRISMA = Preferred Reporting Items for Systematic Reviews and Meta-analyses; CNKI = China National Knowledge Infrastructure; Wanfang = Wanfang Data, CQVIP = CQVIP Database, EMBASE = Excerpta Medica dataBASE, CENTRAL = Cochrane Central Register of Controlled Trials, MEDLINE =Medical Literature Analysis and Retrieval System Online, PubMed = Public/Publisher MEDLINE (NLM journal articles database).

PRISMA 2020 flow diagram for new systematic reviews which included searches of databases and registers only

From: Page MJ, McKenzie JE, Bossuyt PM, Boutron I, Hoffmann TC, Mulrow CD, et al. The PRISMA 2020 statement: an updated guideline for reporting systematic reviews. BMJ 2021;372:n71. doi: 10.1136/bmj.n71

[1]McLean G, Band R, Saunderson K, et al. Digital interventions to promote self-management in adults with hypertension systematic review and meta-analysis[J]. Journal of hypertension, 2016, 34(4): 600.
[2]Massimi A, De Vito C, Brufola I, et al. Are community-based nurse-led self-management support interventions effective in chronic patients? Results of a systematic review and meta-analysis[J]. PloS one, 2017, 12(3): e0173617.
[3]Li R, Liang N, Bu F, et al. The effectiveness of self-management of hypertension in adults using mobile health: systematic review and meta-analysis[J]. JMIR mHealth and uHealth, 2020, 8(3): e17776.
